# Structure and Dynamics of *Burkholderia pseudomallei* OXA-57, a Distinctive Low-Efficiency Class D β‑Lactamase with Carbapenem-Hydrolyzing Activity

**DOI:** 10.1021/acs.biochem.6c00668

**Published:** 2026-10-22

**Authors:** Éilís C. Bragginton, Charlotte K. Colenso, Michael Beer, Karina Calvopiña, Philip Hinchliffe, John M. Shaw, Catherine. L. Tooke, Rathanin Seng, Narisara Chantratita, Adrian J. Mulholland, Christopher J. Schofield, James Spencer

**Affiliations:** † School of Biochemistry and Biomedical Sciences, 1980 University of Bristol, Bristol BS8 1TD, U.K.; ‡ Diamond Light Source, Harwell Science and Innovation Campus, Didcot OX11 0DE, U.K.; § Centre for Computational Chemistry, School of Chemistry, 1980 University of Bristol, Bristol BS8 1TS, U.K.; ∥ Chemistry Research Laboratory, Department of Chemistry and the Ineos Oxford Institute for Antimicrobial Research, 6396 University of Oxford, Oxford OX1 3TA, U.K.; ⊥ Department of Life Sciences, University of Bath, Bath BA2 7AY, U.K.; # Department of Microbiology and Immunology, Faculty of Tropical Medicine, 115374 Mahidol University, Bangkok 10400, Thailand; ∇ Department of Medical Science, Mahidol University, Amnat Charoen Campus, Amnat Charoen 37000, Thailand; ○ Mahidol-Oxford Tropical Medicine Research Unit, Faculty of Tropical Medicine, Mahidol University, Bangkok 10400, Thailand; ◆ The Academy of Science, The Royal Society of Thailand, Bangkok 10300, Thailand

## Abstract

The Gram-negative bacterium *Burkholderia pseudomallei* causes the severe disease melioidosis. β-Lactams, including carbapenems, are the primary treatment for *B. pseudomallei* infections, but are susceptible to chromosomal serine β-lactamases (SBLs), including the class D enzyme OXA-57. Here we show that recombinant OXA-57 is active toward penicillins and first-generation cephalosporins, slowly hydrolyzes carbapenems, including imipenem and meropenem, but is inactive toward oxyimino-cephalosporins (e.g., ceftazidime). Unlike many OXA enzymes, OXA-57 is sensitive to the mechanism-based SBL inhibitor clavulanic acid, but less so to the diazabicyclooctane avibactam and not to the cyclic boronate vaborbactam. Crystal structures of covalent OXA-57:avibactam and OXA-57:meropenem complexes reveal limited hydrogen-bonding interactions with bound ligands. In molecular dynamics simulations, the bound meropenem acyl-enzyme is mobile, with the 6α-hydroxyethyl group able to sample different orientations, while the water molecule necessary for deacylation is bound only transiently. These observations are consistent with the low level of meropenem turnover, supporting proposals that limited carbapenemase activity is a general property of OXA β-lactamases, and highlight the potential importance of OXA-57 in *B. pseudomallei* β-lactam resistance.

## Graphical abstract

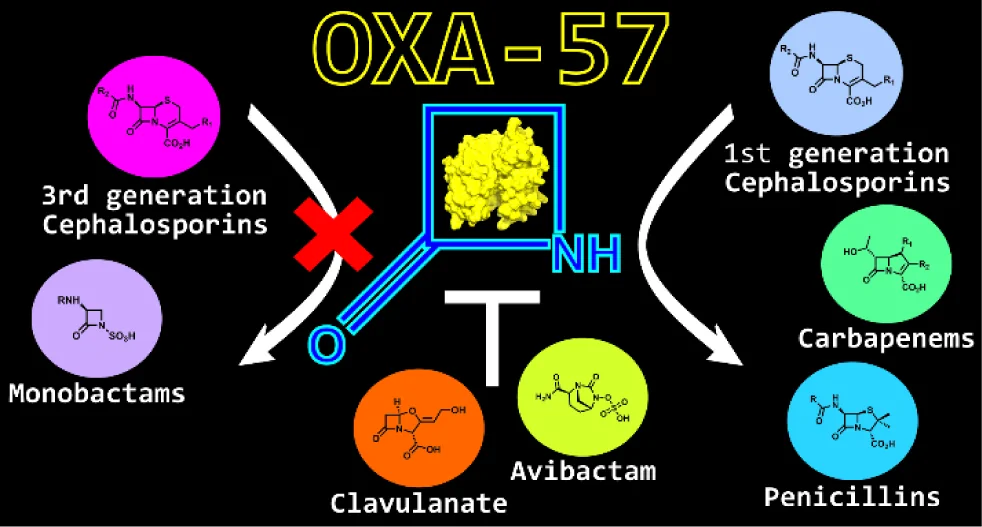

## Introduction

*Burkholderia pseudomallei* is a Gram-negative intracellular pathogen that causes melioidosis, a severe disease endemic to Southeast Asia and Northern Australia.^1^ *B. pseudomallei* infection occurs through multiple routes, including percutaneous inoculation, inhalation and ingestion^1,2^ and presents as an array of symptoms including soft tissue lesions, fever, pneumonia and life threatening sepsis.^3,4^ Melioidosis causes an estimated 89 000 deaths annually with a fatality rate of up to 50% in endemic regions.^5^ The high morbidity and mortality associated with infection classes *B. pseudomallei* as a category B biological agent with potential use as a weapon in bioterrorism.^6^ Survivors of acute melioidosis are susceptible to recurrent *B. pseudomallei* infection and those with preexisting risk factors, such as diabetes mellitus, account for approximately 80% of patients.^1,5^

*B. pseudomallei* manifests intrinsic resistance to a range of antibiotics, including penicillins, 1st and 2nd generation cephalosporins, colistin, macrolides, aminoglycosides and rifamycins.^7^ As a result, the treatment options for melioidosis are limited, with β-lactams such as carbapenems and the 3rd generation cephalosporin ceftazidime being the primary therapeutic choices. Treatment involves an initial intensive phase of intravenous β-lactam antibiotics, usually meropenem or ceftazidime, over 10–14 days, followed by a longer course of oral antibiotics. The latter is classed as the eradication phase of treatment with therapy lasting at least 3 months using combination therapies such as trimethoprim/sulfamethoxazole or amoxicillin/clavulanate.^1,7^

To date, resistance to the clinically used β-lactam antibiotics in *B. pseudomallei* is rare. However, as with most human bacterial infections, cases of drug-resistant melioidosis are increasing due to increased antibiotic use.^8,9^ There are multiple mechanisms of resistance to β-lactam antibiotics, including efflux and decreased porin expression. However, in Gram-negative pathogens, β-lactam degradation by β-lactamases is the most common mechanism.^10^ Currently, >12, 700 β-lactamases have been identified (www.bldb.eu);^11^ they are categorized by their mechanism of action into serine β-lactamases (SBLs) that utilize an active site serine nucleophile to catalyze β-lactam hydrolysis through formation of a covalent acyl-enzyme, and metallo β-lactamases (MBLs), where zinc ions activate a nucleophilic water molecule.^12^ They can be further classified, based on amino acid sequence, into three classes of SBLs (A, C and D) and the class B MBLs.^13^

A notable property of the class-D (OXA) SBLs, differentiating them from class A and C SBLs, is the requirement for a carbamylated lysine for both the acylation and deacylation steps of hydrolysis^14^ (Figure S1). This reversible post-translational modification is driven by reaction with atmospherically-derived CO_2_ and is facilitated by the hydrophobicity of the active site, which modifies the lysine p*K*_*a*_ to favor carbamylation.^14,15^ Some class D SBLs are also distinguished by their hydrolytic activity toward carbapenems, which more usually form long-lived inhibitory acyl-enzyme complexes on reaction with SBLs.^16,17^ However, these carbapenem-hydrolyzing class-D β-lactamases (CHDLs) are distinguished from other SBLs with activity toward carbapenems by their employment of an alternative mechanism of carbapenem degradation resulting in the formation of β-lactone products.^18^ CHDLs are important contributors to carbapenem resistance in several species of pathogenic Gram-negative bacteria, including *Acinetobacter baumannii*.^19^

Previous analysis of the *B. pseudomallei* genome identified 7 potential β-lactamases, 2 of which are described as serine-β-lactamases and 5 as putative metallo-β-lactamases.^20^ To date, however, only the serine-β-lactamases PenI and OXA-57 have been characterized and shown to have β-lactamase activity.^21,22^ PenI, a class A extended spectrum β-lactamase (ESBL), has been extensively studied.^22,23^ Previous investigations have identified PenI mutations, for example P167S^8^ and C69Y, as the cause of ceftazidime-resistant phenotypes in clinical *B. pseudomallei* isolates.^9,24–26^ Similarly, the S72F PenI substitution results in resistance to the widely used β-lactamase inhibitor clavulanic acid.^9,25^ However, although there is clear evidence implicating PenI as a contributing factor to ceftazidime resistance within the clinic, PenI does not catalyze carbapenem turnover^22^ and the cause of carbapenem resistance in *B. pseudomallei* clinical isolates remains elusive.

By contrast with PenI, few reported studies have investigated the possible involvement of the other validated SBL present in the *B. pseudomallei* genome, i.e., the Class-D enzyme OXA-57 (Uniprot Q6ZZZ2), in *B. pseudomallei* resistance phenotypes. A previous biochemical study of OXA-57 was limited to establishing its hydrolytic activity against penicillins and 1st generation cephalosporins.^21^ Here we report a more extensive kinetic characterization of OXA-57 using a broader range of β-lactam substrates with a focus on those of current clinical relevance, and investigate its susceptibility to a range of SBL inhibitors, including clavulanic acid and those (avibactam, vaborbactam) more recently approved for clinical use. We present crystal structures of OXA-57 and its covalent complexes with the carbapenem meropenem and the inhibitor avibactam; and on the basis of these structural data apply extended molecular dynamics simulations to study the behavior of the meropenem-derived acyl-enzyme complex.

## Materials and Experimental Details

Reagents were purchased from Merck unless otherwise specified.

### OXA-57 Purification

DNA encoding for the predicted mature polypeptide of OXA-57 (codons 24 to 269 of the BPSS1997 gene from the *B. pseudomallei* K96243 genome) was cloned into the pET28b T7 expression vector (Novagen) (Table S1) and expressed in *Escherichia coli* BL21 (DE3) cells (Novagen). Cells carrying this plasmid were grown in LB broth, supplemented with 50 μg ml^–1^ kanamycin, at 37 °C to an optical density of 0.6 at 600 nm (OD_600_). Cells were subsequently grown at 18 °C overnight with the addition of 1 mM isopropyl β-D-1-thiogalactopyranoside (IPTG) to induce expression. Cells were harvested by centrifugation (2 700 × *g*, 15 min) and resuspended in 50 mM Tris pH 8.0, 20 mM imidazole pH 8.0, 500 mM NaCl with complete EDTA-free protease inhibitor (Roche). Cells were lysed by 3 passages through a French press cell disruptor (900 psi) and cell debris removed by centrifugation (50 000 × *g*, 1 h, 4 °C). The supernatant was passed through a 0.45 μm filter prior to loading onto a pre-equilibrated 5 mL HisTrap column (Cytiva Life Sciences). The column was equilibrated and washed with 10 column volumes of 50 mM Tris pH 8.0, 20 mM imidazole pH 8.0, 50 mM NaCl and eluted with a linear imidazole gradient (20–300 mM) over 36 column volumes. The purity of eluted fractions was assessed by SDS-PAGE. Fractions containing OXA-57 were pooled and buffer exchanged into 20 mM Tris pH 8.4, 150 mM NaCl, 2.5mM CaCl_2_ in an Amicon 10-kDa molecular weight cutoff (MWCO) centrifugal filter. The N-terminal His-tag was removed from OXA-57 by addition of thrombin (15 μL, 50× units) (Novagen) and incubated overnight at room temperature. Cleaved OXA-57 was loaded onto a HiLoad 16/600 Superdex 75 pg column (Cytiva Life Sciences), pre-equilibrated in 50 mM HEPES pH 7.5, 150 mM NaCl, and peak fractions assessed by SDS-PAGE. Fractions with >90% purity were pooled and concentrated to 15 mg ml^–1^ using an Amicon 10-kDa molecular weight cutoff (MWCO) centrifugal filter. The concentration of OXA-57 was determined by measuring absorbance at λ_280_ nm using the protein extinction coefficient (ε = 56,045 M^–1^ cm^–1^) calculated using the ExPASy ProtParam tool. Purified OXA-57 was stored in 50 mM HEPES pH 7.5, 150 mM NaCl supplemented with 20% glycerol at −70 °C.

### Enzyme Assays

Enzyme assays for all substrates except FC5 ^27^ were followed at 25 °C in 100 mM sodium phosphate pH 7.0, 150 mM NaCHO_3_ (Buffer A) with 1 μM OXA-57. Steady-state kinetic parameters were determined with an Tecan Infinite M200 pro plate reader (Tecan) using Greiner half area 96-well plates. Hydrolysis rates were measured using the following extinction coefficients (Δε) and concentration ranges: ampicillin Δε, −809 M^–1^ cm^–1^ at 235 nm (37.5–600 μM), aztreonam Δε, −700 M^–1^ cm^–1^ at 320 nm (37.5–600 μM), cephalothin Δε, −8790 M^–1^ cm^–1^ at 265 nm (9.4–300 μM), cefotaxime Δε, −7250 M^–1^ cm^–1^ at 265 nm (18.8–300 μM), ceftazidime Δε, −7455 M^–1^ cm^–1^ at 265 nm (18.8–300 μM), imipenem Δε, −10930 M^–1^ cm^–1^ at 298 nm (9.4–300 μM), meropenem Δε, −7200 M^–1^ cm^–1^ at 298 nm (9.4–300 μM). Steady-state velocities were calculated from the initial rates of substrate hydrolysis and plotted against substrate concentration. Kinetic parameters were calculated and analyzed using GraphPad Prism 9.0. Nonlinear regression of the data determined the *K*_*M*_ and *k*_*cat*_ as described by the Michaelis–Menten equation:

1 $$v=\frac{V_{max}\left[S\right]}{K_{M}+\left[S\right]}$$

Where *V*_*max*_ can be described as a function of *k*_*cat*_ multiplied by the enzyme concentration [E]:

2 $$V_{max}=k_{cat}\times \left[E\right]$$

For some substrates, inhibition was observed at high substrate concentrations. In these cases, a modified Michaelis–Menten equation was used to determine the kinetic parameters considering substrate inhibition:

3 $$v=\frac{V_{max}\left[S\right]}{\left(K_{M}+\left[S\right]\right)\times \left(1+\frac{\left[S\right]}{k_{i}}\right)}$$

Steady-state kinetic parameters for the fluorogenic substrate FC5 ^27^ were determined using 12.5 nM OXA-57 in 100 mM phosphate buffer, pH 7.4, 0.01% v/v Triton X-100, 150 mM NaHCO_3_ (Buffer B) over a concentration range of 0.009–20 μM. The assays were conducted at room temperature in clear-bottomed Greiner 384 black well microplates and fluorescence intensity was monitored at λex = 380 nm and λem = 460 nm in a CLARIOstar or PHERAstar FS microplate reader (BMG LabTech). Kinetic parameters were calculated as described above.

The IC_50_ values of inhibitors were determined using a well-described fluorogenic assay by monitoring the enzymatic breakdown of FC5.^27^ In brief, inhibitor compounds at concentrations ranging from 2 × 10^–4^ – 4 mM were incubated with OXA-57 (12.5 nM) in buffer B for 10, 30 and 60 min, followed by the addition of FC5 (5 μM). The assays were conducted as described above for the determination of FC5 kinetic parameters. Normalized data were fitted using a four-parameter function: log­(inhibitor) vs. response, variable slope in GraphPad Prism 6 to obtain IC_50_ values.

### Crystallization and Structure Refinement

OXA-57 was crystallized using the vapor diffusion method. Sitting drops were prepared in a 1:1 ratio of crystallization reagent to 10 mg ml^–1^ OXA-57 in 50 mM HEPES pH 7.5, 150 mM NaCl.

The 2.0 Å uncomplexed OXA-57 structure, OXA-57:meropenem and OXA-57:avibactam complex structures (PDB accessions: 9HPT, 9HPW and 9HPY, respectively) were crystallized in 0.12 M monosaccharides (0.2 M D-glucose, 0.2 M D-mannose, 0.2 M D-galactose, 0.2 M L-fucose, 0.2 M D-xylose, 0.2 M N-acetyl-D-glucosamine), 0.1 M Tris; BICINE pH 8.5, 37.5% (v/v) precipitant mix (25% (v/v) MPD, 25% (w/v) PEG 1000, 35% (w/v) PEG 3350) (from the Morpheus crystallization screen,^28^ Molecular Dimensions). Drops (2 μl protein solution + 2 μl reservoir solution) were equilibrated against 500 μl reservoir solution in CrysChem 24-well plates (Hampton Research) and incubated at 18 °C. The 1.8 Å uncomplexed OXA-57 structure (PDB: 9HPU) crystallized in 0.2 M ammonium acetate, 0.1 M Bis-Tris: HCl pH 6.5, 25% (w/v) PEG 3350 (TOP96, Anatrace). Drops (0.2 μl protein solution + 0.2 μl reservoir solution) were prepared in MRC 2 Lens crystallization plates (SWISSCI) and incubated as above. Both crystallization conditions provided effective cryoprotection, allowing for crystal freezing direct from mother liquor.

For inhibitor and substrate soaking, the 2.0 Å uncomplexed OXA-57 crystals were soaked in mother liquor supplemented with 2 mM avibactam or 10 mM meropenem. All crystals were exposed to mother liquor and flash-cooled in liquid nitrogen prior to data collection. Diffraction data for the 2.0 Å uncomplexed OXA-57 crystals were collected at the ALBA Synchrotron on beamline BL13 - XALOC. Images were indexed and integrated with XDS^29^ and scaled using Aimless^30^ as part of the CCP4 suite.^31^ For the 1.8 Å uncomplexed, inhibitor- and substrate-soaked crystals, diffraction data were collected at Diamond Light Source on beamlines I04 and I03 respectively. Images were indexed and integrated using the DIALS^32^ and Xia2 ^33^ pipeline at Diamond Light Source and subsequently scaled in Aimless. All data were phased by molecular replacement using Phaser.^34^ For the 2.0 Å uncomplexed OXA-57 structure, the PDB identifier 6NI0 (YdxI from *Burkholderia thailandensis* (also known as OXA-D84,^11,35^ ) 90% sequence identity (Table S5)) was used as the search model for molecular replacement. For the 1.8 Å uncomplexed, inhibitor- and substrate-soaked data, the 2.0 Å uncomplexed OXA-57 structure was used. The resulting models were iteratively refined using phenix.refine^36^ and manual model rebuilding in WinCoot7.^37^ Geometry restraints for avibactam and meropenem were calculated using eLBOW and omit maps generated in phenix^36^ from the final model in the absence of ligand.

### Molecular Modeling and Simulations

Unstructured regions of the uncomplexed OXA-57 (PDB entry 9HPT, resolution 2.05 Å) and OXA-57:meropenem complex (PDB entry 9HPW resolution 2.42 Å) X-ray crystal structures (loops β4−β5 and β6−α8, and α1−α2, β4−β5 and β6−α8, respectively), were built using MODELLER v9.24.^38^ In the case of the β4−β5 loop, the X-ray crystal structure of the class D β-lactamase Ybxl (*Burkholderia thailandensis* *)* was used as a template since this region is defined (PDB entry 6NI0, resolution 2.30 Å; 100% sequence identity between OXA-57 residues L209-R214). PROCHECK was used to check the quality of the resulting structures.^39^ Protonation states of titratable groups were predicted using the H++ server v3.2 (http://biophysics.cs.vt.edu/H++)^40^ at pH7.5. For the acyl-enzyme complex, partial atomic charges for the Δ^2^ tautomer of meropenem were generated using restrained electrostatic potential (RESP) fitting as implemented in the R.E.D. Server Development 2.0 ^41^ following geometry optimization at the HF/6-31G* level using Gaussian 16. Missing forcefield parameters were taken from analogous GAFF parameters. The carbamylated lysine (K56) in uncomplexed and acyl-enzyme structures was parameterized using this same approach. In both systems, all crystallographic waters were retained, and a disulfide bond was set by linking C27 and C45.

All simulations were prepared using the LEaP module in AmberTools18.^42^ Proteins were solvated in a truncated octahedron with TIP3P water molecules^43^ having a 10 Å minimum distance between any protein atom and the octahedron edge. Chloride ions were added to attain a neutral net charge along with an additional 150 mM NaCl concentration. Systems were minimized then heated to 300 K at constant volume over 200 ps. Short equilibration dynamics totaling 2 ns were run at constant pressure with isotropic position scaling at 1 bar, controlled by the Berendsen barostat with a pressure relaxation time of 1 ps. The temperature was maintained using Langevin dynamics with the collision frequency set to 5 ps^–1^. Protein backbone atoms (N, Cα, C) and non-hydrogen atoms of K56 and meropenem were constrained by a harmonic potential with a force constant of 10 kcal/mol – Å^2^ which was gradually reduced over the minimization, heating, and equilibration steps. The Particle Mesh Ewald (PME) method was used to calculate long-range electrostatic interactions while the short-range electrostatic and van der Waals interactions were truncated at 10 Å. All bond lengths involving hydrogen were constrained using the SHAKE algorithm.^44^ Unrestrained molecular dynamics simulations were performed for 500 ns with a timestep of 2 fs and coordinates were saved every 10 ps. All simulations were repeated five-fold (R1-R5) and run on the University of Bristol supercomputer BlueCrystal 4. The pmemd.cuda module of AMBER18 was used to run the simulations using the ff14SB forcefield^45^ at 300 K under periodic boundary conditions.

All simulations were analyzed using the *cpptraj* module within AmberTools18 having removed the first 75 ns of each trajectory to account for equilibration, unless otherwise stated. Mass-weighted root-mean-squared deviation (RMSD) calculations were computed with reference to the crystal structure. Average structures were calculated over 425 ns using N, Cα, and C atoms, and used as reference in root-mean-squared fluctuation (RMSF) calculations. Representative structures in Figures 4–6 are those having the lowest RMSD to the average structure calculated over all repeat simulations. For hydrogen bonding analyses the acceptor to donor heavy atom distance cutoff was set to 3.5 Å. Trajectories were visualized and manipulated using VMD.^46^ UCSF Chimera,^47^ , gnuplot and MATLAB were used to produce figures. The *mindist* function of AMBER20 was used to generate Supplementary Figures S25 and S26.

To explore the stability of the putative deacylating water molecule, a snapshot from the equilibrium MD simulations (trajectory 4, 75 ns), in which a water molecule occupied the deacylating position, was chosen. This was minimized over 2000 steps with 600 steps of steepest descent. The system was then heated to 298 K (Langevin thermostat) over 50 ps in the NVT ensemble and equilibrated over 2 ns in the NPT ensemble at 298 K and 1 atm of pressure (Langevin thermostat and Berendsen barostat). 100 kcal mol^–1^ Å^–2^ Cα restraints were implemented for the first 1 ns, whilst the second 1 ns was unrestrained. To maintain the water molecule in the deacylating position, 100 kcal mol^–1^ Å^–2^ restraints were applied to keep it within 3.8 Å of the carbonyl carbon in heating and both equilibration steps. Five independent repeats of 10 ns unrestrained MD simulation were then run at 298 K and 1 atm of pressure (Langevin thermostat and Berendsen barostat). All steps were run using AMBER20.

## Results

OXA-57, lacking its predicted signal peptide, was produced in recombinant *E. coli* BL21 (DE3) using the pET-28 T7 vector and was readily purified to homogeneity using immobilized metal ion affinity and size-exclusion chromatography (Figure S2). The purified protein eluted from the size exclusion column as a single peak at a volume consistent with the predicted molecular mass of the monomeric protein (27.1 kDa), with no evidence of a second peak that would indicate the presence of a dimer, as observed for some other class-D β-lactamases including OXA-10 and OXA-48.^48–50^ While previous data indicated a propensity for OXA-57 to dimerize in solution, in that study the dimeric form represented only 10% of the total protein content;^21^ hence we propose the monomer, rather than the dimer, to be the favored form of OXA-57.

### Hydrolysis of β-Lactam Substrates by OXA-57

Class D (OXA) β-lactamases differ widely in activity toward different β-lactams, with some (e.g., OXA-20 ^51^) efficiently hydrolyzing only penicillin substrates, while the activity of others extends to 3rd generation cephalosporins (e.g., OXA-10 ^16^) and/or carbapenems (e.g., OXA-48 ^48^). OXA-57 was reported to turn over penicillins and 1st generation cephalosporins.^21^ However, the activity of OXA-57 against later-generation cephalosporins (e.g., ceftazidime) and carbapenems, and the effect of NaHCO_3_ supplementation (to ensure maximal carbamylation of the active site lysine 73) have not been previously reported. We therefore determined steady-state kinetic parameters for OXA-57-catalyzed hydrolysis of a wider range of β-lactam substrates in the presence of NaHCO_3_ (Table 1). *k*_*cat*_/*K*_M_ values for turnover of the aminopenicillin ampicillin and the 1st generation cephalosporin cephalothin were close to those previously reported (0.0075 and 0.0017 s^–1^ μM^–1^, respectively^21^). However, hydrolysis was not observed for the (oxyimino)­cephalosporin substrates; cefotaxime and ceftazidime, or the monobactam aztreonam. By contrast, we observed low levels of hydrolysis for the carbapenems imipenem and meropenem, but it was necessary to undertake the steady-state experiments over several hours to obtain kinetic parameters (Figure S3a). OXA-57-catalyzed turnover of both imipenem and meropenem was slow (*k*_cat_, 0.04 ± 0.005 and 0.01 ± 0.002 s^–1^), with *K*_M_ values (51 ± 12 and 21 ± 7 μM) ∼10-fold weaker than those typically obtained for other class D β-lactamases (Table S2). In comparison with other class D β-lactamases, these values are closest to those described for OXA-10, an enzyme with only weak activity toward carbapenems and that is not usually considered a CHDL,^52^ rather than for members of the main CHDL lineages e.g., OXA-23 and OXA-48 (Table S2). Substrate inhibition of OXA-57 by both carbapenems was observed at higher concentrations (Figure S3b).

**Table 1:** Steady-State Kinetic Parameters for Hydrolysis of Selected β-Lactams by Purified Recombinant OXA-57^a^

Substrate	Antibiotic class	*k*_cat_ (s^–1^)	*K*_M_ (μM)	*K*_*i*_ (μM)	*k*_*cat*_ */K*_*M*_ (s^–1^ μM^–1^)^c^
Ampicillin	Penicillin	0.77 ± 0.1	131 ± 32	-	0.006 ± 0.002
Aztreonam	Monobactam	NH^b^	NH	NH	NH
Cephalothin	1st generation cephalosporin	0.57 ± 0.1	238 ± 44	405 ± 97	0.002 ± 0.0006
Cefotaxime	3rd generation cephalosporin	NH	NH	NH	NH
Ceftazidime		NH	NH	NH	NH
Imipenem	Carbapenem	0.04 ± 0.005	51 ± 12	265 ± 79	0.0008 ± 0.0002
Meropenem		0.01 ± 0.002	21 ± 7	646 ± 355	0.0005 ± 0.00002
FC5	Fluorogenic substrate	0.18 ± 0.01	18 ± 2	-	0.01 ± 0.001

a Experiments were carried out in triplicate, values quoted are means ± standard error. OXA-57 concentration was 1 μM throughout, apart from for FC5 where 12.5 nM enzyme was used.

b NH: no hydrolysis detected.

c Reported values are derived from those associated with *k*_cat_ and *K*_M_.

### Inhibition of OXA-57

β-Lactamase inhibitors (BLIs) are used in combination with β-lactams to circumvent β-lactamase activity, so extending the clinically useful lifetime of β-lactams.^53^ Both β-lactam compounds (e.g., clavulanate and tazobactam) that act as mechanism-based inhibitors, and non-classical inhibitors lacking a β-lactam core (e.g., the diazabicyclooctane (DBO) avibactam and the cyclic boronate vaborbactam) are now in clinical use. While clavulanic acid is reported to inhibit OXA-57, (*K_i_* 3.4 μM),^21^ its susceptibility to the new inhibitor classes has not previously been reported. We measured half maximal inhibitory concentrations (IC_50_) for inhibition of OXA-57 by clavulanate, avibactam and vaborbactam, measuring residual activity after pre-incubation with inhibitor for 10, 30 and 60 minutes (Table 2). Direct competition assays were carried out in the presence of NaHCO_3_, using as a reporter substrate the fluorogenic cephalosporin FC5,^27^ which showed comparable kinetic parameters to other OXA-57 substrates (Table 1). Clavulanic acid was the most effective OXA-57 inhibitor tested, with IC_50_ values ranging from 9.4–30.3 μM observed, depending upon pre-incubation time, and comparable to those for other class-D β-lactamases such as OXA-10 or OXA-48 (Table S3). Avibactam has slightly higher IC_50_ values, from 13.2–58.8 μM (Table 1), and is a less efficient inhibitor of OXA-57 than of some other OXA enzymes, e.g., OXA-10, OXA-23 and OXA-48, for which IC_50_ values in the nM range are reported (Table S3). Vaborbactam only very weakly inhibited OXA-57, consistent with its reported activity towards other class-D β-lactamases^54^ (Table S3), with an IC_50_ unable to be determined from measurements at up to 4 mM inhibitor.

**Table 2:** pIC_50_ and IC_50_ Values for Inhibition of OXA-57 by Selected β-Lactams and β-Lactamase Inhibitors^a^

Time (min)	Inhibitor	pIC_50_	IC_50_ (μM)
10	Clavulanate	4.5 ± 0.03	30.3
	Avibactam	4.2 ± 0.03	58.8
	Vaborbactam	<2.4	-
	Aztreonam	<2.4	-
	Cefotaxime	<2.4	-
	Ceftazidime	<2.4	-
30	Clavulanate	4.9 ± 0.04	12.3
	Avibactam	4.7 ± 0.04	18.8
	Vaborbactam	<2.4	-
	Aztreonam	<2.4	-
	Cefotaxime	<2.4	-
	Ceftazidime	<2.4	-
60	Clavulanate	5.0 ± 0.04	9.4
	Avibactam	4.9 ± 0.04	13.2
	Vaborbactam	<2.4	-
	Aztreonam	<2.4	-
	Cefotaxime	<2.4	-
	Ceftazidime	<2.4	-

a Each experiment was repeated four times, data represent the mean ± S.E.

As well as being effective antibiotics, some β-lactams efficiently inhibit certain β-lactamases. As steady-state measurements (above) failed to detect OXA-57-catalyzed hydrolysis of the 3rd generation (oxyimino)­cephalosporins cefotaxime or ceftazidime, or the monobactam aztreonam, we investigated their ability to inhibit OXA-57 via formation of long-lived acyl-enzyme species. However, we could not determine IC_50_ values for any of these compounds from measurements at concentrations up to 4 mM (Table 2), indicating that these β-lactams do not form stable complexes at the OXA-57 active site.

### Crystal Structure of Uncomplexed OXA-57

While some biochemical characterization of OXA-57 has been reported, a crystal structure has not been available. Accordingly, purified OXA-57 was crystallized from two different conditions in an identical crystal form (space group *C*222_1_ with one molecule in the asymmetric unit) and the structure solved to resolutions of 1.8 and 2.0 Å, respectively (Table S4). The two structures are effectively identical (RMSD 0.31 Å as determined by SSM Superpose^55^), although in neither case could we model a continuous polypeptide chain, with residues 209–214 (comprising the β4−β5 loop adjacent to the active site) and 243–245; and 209–214 and 243–248; omitted from the final models derived from the 2.0 Å and 1.8 Å datasets, respectively, due to poorly defined electron density in these regions. The discussion below, and associated molecular simulations, are based upon the 2.0 Å resolution structure as the model is more complete, although the data are of slightly lower quality.

The final modeled structure consists of eight α-helices surrounding a six-stranded antiparallel β-sheet (Figure 1a) with the conserved SXXK motif containing the nucleophilic serine residue^50^ located at the N-terminus of the α1 helix (residues S53 and K56). Of the two further sequence motifs conserved in OXA β-lactamases the SXV motif, whose serine (OXA-57 S104) relays protons in the class-D β-lactamase reaction mechanism^14,21^ (Figure S1), sits on the loop connecting helices α3 and α4 and the KTG motif (OXA-57 residues 201 – 203) located on the β4 strand. The Ω-loop (OXA-57 residues 137–152), a flexible region important to catalytic activity and substrate selectivity in class A β-lactamases,^56^ is well defined by the experimental electron density and could be modeled in its entirety.

**Figure 1:**
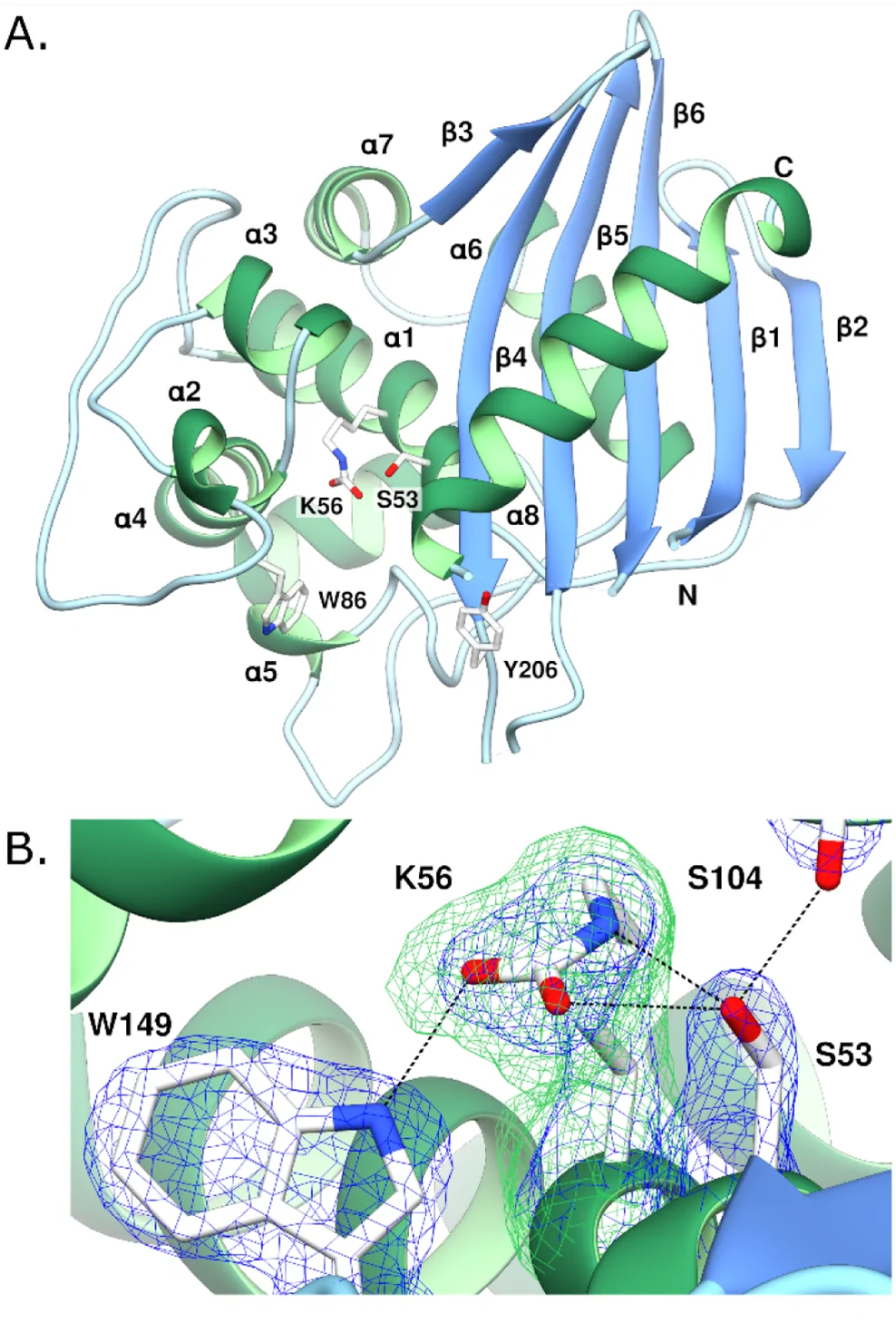
Crystal Structure View of OXA-57. A. Ribbon diagram of the overall structure, showing secondary structure elements and key residues. B. Active site showing 2*F*_o_ – *F*_c_ electron density (blue mesh, 1.5σ) for residues S53, K56, S104 and W149. Omit *F*_o_ – *F*_c_ map (green) calculated with K56 removed and contoured at 3σ shows carbamylation of K56. Hydrogen bonds are shown as dashed lines. The figure was generated using UCSF Chimera.^47^

Overall, the fold of OXA-57 is similar to those of other class-D β-lactamases (root-mean-square deviation values [RMSDs] for OXA-57 compared with selected OXA structures are presented in Table S5). Electron density was also well defined for mechanistically important residues in the OXA-57 active site; inspection of difference maps clearly showed that, consistent with previous studies,^14^ K56 is fully carbamylated (Figure 1b) in both uncomplexed structures, was refined with occupancy 1.0 and B-factors for the carbamate atoms equivalent to those of the K56 N_ε_ atom (Table S6)), and forms hydrogen bonds with S53 and with W149 on the Ω-loop. The overall structure closely (Cα rmsd 0.6 Å) resembles that of the search model used for molecular replacement (*B. thailandensis* YbxI, PDB 6NI0, 89.7% sequence identity, Table S5). Of the 21 positions in the crystallized OXA-57 sequence that differ in YbxI, only residues 141 (OXA-57 Q, YbxI K) in the Ω-loop, 148 (A/S) part of motif-5 ^35^ at the end of the Ω-loop, 204 (V/A) adjacent to the KTG motif (motif-6), and 205 (S/A), 218 (R/H) and 220 (S/F) in the β4−β5 loop (also referred to as the B-loop^35^) are located in regions of potential importance in ligand binding/hydrolysis (see below and Figure S4). Of these only OXA-57 V204 sits within the active site, but given that the backbone amide (part of the catalytically important oxyanion hole) rather than the side chain is involved in ligand interactions we consider it unlikely that substitution for A will substantially differentiate the (so far undisclosed) catalytic activity of YbxI from that of OXA-57.

### Structural Basis of OXA-57 Inhibition by Avibactam

To assess the effect of inhibitor exposure on the structure of OXA-57, crystals were soaked with avibactam for 16 hours. Diffraction data at 2.48 Å resolution (Table S4) showed clear *F*_o_ – *F*_c_ density in the active site that could confidently be modeled as ring-opened avibactam covalently attached to S53 (real-space correlation coefficient (RSCC) 0.96, Figure 2a, b). Soaking with avibactam did not alter the space group (*C*222_1_) or unit cell dimensions in comparison to those for the uncomplexed enzyme (Table S4) and avibactam induces no substantial changes to the overall structure (C_α_ RMSD 0.31 Å between the uncomplexed and avibactam-bound structures). Avibactam was refined at full occupancy with B-factors comparable to those for protein atoms (Table S4).

**Figure 2:**
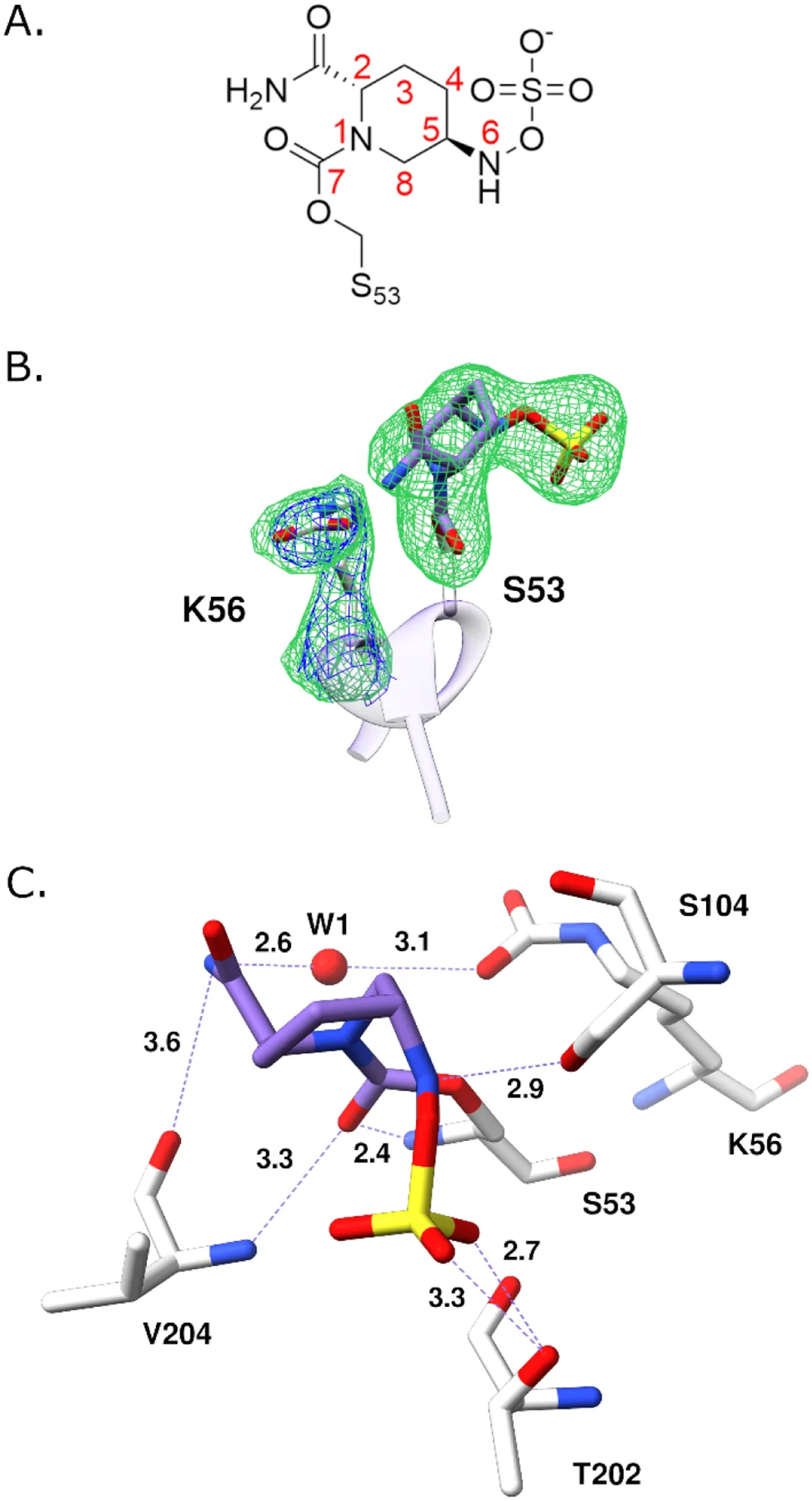
OXA-57:Avibactam Complex. A. Schematic of avibactam:enzyme complex showing avibactam atom numbering. B. Electron density of avibactam bound to OXA-57 (PDB 9HPY). 2*F*_o_ – *F*_c_ electron density (blue, 1.5σ) shows carbamylation of K56. Green density shows Omit *F*_o_ – *F*_c_ map (3σ) calculated with avibactam and carbamylated K56 removed. C. Interactions of avibactam with the OXA-57 active site. Avibactam carbon atoms are colored purple, non-carbon atoms by atom type, interacting residues are shown as white sticks, and water molecules as red spheres. Hydrogen bonding interactions are shown as dashed lines with distances between heavy atoms (Å) shown. This figure was generated using ChemDraw and UCSF Chimera.^47^

The opened six-membered piperidine ring of avibactam adopts a chair-like conformation, as seen in other SBL:avibactam complexes,^57,58^ with binding stabilized by an extensive network of hydrogen bonds (Figure 2c). The avibactam C7 carbonyl oxygen interacts with the backbone amides of S53 (2.4 Å) and V204 (3.3 Å) on the neighboring β4 strand. The avibactam-derived C2 carboxamide hydrogen bonds with the backbone carbonyl of V204 (3.6 Å). The avibactam-derived sulfate is stabilized by interactions between two of its oxygen atoms and the side chain hydroxyl of T202 (2.7 Å and 3.3 Å, respectively). In contrast to some complexes with other SBLs,^59–61^ our structure provides no evidence for desulfation of bound avibactam. K56 remains fully carbamylated (Figure 2b) as evidenced by B-factors for the carbamate group (Table S6); the carbamate is reoriented compared to its position in the uncomplexed structure (Figure S5), enabling hydrogen bonding to the avibactam C2 carboxamide via a water molecule (Figure 2c W1, 2.6 and 3.1 Å). The urea-derived N6 nitrogen of avibactam is oriented toward the carbonyl group of the acyl-enzyme-type complex, that is in position for an apparently stereo-electronically favored reaction to reform avibactam, consistent with the reversible nature of avibactam inhibition.^62^ In prior avibactam-derived structures, the N6 nitrogen of avibactam and related diazabicyclooctanes has been observed both in this conformation and one which is orientated away from the carbonyl group of the acyl-enzyme type complex.^63,64^

### Interactions of OXA-57 with Hydrolyzed Meropenem

To investigate the structural basis of OXA-57-catalyzed β-lactam (specifically carbapenem) hydrolysis, OXA-57 crystals were soaked with meropenem for 1 hr before cryo-cooling for subsequent diffraction experiments. Diffraction data extending to 2.42 Å resolution (Table S4) revealed positive *F*_o_ – *F*_c_ density in the active site consistent with ring-opened meropenem covalently linked to S53. Meropenem exposure did not affect the space group (*C*222_1_) or unit cell dimensions compared to those for the native enzyme (Table S4) and induced minimal changes to the overall structure, (C_α_ RMSD 0.38 Å).

Electron density was well defined for the meropenem-derived pyrroline ring, carbonyl oxygen, C2 exocyclic sulfur and C6 hydroxyethyl group (Figure 3b), but was weak for the remainder of the C2 substituent and C-3 carboxylate. However, at this resolution the orientation of the 6-α-hydroxyethyl group could not be unambiguously assigned, this was therefore modeled in an orientation pointing away from the carbamylated K56, with a C24-C6-C8-O22 dihedral angle of 161° (orientation II as defined by Chudyk *et al.* ^65^ and Hirvonen *et al.* ^66^), similar to that observed in carbapenem complexes of other class D β-lactamases.^63,67–69^ (Of note, inspection of difference maps, including those obtained after trial refinements with the 6-α-hydroxyethyl group in an inward-pointing orientation, did not indicate existence of any-subpopulations in this configuration, supporting our approach). The modeled ligand has an RSCC of 0.9 as defined in Phenix. Carbapenem hydrolysis by serine β-lactamases can generate alternative products; tautomerization of the Δ2-enamine product (in which the carbapenem C2 is *sp* ^*2*^ hybridized) results in the Δ1-imine in two equilibrating stereoisomeric forms (with C2 *sp* ^3^ hybridized^17^) (Figure S1). Based upon experimental electron density maps, which indicated the exocyclic C2 sulfur to be coplanar with the pyrroline ring, the meropenem-derived acyl-enzyme was modeled as the Δ2 enamine, though the presence of other tautomers is possible (Figure 3A, B).

**Figure 3:**
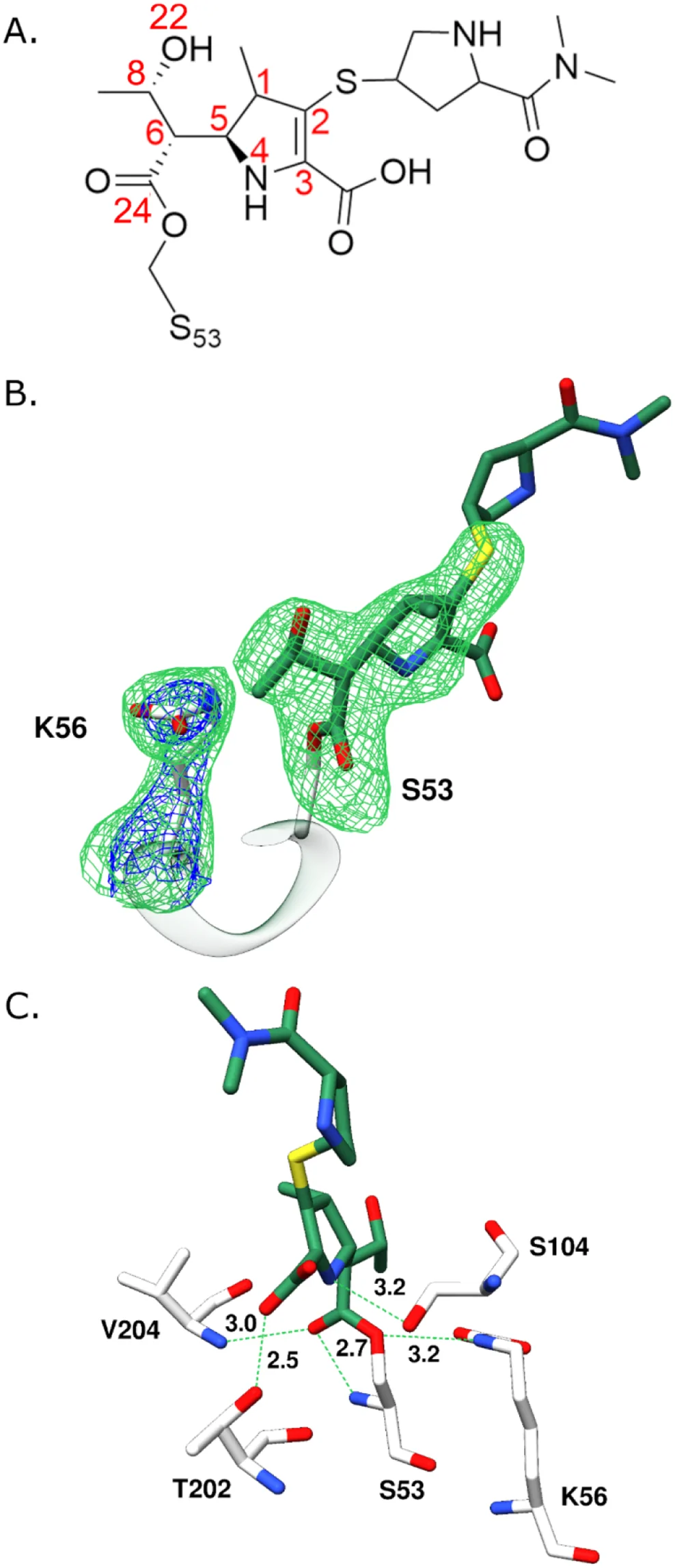
The Meropenem-derived Acyl-enzyme of OXA-57. A. Schematic of the meropenem acyl-enzyme in the Δ^2^ enamine tautomer. **B.** Electron density for meropenem bound in the active site of OXA-57 (PDB 9HPW). An Omit *F*_o_ – *F*_c_ map (green, 3σ) was calculated with meropenem and the carbamylated K56 removed. **C.** Key interactions of the OXA-57:meropenem acyl-enzyme. Meropenem carbon atoms are colored green, all other non-carbon atoms colored by atom type. Interacting residues are white sticks, and hydrogen bonding interactions are shown as dashed lines with distances between heavy atoms labeled in Å. This figure was generated using ChemDraw and UCSF Chimera.^47^

A network of hydrogen bonds stabilizes the meropenem derived acyl-enzyme (Figure 3C). The S53 oxygen atom interacts with the side chain amide of K56 (3.2 Å), which *F*_o_ – *F*_c_ electron density maps clearly reveal to be fully carbamylated and for which the carbamate was refined at full occupancy (Table S6). The acyl-enzyme carbonyl oxygen is positioned, *via* hydrogen bonds, in the oxyanion hole formed by the backbone amides of S53 and V204 (2.7 Å and 3.0 Å). The pyrroline nitrogen N4 hydrogen bonds to the S104 hydroxyl (3.2 Å) and the C3 carboxylate interacts with the side chain O of T202 (2.5 Å). However, no electron density was evident for an active site water molecule positioned for deacylation of the meropenem acyl-enzyme complex.

### Molecular Dynamics Simulations of Native OXA-57 and the OXA-57–Meropenem Acyl-Enzyme Complex

To investigate the dynamic behavior of OXA-57, and the effects upon it of acylation by meropenem, we ran five 500 ns molecular dynamics (MD) simulations (repeats 1–5, R1 - R5) of uncomplexed OXA-57 and the OXA-57:meropenem complex (totaling 2.5 μs per system). Our crystal structures were used as starting points for the simulations, with unstructured regions (corresponding to the β4−β5 and β6−α8 loops in native, uncomplexed OXA-57; and loops α1−α2, β4−β5 and β6−α8 in the OXA-57:meropenem acyl-enzyme complex) constructed as described in Methods (Figure S6). The time dependences of backbone RMSD values during each simulation is shown in Figures S7 and S8. In some cases, we observed variations in backbone RMSD over the course of the 500 ns simulations; these were subsequently recalculated excluding regions corresponding to the β4−β5 loop (residues S205–S220) and the C-terminus (residues L266–R269) resulting in RMSDs that stabilized around ∼1.0 Å (Figures S9, S10). These averaged 0.9, 1.0, 0.9, 1.0, and 1.3 Å for simulations R1–5 of native OXA-57; and 1.1, 1.0, 1.0, 1.1, and 1.0 Å for simulations of the OXA-57:meropenem complex. There were no marked differences in residue-based backbone RMSF distributions between the simulations of native OXA-57 and the meropenem acyl-enzyme (Figure S11). Based on these analyses the simulations do not provide evidence for slow, large-scale (global) conformational transitions taking place on this time scale (Figure 4).

**Figure 4:**
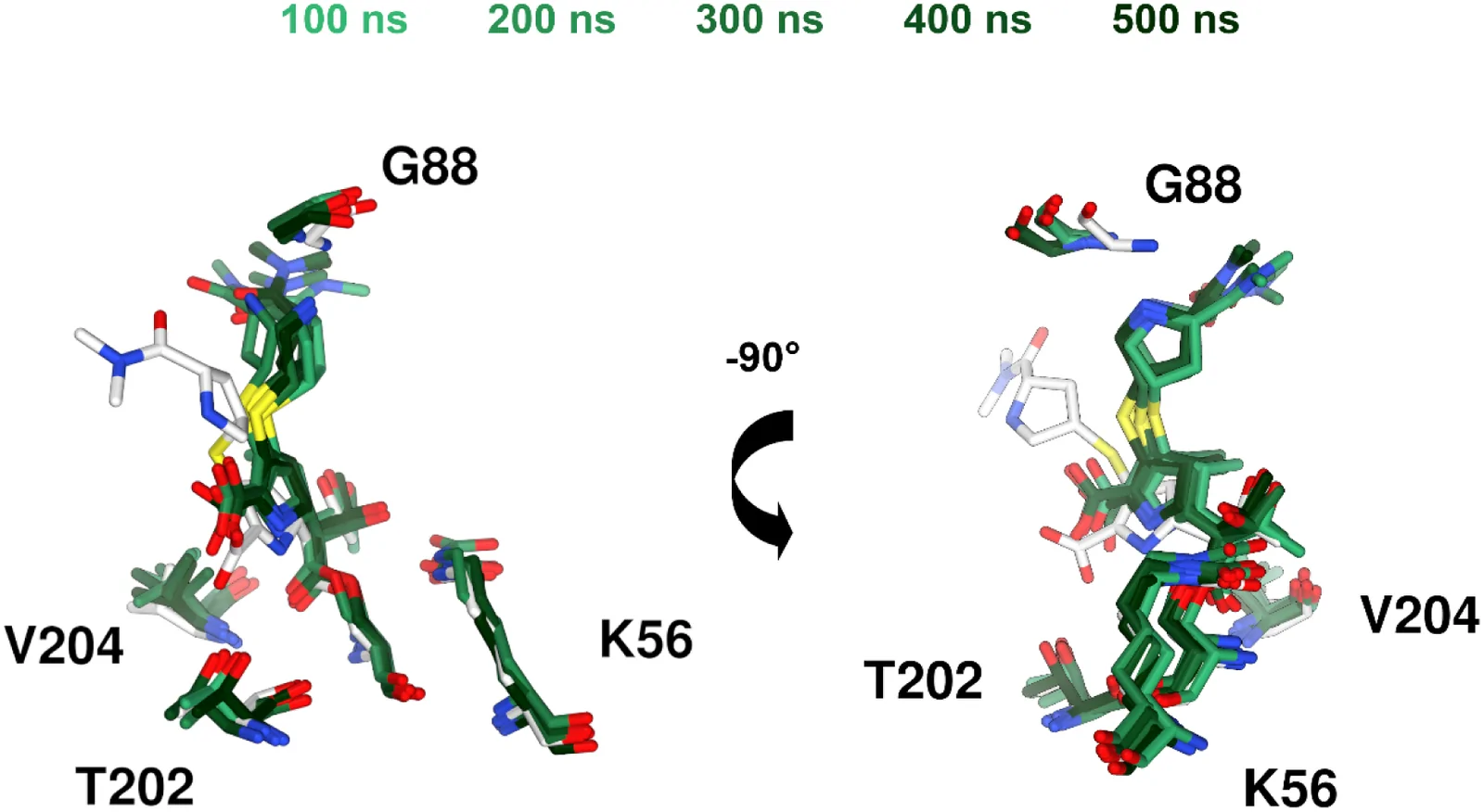
Relocation of Bound Meropenem in the OXA-57 Active Site. Superpositions of bound meropenem in snapshots taken at 100 ns intervals throughout the trajectory of MD simulations of the OXA-57:meropenem acyl-enzyme. Data shown are from simulation R5, with additional data provided in Supporting Information (Figure S17). Carbon atoms in the crystal structure are in white; carbon atoms from simulations are in green, snapshots at successive 100 ns intervals are shaded incrementally from light (100 ns) to dark (500 ns) green. Other atom colors as standard. This figure was generated using UCSF Chimera.^37^

In both cases, the β4−β5 loop is clearly the most mobile region of the protein. For the acyl-enzyme complex, RMSF values in the region corresponding to the β4−β5 loop were observed to increase in one replicate simulation (R3), although this was not evident in the other four simulations. Snapshots of structures taken at 100 ns intervals throughout the MD trajectories of uncomplexed OXA-57 and the meropenem acyl-enzyme indicate that the β4−β5 loop accesses similar conformations, and undergoes similar motions, regardless of the presence of meropenem (Figures S12, S13).

The carbamylated lysine side chain (residue K56) was stable over the course of the simulations of both native OXA-57 and the OXA-57:meropenem acyl-enzyme (average RMSDs 0.5 and 0.6 Å, respectively (Figures S14 and S15). However, in the meropenem acyl-enzyme, bound meropenem deviates from the crystallographically observed conformation within the first 100 ns of each simulation, with movements of the exocyclic sulfur and pyrrolidine ring of the C2 substituent largely accounting for the increase in RMSD. Bound meropenem adopts different conformations throughout the simulations, ultimately favoring a conformation approximately 5–6 Å distant from that observed in the crystal structure (Figure S16). During the MD simulations the C2 substituent (R-group) usually resides between the α1−α2 and β4−β5 loops (Figure S17), rather than pointing into bulk solvent.

Alterations in the interactions made by the meropenem C3 carboxylate group are associated with changes in the orientation of bound meropenem. The crystal structure of the OXA-57:meropenem acyl-enzyme suggests the presence of a hydrogen bond between the meropenem C3 carboxylate and the side chain hydroxyl of T202, although experimental electron density does not well define the orientation of the C3 carboxylate group. However, such an interaction is rarely evident during the MD simulations (Figure S18). Instead, the meropenem C3 carboxylate is occasionally hydrogen bonded to S104 (in on average approximately 4% of the trajectories via the carboxylate O01 atom, and 6% via O03) but, for most of the simulations, faces the bulk solvent and is coordinated by 1–6 (most commonly 3) water molecules (Figure S18). During the MD simulations we observe that the meropenem C3 carboxylate can form water-mediated interactions with the T202 hydroxyl group, and additionally with the hydroxyl group of T202 and atom H01 attached to the nitrogen of the meropenem pyrroline ring (Table S7). The high degree of mobility of, and absence of persistent interactions made by, the meropenem C3 carboxylate throughout the MD simulations is consistent with the poor electron density observed for this group in the crystal structure.

Hydrogen bonding analysis indicates that the meropenem-derived complex forms five hydrogen bonds to OXA-57 that are present in at least three of the five repeat simulations and over more than half of the MD trajectory (Figure 5). The meropenem carbonyl oxygen (O25) is positioned in the oxyanion hole formed by the backbone amides of V204 (average hydrogen bond occupancy 95% over the five MD simulations), and S53 (average occupancy 97%). The acyl-enzyme ester oxygen (OG) is hydrogen bonded to the side chain N01 of carbamylated K56 (72% occupancy). A hydrogen bond between N15 in the pyrrolidine ring of the meropenem C2 substituent and the backbone amide nitrogen of G88 located in the α1−α2 loop, is evident for over half of the trajectory in 4/5 of the repeat MD simulations (excepting R4). This interaction likely helps stabilize the extended conformation of the portion of the meropenem C2 substituent that lies beyond the exocyclic sulfur (Figure 5a).

**Figure 5:**
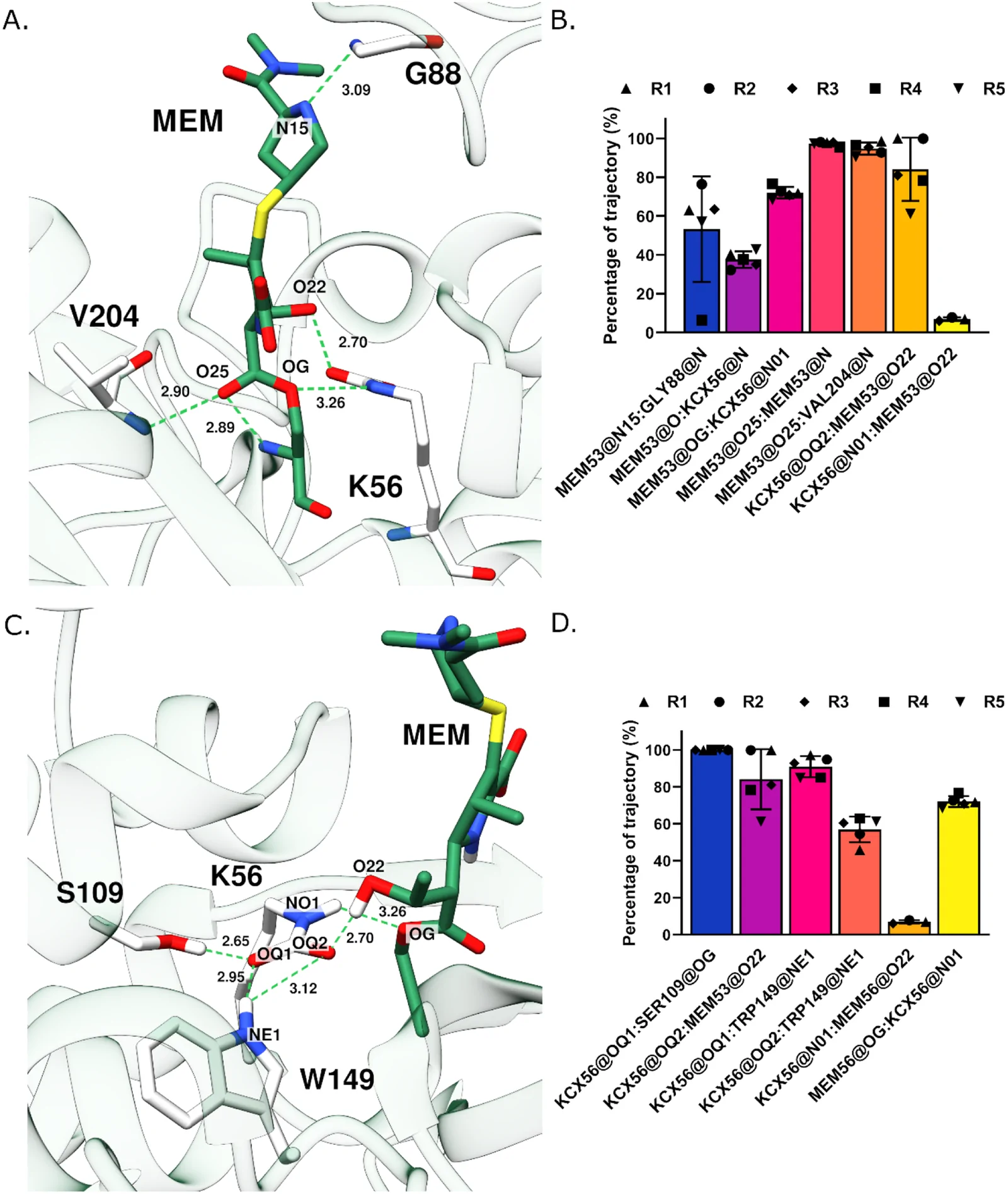
Hydrogen Bond Interactions Between the Meropenem Complex and OXA-57. A. Hydrogen bond interactions between OXA-57 and meropenem mapped onto a representative structure from simulations of the OXA-57:meropenem acyl-enzyme. **B.** Hydrogen bond interactions between OXA-57 and meropenem expressed as the mean percentage occupancy during 425 ns trajectories of simulations of the OXA-57:meropenem acyl-enzymes. **C.** Hydrogen bond interactions between carbamylated K56 and meropenem mapped onto a representative structure from simulations of the OXA-57:meropenem complex. **D.** Hydrogen bond interactions between carbamylated K56 and meropenem expressed as the mean percentage occupancy during 425 ns trajectories of the OXA-57:meropenem complex simulations. For **A** and **C**, the representative structure has the lowest RMSD to the average coordinates of all five repeat simulations of the OXA-57:meropenem acyl-enzyme. For clarity, only hydrogen bonds present for over half of the trajectories in at least three simulations are shown. Hydrogen bond distances shown are averages over all five simulations. For panels **B** and **D**, bar heights denote mean values, with error bars representing standard deviations. Values for individual repeats are shown as upward triangles (R1), circles (R2), diamonds (R3), squares (R4) and downward triangles (R5). Atoms involved in hydrogen bonds are specified as acceptor:donor. For clarity, only hydrogen bonds present in at least three simulations are shown. Meropenem (MEM) carbon atoms are shown in green, protein carbon atoms in white, other atom types atoms (including hydrogen atoms where involved in hydrogen bonds) in standard colors. Figure was generated using UCSF Chimera^47^ and GraphPad Prism.

Most notably, the meropenem C6 α-hydroxyethyl group is positioned to make persistent hydrogen bonds by sampling alternative positions from that adopted in the starting crystal structure (Figure S19). In the crystal structure, the C6 α-hydroxyethyl group was refined (as described above) in an “outward-facing” orientation (orientation II^65,66^ ) with the oxygen atom (O22) 5.18 Å away from the OQ2 oxygen of carbamylated Lys56, and a C24-C6-C8-O22 dihedral angle of 161° (Figure S19b). Over the course of the MD simulations, however, the C6 α-hydroxyethyl group repositions to spend the majority of the trajectories in “inward-facing” orientations, in which O22 is hydrogen bonded to Lys56 OQ2 (for 84% of the simulation time averaged across all 5 replicate simulations) and is consequently adopting “orientation III” with a C24-C6-C8-O22 dihedral angle approximating to 270 ° (Figure S19c). In addition, the R4 trajectory in particular occasionally samples “orientation I” (dihedral angle c. 50°) in which O22 is no longer hydrogen bonded to K56 OQ2 (Figure S19a), instead making it possible for an incoming water molecule to occupy the putative deacylating position (see below).

We also examined the hydrogen bonding network made by the carbamylated lysine K56 (Figure 5c). In addition to the hydrogen bond to meropenem O22 (i.e., the C6 α-hydroxyethyl group, present for 84% of the simulation time, above), the K56 carbamyl group makes a further two hydrogen bonding interactions: of OQ1 to the side chain hydroxyl of S109 (100% average occupancy), and of either OQ1 or OQ2 to the indole N atom NE1 of W149 (91/57%; Figure 5d). Atom N01 of the carbamylated K56 side chain makes an additional hydrogen bond to the meropenem C6 α-hydroxyethyl oxygen O22 (>60% occupancy in all five trajectories). Involvement in multiple high-occupancy hydrogen bonds ensures that carbamylated K56 retains a tightly defined conformation throughout the MD trajectories (Figures S14, S15).

Resolution of β-lactam acyl-enzymes of SBLs occurs through addition of water to the acyl-enzyme carbonyl, releasing the hydrolyzed product and regenerating active enzyme. However, our crystal structure of the OXA-57:meropenem acyl-enzyme did not contain any water molecules appropriately positioned for deacylation. Accordingly, the MD trajectories were investigated to search the OXA-57 active site for candidate deacylating water molecules. Water molecules within 10 Å of both the acyl-enzyme carbonyl carbon (meropenem C24) and OQ2 of carbamylated lysine K56 (the most likely general base for activation of an incoming water molecule, Figure 6A) were extracted from each frame of each of the five MD trajectories (42, 501 frames in total) and their positions and occupancies plotted (Figure 6B). In four of the five repeat simulations water molecules positioned for deacylation were either entirely absent (repeats R1 and R3) or present to a negligible degree (repeats R2 and R5, Figure S20). In simulation R4, some water molecules were observed located within 3.5 Å of both meropenem C24 and K56 OQ2, and thus favorably positioned for deacylation (Figure 6B), although in most frames water molecules, where present, are between 4–5 Å distant from meropenem C24 and 5–6 Å from K56 OQ2. In simulations R2, R4 and R5, water molecules reside within 3.5 Å of both meropenem C24 and K56 OQ2 in a total of 1, 602, and 32 frames, equating to 0.002, 1.4, and 0.08% of the simulations, respectively. Water molecules occupying this potentially deacylation-competent position can exchange with bulk solvent; in simulation R4, 3 individual water molecules are observed in this position, while 9 are observed in R5.

**Figure 6:**
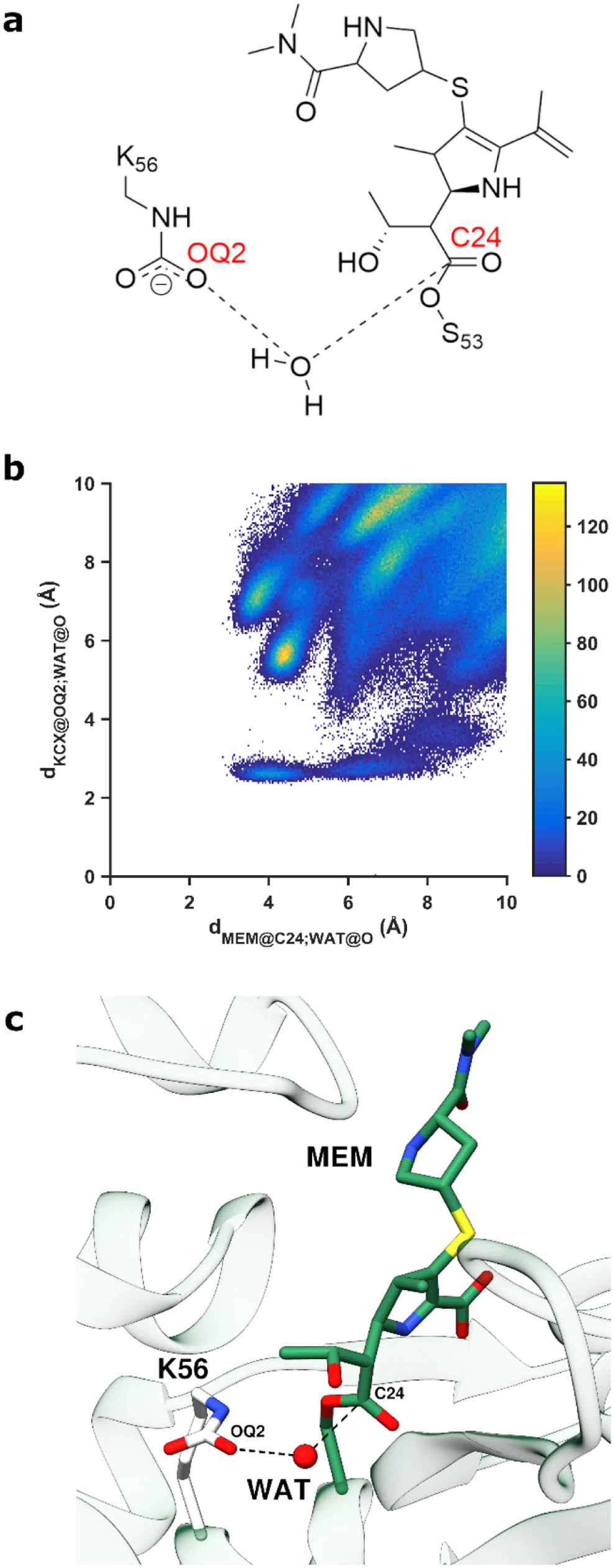
Candidate Deacylating Water Molecules are Transiently Present During MD Simulations of the OXA-57:meropenem Acyl-enzyme. A. Active site of the OXA-57:meropenem acyl-enzyme. Distance distributions calculated in **B** are shown as dashed lines. **B**. Distance distributions of water molecules between meropenem C24 (carbonyl carbon) and K56 OQ2 over 425 ns MD trajectories of the OXA-57:meropenem acyl-enzyme. Data shown are from simulation R4 (data for repeat simulations R1–3 and R5 can be found in Supporting Information (Figure S18)). **C**. Hydrogen bond interactions between K56, meropenem and water in the deacylating position mapped onto a representative structure from simulation R4 of the OXA-57:meropenem acyl-enzyme. Figure was generated using ChemDraw, MATLAB and UCSF Chimera.^47^

Water molecules positioned for possible deacylation also interact with the meropenem 6-α-hydroxyethyl group. Specifically, meropenem atom O22 (the hydroxyl of the C6α-hydroxyethyl substituent) lies within 3.5 Å of the candidate deacylating water in 601/602 and 25/32 of the frames where such a water molecule is present in repeat simulations R4 and R5, respectively (Figure 6c). These observations suggest that the hydrogen bond between meropenem O22 and K56 OQ2 must break for a water molecule to occupy the deacylating position, i.e., that the meropenem 6α-hydroxyethyl group rotates from orientation III (hydrogen bonded to K56 OQ2, as adopted across the majority of the MD simulations) to orientation I (hydrogen bonded to a putative deacylating water molecule, Figure S19). Indeed, in simulation R4, where candidate deacylating water molecules were most prevalent, meropenem O22 is not hydrogen bonded to K56 OQ2 when the water molecules are present. In simulations R2 and R5 there were very rare instances (one frame in R2, five in R5) when both a water molecule and the meropenem O22:K56 OQ2 hydrogen bond are observed.

To further investigate the stability of water molecules potentially positioned to support deacylation, we carried out additional MD simulations (5 × 10 ns) from a starting conformation (a snapshot from the R4 trajectory after 75 ns, Figure S19A) containing a water molecule in the putative deacylating position (Figure S21). Restraints were applied during the heating and equilibration steps; when these were removed the putative deacylating water molecule left its binding site (defined as an increase in distance to the meropenem acylenzyme carbonyl carbon atom (C24) and to OQ2 of carbamylated K56 to >5.0 Å, in 34% of the total frames (Figure S22). However, in only 8% of frames from these short simulations did the water molecule remain ≤3.5 Å from both meropenem C24 and K56 OQ2, i.e., well positioned to support deacylation. This behavior further supports our contention that water molecules positioned to deacylate the OXA-57:meropenem acylenzyme are unstable. Consistent with our kinetic analyses (above) showing carbapenems to be poor substrates for OXA-57, our MD simulations indicate that the active site of the OXA-57:meropenem acyl-enzyme does not readily stabilize a water molecule positioned for deacylation. The existence of a persistent hydrogen bond between meropenem O22 (of the C6α hydroxyethyl group) and a carboxylate (OQ2) oxygen of carbamylated K56 may be a contributing factor to this.

Two further factors have been identified as contributors to deacylation and carbapenem turnover in OXA β-lactamases. First, it has been suggested that the positioning and movement of a leucine residue (L166 in OXA-23) may play a critical role in permitting access of a water molecule to the active site to enable deacylation.^68^ In OXA-57 the analogous residue is an isoleucine (I150) located on the α5-α6 loop. Accordingly, we interrogated our simulations to look for possible correlation between motions of I150 and the presence/absence of a water molecule positioned for deacylation. In those MD simulations starting from the crystal structure, motions of the I150 side chain (plotted as side chain RMSDs, Figure S23) did not evidence any relationship to the presence of a water molecule in the deacylating position. For example, while the I150 side chain remains close (RMSD < 2.0 Å) to its starting position in replicate trajectory 1, in which no water molecule occupies the deacylating position (Figure S23A); in replicate 5, where I150 samples alternative conformations for the majority of the trajectory (Figure S23E), a putative deacylating water molecule is present in only 0.08% of frames. As a further test of this conclusion, we investigated the positioning of the I150 side chain in the set of shorter (10 ns) simulations starting from a conformation in which a putative deacylating water molecule was present. The results indicate that the I150 side chain samples a range of chi angles throughout these simulations, both in frames where a water molecule lies close (≤4 Å) to meropenem C24, and in those where it does not (Figure S24).

Secondly, in some CHDLs, a hydrophobic bridge flanking the active site is either present or forms during carbapenem hydrolysis,^70,71^ with the residues involved varying between OXA family members. Inspection of the OXA-57 structure identifies residues W86 and Y206, on the α1-α2 and β4-β5 loops, respectively (Figure 1A), as candidates to form a hydrophobic bridge across the active site cleft during the hydrolysis reaction, although they do not interact in either the uncomplexed or meropenem acyl-enzyme crystal structures. To investigate the possibility, and the potential mechanistic implications, of formation of such a bridge we calculated the minimum distance between any two carbon atoms in these residues during the 500 ns MD simulations of both the uncomplexed and meropenem acyl-enzyme structures. These analyses indicate that the two residues rarely approached within ≤4.0 Å of one another (Figures S25, S26). However, as close approaches are most prevalent in replicate trajectory 4, which is most associated with presence of a water molecule positioned for deacylation (above), we inspected snapshots along this trajectory to investigate whether closure of the active site might be associated with potentially active conformations. Such comparisons (Figure S27) indicate that the active site architecture, in particular the conformation of the β4−β5 loop, differs little between snapshots apparently compatible with deacylation (i.e., with a water molecule present in the deacylating water position and the meropenem 6α-hydroxyethyl group in orientation I); and those apparently not. In the latter case (e.g., at the end of the trajectory) no such water molecule is present and the meropenem 6α-hydroxyethyl group is in orientation II (as observed in the crystal structure). Hence our simulations do not support a role for I150 in maintaining an active site water molecule to deacylate bound meropenem, and do not identify formation of a hydrophobic bridge across the OXA-57 active site cleft as a feature that distinguishes potentially active conformations.

## Discussion

In agreement with previous work,^21^ our kinetic studies show OXA-57 to be a penicillinase that also hydrolyzes 1st generation cephalosporins (cephalothin) and which is inhibited by clavulanic acid at micromolar concentrations. Importantly, we show OXA-57 to be a weak carbapenemase, with kinetic parameters for carbapenems comparable to those previously reported for OXA-10 ^72^ (Table S2). Previous work indicates that class-D β-lactamases such as OXA-2 or OXA-10, with apparent narrow-spectrum and weak carbapenem-hydrolyzing activity, can elevate carbapenem MICs into the resistant range when expressed in impermeable producers, although the same effects were not evident in more permeable hosts such as laboratory *E. coli*.^72^ Thus, despite the weak activity of OXA-57 toward carbapenems *in vitro*, it is possible that its over-expression in an appropriate background could result in clinically relevant carbapenem MICs, as previously seen for the *B. pseudomallei* class A β-lactamase PenI.^9,73^ Significantly, efflux has also been reported as a contributing factor to carbapenem resistance in other Gram-negative pathogens.^74,75^ *B. pseudomallei* has 3 characterized resistance nodulation division (RND) efflux pumps which are utilized in resistance to other clinically important antibiotics.^76^ It is therefore possible that the weak carbapenemase activity of OXA-57, when present in a relatively impermeable host organism such as *B. pseudomallei*, and potentially in combination with upregulated efflux systems, may contribute to imipenem or meropenem resistance phenotypes in *B. pseudomallei* clinical isolates.

Of the tested compounds, clavulanic acid was the most effective β-lactamase inhibitor against OXA-57. In comparison with other class-D β-lactamases, avibactam was up to two orders of magnitude less active toward OXA-57, while OXA-57 showed very limited susceptibility toward vaborbactam, consistent with weak vaborbactam activity towards other class D enzymes (Table S3). Avibactam, like other DBO inhibitors, functions via a reversibly binding mechanism where a covalent inhibitory complex formed by carbamylation of the nucleophilic serine can undergo slow decarbamylation and recyclization that regenerates active inhibitor.^77^ For some SBLs, such as KPC-2 (class A) and AmpC (class C),^59–61^ avibactam and other DBOs can undergo desulfation leading to inhibitor degradation/hydrolysis and release of inactive products.^60,78^ Our crystal structure of the OXA-57:avibactam complex (obtained from crystals exposed to avibactam for 16 h before data collection), provides no evidence of desulfation, indicating that, as observed for other OXA enzymes,^57,60^ OXA-57 is likely to support reversible decarbamylation of DBOs through recyclization.

Avibactam binds the OXA-57 active site in a similar orientation to that observed in complexes with other class-D β-lactamases (OXA-10, OXA-24, OXA-48; Figure 7). In these four OXA:avibactam complex structures, one avibactam sulfate oxygen makes a hydrogen bond to a neighboring threonine (OXA-57 T202). In the OXA-57 complex this is the only hydrogen bond made by the avibactam sulfate, but in comparator structures the sulfate group makes further hydrogen bonds to a conserved arginine residue (Figure 7). OXA-57 contains an equivalent arginine (R255), but this is unfavorably oriented for interaction with the avibactam sulfate. Both OXA-10 and OXA-24 make additional interactions with avibactam that are absent from the OXA-57 complex; namely an additional hydrogen bond between the avibactam sulfate oxygens and the serine residue of the SXV motif (OXA-10 S115, OXA-24 S128; Figure 7a, b). In the OXA-24 complex, the avibactam sulfate further interacts with two active site water molecules (Figure 7b). The limited extent of interactions made with avibactam are consistent with its reduced potency toward OXA-57, compared to other class D enzymes.

**Figure 7:**
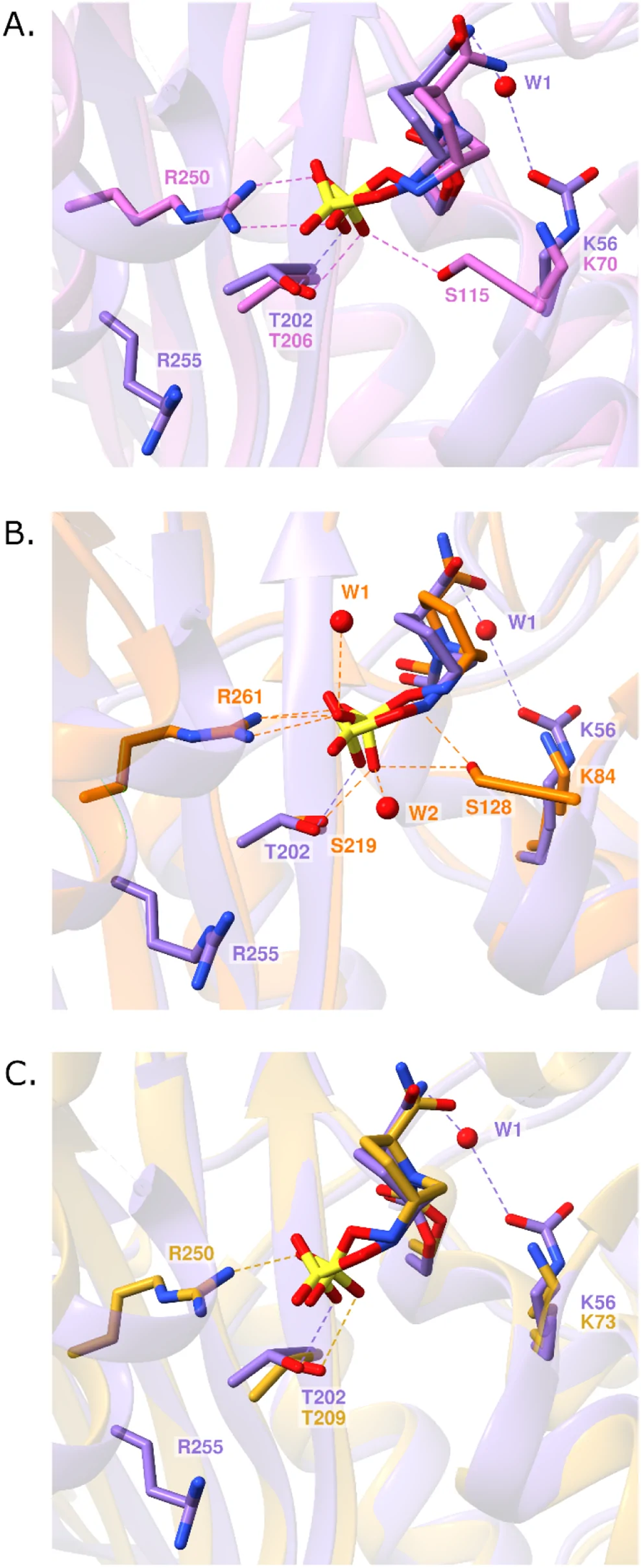
Avibactam Binding to Class D β-lactamases. **a**. Overlay of avibactam binding to OXA-57 (purple) and OXA-10:avibactam complex (PDB: 4S2O,^57^ pink). **b.** Overlay of avibactam binding to OXA-57 (purple) and OXA-24:avibactam complex (PDB: 4WM9,^58^ orange). **c.** Overlay of avibactam binding to OXA-57 (purple) with OXA-48:avibactam complex (PDB: 4WMC,^58^ gold). Non-carbon atoms are colored by atom type, hydrogen bonding interactions are shown as dashed lines. RMSD values between structures can be found in Supporting Information (Table S8). The figure was generated using UCSF Chimera.^47^

The most notable difference between the OXA:avibactam complexes is the presence of the carbamylated lysine in OXA-57. Carbamylation of the OXA active site lysine is pH-dependent, and favored under basic conditions,^14^ as utilized in crystallization of OXA-57 (OXA-10, pH 6.5; OXA-24, pH 6.0; OXA-48, pH 7.5 and OXA-57, pH 8.5). Although carbamylation of the active site lysine has been suggested to be disfavored in avibactam complexes of OXA β-lactamases,^57^ in the OXA-57:avibactam complex the carbamylated lysine makes a water-mediated interaction with the avibactam C2 carboxamide (Figure 7), potentially stabilizing both the lysine carbamate and bound avibactam. The role of lysine carbamylation in susceptibility to inhibition of OXA enzymes by avibactam (and other DBOs) however remains to be clarified.

In comparison to other CHDLs, *K*_M_ values for hydrolysis of the carbapenems imipenem and meropenem by OXA-57 are in most cases 5–10-fold higher, and *k*_*cat*_ values an order of magnitude lower (Table S2). Such differences may reflect factors including altered hydrogen bonding networks with bound substrates, and differences in active site accessibility to the water molecule required for deacylation. Superimposition of our OXA-57:meropenem structure on those of other OXA:carbapenem acyl-enzyme complexes identifies differing interactions with bound carbapenems (Figure 8). Hydrogen bonds in common with the OXA-1:doripenem^79^ and OXA-10:imipenem^84^ complexes (Figure 8a, b), which also contain a carbamylated active site lysine, include those between the carbapenem acyl-enzyme carbonyl oxygen and the oxyanion hole formed by the backbone amide nitrogens of the serine nucleophile and the residue immediately following the conserved KTG motif (OXA-57 V204, OXA-1 A215, OXA-23 W219, and OXA-48 Y211; Figure 8a, c, d). Similarly, a hydrogen bond between the carbapenem C3 carboxylate and the threonine residue of the active site KTG motif (OXA-57 T202) is present in all of these complexes. Interestingly, this hydrogen bond did not persist during MD simulations, which identified bound meropenem as relatively mobile within the OXA-57 active site. It is also important to note that, due to weak experimental electron density, we were unable to definitively assign the orientation of the carbapenem 6α-hydroxyethyl group in the OXA-57:meropenem acyl-enzyme complex crystal structure, and it was therefore modeled in an orientation (orientation II), close to the carbamylated K56, based on that observed in other OXA:carbapenem complexes.^68,69^

**Figure 8:**
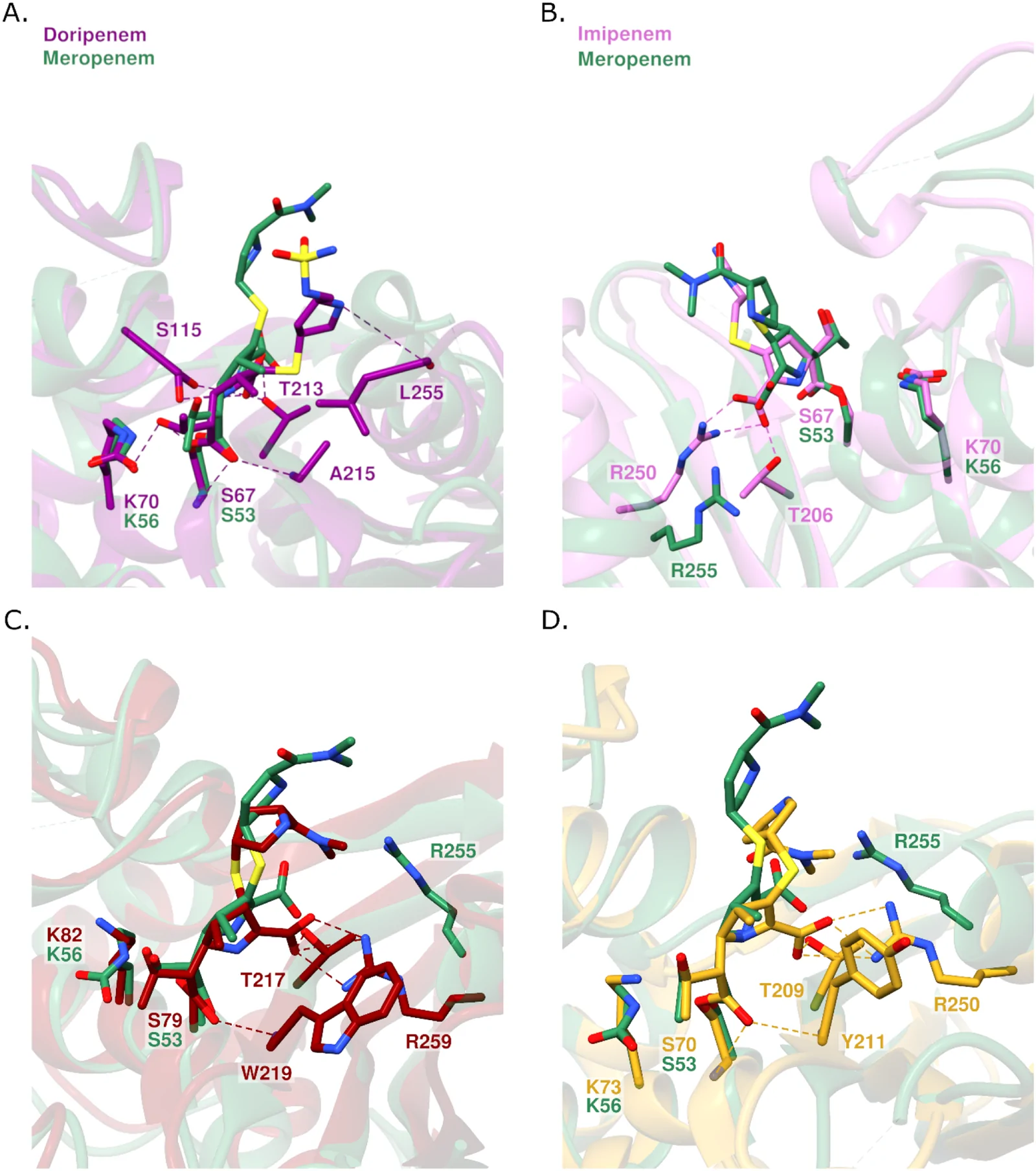
Carbapenem Binding to Class-D β-lactamases. **a.** Overlay of OXA-57:meropenem (green) and OXA-1:doripenem (PDB: 3ISG,^79^ purple) complexes. **b.** Overlay of OXA-57:meropenem (green) and OXA-10:imipenem (PDB: 6SKP,^84^ pink) complexes. **c.** Overlay of OXA-57:meropenem (green) and OXA-23:meropenem (PDB: 4JF4,^68^ red) complexes. **d.** Overlay of OXA-57:meropenem (green) and OXA-48:meropenem (PDB: 6P98,^69^ gold) complexes. For clarity, hydrogen bonds between OXA-57 and meropenem are not shown. Non-carbon atoms are colored by atom type and hydrogen bonding interactions are shown as dashed lines. RMSD values between structures are given in Supporting Information (Table S9). The figure was generated using UCSF Chimera.^47^

As is the case for the avibactam complexes described above, the most notable difference between the OXA-57:meropenem acyl-enzyme complex and comparator class D β-lactamase:carbapenem complexes is the observation that OXA-57 residue R255 is not favorably oriented to stabilize the meropenem acyl-enzyme. An arginine at the equivalent position is highly conserved in other CHDLs, and interacts with the C3 carboxylate of complexed carbapenems.^67–69^ The OXA-10:imipenem complex^84^ contains two such bonds (Figure 8b) and the OXA-23:meropenem^68^ and OXA-48:meropenem^69^ complexes three (Figure 8c, d). Intriguingly OXA-1, which is not classed as a CHDL and is considered to be inhibited by carbapenems,^79^ lacks an arginine at the equivalent position. However, this arginine residue does not appear to be essential for carbapenem hydrolysis, as the Gram-positive class-D β-lactamase BPU-1 also lacks arginine at the equivalent position but hydrolyzes carbapenems with comparable kinetic parameters to CHDLs from Gram-negative sources^80^ (Table S2).

Carbapenems are distinct from other β-lactams in the ability of their ring-opened species (acyl-enzymes or hydrolysis products) to access alternative tautomeric forms of their pyrroline ring: the Δ^2^ (enamine) and the Δ^1^ (imine, in both the (*S*)-Δ^1^ and (*R*)-Δ^1^ configurations).^18^ Previous studies with some ESBLs, such as SHV-1 and RTEM, have proposed the Δ^2^ enamine tautomer to be most relevant for efficient carbapenem hydrolysis,^81,82^ and that efficient carbapenemases either minimize the extent of Δ^1^ tautomer formation or facilitate tautomerization of the Δ^1^-imine back to the Δ^2^-enamine.^83^ In our OXA-57:meropenem structure, the meropenem acyl-enzyme was modeled as the Δ^2^-enamine, based upon the location of the exocyclic sulfur. Various tautomeric states are crystallographically observed in the OXA:carbapenem complexes compared in Figure 8. In both the OXA-10:imipenem^84^ and OXA:48:meropenem^69^ complexes, bound carbapenems are modeled as the Δ^2^-enamine tautomer. However, in the OXA-1:doripenem^79^ and OXA-23:meropenem^68^ structures, the carbapenem acyl-enzymes were modeled in the Δ^1^-imine tautomer in the (*R*)-Δ^1^ and (*S*)-Δ^1^ configurations, respectively.^68,79^ Considering these observations in the context of the reported kinetic parameters for carbapenem hydrolysis by each of these enzymes (Table S2), it is clear that catalytic efficiency toward carbapenems does not necessarily correlate with the presence of a specific acyl-enzyme tautomer, at least as observed in the relevant crystal structures. In the case of OXA-57 our assignment of predominantly bound meropenem in the Δ^2^-enamine tautomer, while consistent with the ability of the enzyme to hydrolyze meropenem, is not then an indicator of strong activity toward this substrate.

Access to the active site by the water molecule necessary for deacylation is fundamentally important to carbapenem hydrolysis by CHDLs.^15^ The carbamylated lysine is thought to act as a general base, activating the incoming water molecule to attack the acyl-enzyme carbonyl carbon, ultimately liberating the inactivated product (Figure S1).^14^ It has been proposed that steric hindrance during acylation, involving the carbapenem 6α-hydroxyethyl group, causes expulsion of water from the active site necessitating subsequent recruitment of water from bulk solvent.^68,85^ In the case of OXA-57, we found no evidence of solvent molecules positioned for deacylation in the active site of the uncomplexed enzyme, and there was no candidate deacylating water in the OXA-57:meropenem acyl-enzyme complex structure. While, given the comparatively low resolution of our crystallographic data, we cannot rule out the presence of a water molecule with insufficient occupancy to justify its inclusion in our final model, our MD simulations of the meropenem acyl-enzyme indicate only sporadic presence of an active site water molecule positioned for deacylation.

Different routes by which the deacylating water molecule can access the active site have been proposed for different CHDLs. In OXA-23, a hydrophobic patch formed by the conserved residues V128 and L166 shields the carbamylated lysine. Upon acylation, reorientation of the side chains of these residues causes formation of a channel close to the carbamylated lysine, allowing access of a water molecule.^68,85^ In contrast, uncomplexed OXA-48 possesses a channel between the hydrophobic patch (residues V120 and L158) and the serine nucleophile that provides direct access of water to the carbamylated lysine, with acylation leading to only minimal conformational changes that widen this channel to promote water access.^69^ In OXA-57 V106 and I150 form an equivalent hydrophobic patch, but the OXA-57:meropenem acyl-enzyme complex structure provides no evidence of conformational changes leading to formation of a water access channel as described for OXA-23; and neither the uncomplexed or meropenem-bound OXA-57 structures contain a preexisting solvent channel to the active site as reported for OXA-48.^69^

We interrogated our MD simulations to investigate whether OXA-57 I150 might act as a “gating” residue regulating access of water to the active site. In 4 of our 5 repeat MD simulations I150 sampled different conformations throughout the trajectory (Figure S23), but there was no correlation between specific conformations and presence of water in the deacylating position, and the average RMSF of the I150 side chain (1.0 Å) is consistent with the average RMSF values for all side chains in the acyl-enzyme complex. Furthermore, in simulations starting from a conformation that contained a putative deacylating water molecule, the Ile150 side chain sampled a range of chi angles regardless of the continued presence of this water (Figure S24). Hence our simulations do not support a role for I150 facilitating or regulating access of water to the OXA-57 active site to enable deacylation. Interestingly, the OXA-48 L158I mutant reduced catalytic efficiency toward meropenem, and also disfavored production of the alternative, β-lactone product as recently described for carbapenem hydrolysis by several class D enzymes.^18,86^ This observation indicates that the presence of I150 in OXA-57 may be a contributing factor to its weak carbapenemase activity.

A noteworthy feature of some CHDLs, in particular enzymes such as OXA-23 and OXA-24 that originate in *Acinetobacter* spp., is formation of a hydrophobic bridge spanning the active site cleft. As first identified in OXA-24 ^71^ the hydrophobic bridge involves contacts between the side chains of conserved residues flanking the active sight cleft, typically phenylalanine/tyrosine in the α3−α4 loop and methionine/tryptophan on the β5−β6 loop. In some cases, e.g., OXA-58, this bridge only forms upon ligand binding.^70^ Candidate residues for involvement in hydrophobic bridge formation in OXA-57 are W86, on the α1−α2 loop and Y206 on the β4−β5 loop. However, the uncomplexed OXA-57 structure provides no evidence for a hydrophobic bridge between W86 and Y206, which lie 12 Å distant from one another, and conformational changes leading to formation of a hydrophobic bridge are not observed in the crystallized OXA-57:meropenem complex. Moreover, approach of W86 and Y206 within ≤4.0 Å of one another was rarely observed in MD simulations (Figures S25, S26), such “closed” configurations were observed in both presence and absence of a putative deacylating water molecule (Figure S27), and these residues were generally further apart in simulations of the acyl-enzyme complex than in the uncomplexed enzyme. We conclude that, in contrast with the equivalent complexes of some other CHDLs, OXA-57 does not form a persistent hydrophobic bridge across the active site cleft of the carbapenem acyl-enzyme complex.

The hydrophobic bridge plays a pivotal role in carbapenem hydrolysis by many CHDLs through constraining the tautomeric configuration of the carbapenem acyl-enzyme.^87^ However, such a bridge is not present in all CHDLs, and carbapenem turnover occurs with comparable or even better efficiency in β-lactamases such as the OXA-48 group from which it is absent. In OXA-48, an alternative mechanism is proposed whereby a tyrosine (Y211) in an equivalent position to the methionine residue of the OXA-23 hydrophobic bridge orients toward the active site cleft forming a “pseudobridge” preventing formation of the (*R*)-Δ^1^ acyl-enzyme tautomer.^69^ OXA-57 Y206 occupies the equivalent position to OXA-48 Y211, but is in a different orientation that is not consistent with a role in pseudobridge formation, and as described above makes only very transient interactions across the active site cleft during MD simulations. Thus, none of our findings support a role for OXA-57 Y206 in constraining carbapenem tautomerization, and our data do not identify any alternative mechanism by which this is achieved.

To investigate the broader implications of our observations, we carried out a genomic analysis of 1,286 *B. pseudomallei* clinical strains from Northeast Thailand^88^ and identified several chromosomal OXA isoforms, including OXA-59, along with other previously uncharacterized variants. These isoforms differ from OXA-57 by up to 4 single amino acid polymorphisms (SAPs). When the locations of these polymorphisms were mapped onto the OXA-57 crystal structure, most were not found in positions directly interacting with bound β-lactam substrates, and are thus unlikely to impact specificity or turnover (Figure S28). Indeed, previous kinetic studies identified minimal effects of specific substitutions, such as K231N in OXA-42 and D170N in OXA-59, on catalytic activity or substrate specificity.^21^ Two exceptions were identified: the S104P substitution in OXA-42 (not found in our strain collection) abolishes β-lactam hydrolysis *in vitro*, and the K56R substitution in a variant type (VT8), observed in a single strain, could have functional implications. In summary, therefore, we expect the properties of OXA-57 described here to be representative of chromosomal OXA-β-lactamases from *B. pseudomallei*.

## Conclusion

The data herein reveal *B. pseudomallei* OXA-57 to be a class-D β-lactamase capable of slow turnover of carbapenem as well as penicillin substrates, incapable of hydrolyzing (oxyimino) cephalosporins, and inhibited by clavulanate and (more weakly) avibactam. OXA-57 is monomeric (rather than dimeric as is the case for many other OXA enzymes) with an overall fold similar to those of other OXA enzymes, but with an extended, unstructured β4-β5 loop adjacent to the active site. Structures of complexes with avibactam and the acyl-enzyme complex of the carbapenem meropenem do not reveal substantial conformational changes associated with ligand binding, while a conserved active site arginine (OXA-57 R255) involved in ligand interactions of other OXA enzymes does not participate in hydrogen bonds to either the avibactam sulfate or meropenem C3 carboxylate groups. This observation is consistent with the relatively weak affinity of OXA-57 for both avibactam and meropenem. Molecular dynamics simulations of uncomplexed OXA-57 and its meropenem acyl-enzyme (assigned as the Δ^2^ tautomer) show reorientation of bound meropenem away from the position defined by the crystal structure, low occurrence of an active site water molecule positioned for deacylation and a lack of conformational changes, such as loop closure or hydrophobic bridge formation across the active site, associated with carbapenem hydrolysis by other class D β-lactamases. Thus, although kinetic data identify weak carbapenem-hydrolyzing activity by OXA-57, and the crystal structure indicates meropenem to be present in the (more hydrolytically labile) Δ^2^ -enamine tautomer, we do not identify specific features of OXA-57 that are associated with carbapenemase activity. This is consistent with suggestions that weak carbapenemase activity is an inherent property of class D β-lactamases.^72^ However, despite the weak *in vitro* activity of OXA-57, its coexistence with other resistance mechanisms, such as efflux pumps and alterations to permeability, and the potential for enhanced activity in the host cell through upregulation of expression and/or acquisition of point mutations (as has occurred in e.g., *Acinetobacter* sp.^89^), suggest that OXA-57 and its variants should be considered as a potential contributing factor to β-lactam, in particular carbapenem, resistance in *B. pseudomallei*. Genomic analyses of chromosomal OXA genes in clinically derived strains of *B. pseudomallei* suggest our observations with OXA-57 are likely to be of general relevance. The unusual properties of OXA-57, as revealed by our combined biochemical and biophysical studies, also identify scope for improvement in β-lactamase inhibitors with potential application as part of new combination therapies for treating melioidosis.

## Supplementary Material



## Data Availability

Starting models, input files, and representative snapshot structures from MD simulations will be made freely available at the University of Bristol Research Data Repository (https://data.bris.ac.uk/).

## Notes

Tables showing: PCR primers used for construct generation; comparison of carbapenem hydrolysis kinetics for class D β-lactamases; IC50 values for clavulanate, avibactam, and vaborbactam inhibition of selected class D β-lactamases; x-ray data collection and refinement statistics; sequence and structural similarity of OXA-57 to other class D β-lactamases; B-factors for Lys56 carbamate; interactions of a water molecule bridging meropenem and OXA-5 T202 in MD simulations; comparisons of selected OXA:avibactam and OXA:carbapenem complex structures; figures showing: proposed OXA-57 hydrolysis mechanism; purification of OXA-57; carbapenem hydrolysis by OXA-57; positions in the OXA-57 structure of residues differing between OXA-57 and *B. thailandensis* YbxI; alternative conformations of carbamylated K56 in the OXA-57:avibactam complex structure; starting models for molecular dynamics (MD) simulations; time series of backbone, core, K56, and meropenem RMSD values for MD simulations of uncomplexed OXA-57 and meropenem acyl-enzyme; backbone RMSF values for MD simulations of uncomplexed OXA-57 and the OXA-57:meropenem complex; snapshots of structures of the protein and bound meropenem during MD simulations of uncomplexed OXA-57 and OXA-57:meropenem; time series of hydrogen bond interactions made by meropenem during MD simulations of the OXA-57 complex; alternative orientations of the meropenem 6α-hydroxyethyl group; distribution of active-site water molecules in MD simulations of the OXA-57:meropenem complex; stability of MD simulations starting from a conformation with a putative deacylating water; active-site water distributions during MD simulations; mobility of residue I150 during MD simulations of the OXA-57:meropenem complex; mobility of active-site loops in MD simulations of uncomplexed OXA-57 and the OXA-57:meropenem complex; orientations of active-site loops; sequence polymorphisms in *B. pseudomallei* chromosomal OXA β-lactamases (**PDF**)

## ¶

Diamond Light Source, Harwell Science and 975 Innovation Campus, Didcot OX11 0DE, U.K

## ††

Department of Life Sciences, University of Bath, 987 Bath, BA2 7AY, U.K.

## ‡‡

Department of Medical Science, Amnat Charoen Campus, 989 Mahidol University, Amnat Charoen, Thailand.

## Abbreviation

ESBL: Extended-spectrum β-lactamases
IMAC: Immobilized metal affinity chromatography
NH: No hydrolysis
BLI: β-Lactamase inhibitor
DBO: Diazabicyclooctane
IC_50_: Half-maximal inhibitory concentration
RMSD: Root-mean-square deviation
RSCC: Real-space correlation coefficient
RMSF: Root-mean-square fluctuation
CHDL: Carbapenem-hydrolyzing class D β-lactamases
MIC: Minimum inhibitory concentration
