# Supplementary material for: Structure and Dynamics of Burkholderia pseudomallei OXA-57, a Distinctive Low-Efficiency Class D β‑Lactamase with Carbapenem-Hydrolyzing Activity
Source: Biochemistry. 2026 Oct 22;65(21):3514–32. doi: 10.1021/acs.biochem.6c00668 (PMC13738345; doi:10.1021/acs.biochem.6c00668)
Supplement: Supplementary file 1 [file bi6c00668_si_001.pdf]

Structure and Dynamics of *Burkholderia pseudomallei* OXA-57, a Distinctive Low-Efficiency Class D β-Lactamase with Carbapenem-Hydrolyzing Activity

Supporting Information

Éilís C. Bragginton [1,2†], Charlotte K. Colenso [1,3], Michael Beer [1,3], Karina Calvopiña [4], Philip Hinchliffe [1], John M. Shaw [1], Catherine. L. Tooke [1,5#], Rathanin Seng [6,7‡], Narisara Chantratita [6,8,9], Adrian J. Mulholland [3], Christopher J. Schofield [4] and James Spencer [1]*

[1] School of Biochemistry and Biomedical Sciences, University of Bristol, Bristol, BS8 1TD, U.K.

[2] Diamond Light Source, Harwell Science and Innovation Campus, Didcot OX11 0DE, U.K.

[3] Centre for Computational Chemistry, School of Chemistry, University of Bristol, Bristol, BS8 1TS, U.K.

[4] Chemistry Research Laboratory, Department of Chemistry and the Ineos Oxford Institute for Antimicrobial Research, University of Oxford, Oxford, OX1 3TA, U.K.

[5] Department of Life Sciences, University of Bath, Bath, BA2 7AY, U.K.

[6] Department of Microbiology and Immunology, Faculty of Tropical Medicine, Mahidol University, Bangkok 10400, Thailand.

[7] Department of Medical Science, Amnat Charoen Campus, Mahidol University, Amnat Charoen 37000, Thailand.

[8] Mahidol-Oxford Tropical Medicine Research Unit, Faculty of Tropical Medicine, Mahidol University, Bangkok 10400, Thailand.

[9] The Academy of Science, The Royal Society of Thailand, Bangkok 10300, Thailand

*Corresponding author: Jim.Spencer@bristol.ac.uk

Table S1. Primers used to Generate Recombinant Vector pET28b (+) *OXA-57* (Δ1-69).

Primers*	Sequence
Forward	CGCGCGGCAGCCATATGAAGACTATCTGCACGGCTATTGCG
Reverse	GCTCGAATTCGGATCCTTAGCGGGCTGCGAGTAAGCG

*Primers were supplied by Eurofins Genomics.

Table S2. Comparison of Class-D β-Lactamase Steady-State Kinetics for Imipenem (IMI) and Meropenem (MER) Hydrolysis.*(1-3)*

Class-D β-lactamase	Organism	K_M (μM)		k_{cat} (s^{-1})		k_{cat}/K_M (M^{-1} s^{-1})	
		IMI	MER	IMI	MER	IMI	MER
OXA-57	*B. pseudomallei*	51.0	21.0	0.04	0.01	7.8 x 10^2	3.3 x 10^3
OXA-2[1]	*P. aeruginosa*	≤2.0	≤2.0	0.18	0.11	≥9.0 x 10^4	≥5.5 x 10^4
OXA-10[1]	*P. aeruginosa*	2.0	5.6	0.039	0.041	2.1 x 10^4	7.0 x 10^3
OXA-23[2]	*A. baumanii*	4.8	≤1.0	0.35	0.068	7.4 x 10^5	≥6.8 x 10^4
OXA-24[1]	*A. baumanii*	≤2.0	≤2.0	1.7	0.11	≥8.5 x 10^5	≥5.5 x 10^4
OXA-48[1]	*K. pneumoniae*	5.3	≤2.0	4.8	0.16	1.3 x 10^6	≥8.0 x 10^4
OXA-58[1]	*A. baumanii*	6.0	≤2.0	1.8	0.019	2.9 x 10^5	≥9.5 x 10^3
BPU-1[3]	*B. pumilus*	6.6	4.2	0.26	0.014	6.2 x 10^3	3.3 x 10^3

Table S3. Comparison of IC_{50} Values for Clavulanic acid, Avibactam and Vaborbactam Inhibition of Selected Class-D β-lactamases*(4-8)*.

Class-D β-lactamase	Organism	Clavulanate		Avibactam		Vaborbactam	
		Incubation time (min)	IC_{50} (μM)	Incubation time (min)	IC_{50} (μM)	Incubation time (min)	IC_{50} (μM)
OXA-57	*B. pseudomallei*	10<br>30	30.3<br>12.3	10<br>30	58.8<br>18.8	-	
OXA-10	*P. aeruginosa*	NA	*< 40[4]	10<br>30	0.17[6]<br>0.04[6]	NA	> 400[7]
OXA-23	*A. baumanii*	-		10<br>30	0.09[6]<br>0.17[6]	10	* 120[8]
OXA-48	*K. pneumoniae*	30	*16[5]	10<br>30	0.50[6]<br>0.14[6]	NA	32[7]

*Reaction not supplemented with $NaHCO_3$

NA: not available.

Table S4. X-ray Diffraction Data Collection and Refinement Statistics.

	Uncomplexed OXA-57<br><br>PDB: 9HPT	Uncomplexed OXA-57<br><br>PDB: 9HPU	OXA-57: avibactam<br><br>PDB: 9HPY	OXA-57: meropenem<br><br>PDB: 9HPW
**Data Collection**				
Space Group	C 2 2 21	C 2 2 21	C 2 2 21	C 2 2 21
**Cell dimensions**				
a, b, c (Å)	66.22, 111.56, 66.20	65.79, 110.35, 64.80	65.78, 110.84, 66.16	65.93, 109.86, 66.36
α, β, γ (º)	90, 90 ,90	90, 90, 90	90, 90 ,90	90, 90 ,90
Wavelength (Å)	0.9	0.98	0.91	0.91
Resolution (Å)	43.17 – 2.0 (2.05 – 2.0)	42.59 – 1.80 (1.84 – 1.80)	55.42 – 2.48 (2.58 – 2.48)	56.53 – 2.42 (2.51 – 2.42)
R_{pim}	0.020 (1.041)	0.049 (0.917)	0.048 (0.732)	0.036 (0.568)
CC1/2	1.0 (0.532)	0.998 (0.38)	0.999 (0.59)	0.997 (0.667)
$I/\sigma(I)$	17.3 (0.7)	8.9 (0.8)	9.0 (0.8)	11.7 (1.2)
Completeness (%)	100.0 (100.0)	100.0 (100.0)	100.0 (100.0)	99.7 (99.3)
Redundancy	13.1 (12.0)	11.4 (12.7)	12.3 (12.9)	12.8 (12.8)
**Refinement**<br>Resolution (Å)	43.17 – 2.0	42.01 – 1.80	55.42 – 2.48	56.53 – 2.42
No. Reflections	16959	22237	8825	9432
R_{work}/R_{free}	0.2072/0.2311	0.1890/0.2143	0.1925/0.2451	0.1971/0.2601
**No. non-H atoms**				
Protein	1831	1810	1812	1800
Solvent	24	134	9	12
Ligand	-	1	17	26
***B*-factors (Å²)**				
Protein	65.6	36.2	76.4	79.9
Solvent	53.8	39.6	74.3	71.2
Ligand	**-**	42.0	71.1	114.6
**r.m.s deviations**				
Bond lengths (Å)	0.007	0.006	0.008	0.007
Bond angles (º)	0.832	0.830	1.01	0.88
**Ramachandran (%)**				
Outliers	0.44	0.00	0.88	1.35
Favored	94.27	96.43	91.59	93.27

Table S5. Similarity of OXA-57 to Other Class-D β-Lactamases*.

Protein	PDB accession	Resolution (Å)	Sequence identity (%)	Cα RMSD (Å)
OXA-1	1M6K	1.50	34.67	1.41
OXA-10	1FOF	2.00	25.36	1.76
OXA-23	4K0X	1.61	22.66	1.50
OXA-24	3G4P	1.97	18.75	1.62
OXA-48	4S2P	1.70	27.36	1.62
OXA-58	4OH0	1.30	21.95	1.81
OXA-D84/YbxI	6NI0	2.30	89.66	0.60

*Comparisons used the 2.0 Å structure of uncomplexed OXA-57 (pdb 9HPT). RMSD values were generated using SSMSuperpose *(9)*.

Table S6. B-Factors for OXA-57 Lys56 Carbamate.*

	Uncomplexed OXA-57<br><br>PDB: 9HPT	Uncomplexed OXA-57<br><br>PDB: 9HPU	OXA-57: avibactam<br><br>PDB: 9HPY	OXA-57: meropenem<br><br>PDB: 9HPW
Atom	B-factor ($Å^2$)	B-factor ($Å^2$)	B-factor ($Å^2$)	B-factor ($Å^2$)
KCX56 N_ζ	50.30	27.75	66.83	76.69
KCX56 CX	54.01	28.90	67.38	76.41
KCX56 OQ1	51.26	27.57	69.48	76.94
KCX56 OQ2	50.47	27.36	74.43	76.68

*Lysine-carbamate occupancy was set to 1.0 in all cases.

Table S7. Bridging Water Interactions Between the C3 Carboxylate Group of Meropenem and T202.

Water-mediated acceptor:donor/acceptor	R1*	R2*	R3*	R4*	R5*
MEM53@*O01*:T202@*OG1*	5787			4384	
MEM53@*O01*:T202@*HG1*	5197	12136	7952	6649	13194
MEM53@*O03*:T202@*OG1*	10709		5417		
MEM53@*O03*:T202@*HG1*	6359	6428	7159	6255	6033
MEM53@*O01*:MEM53@*H01*:T202@*OG1*		12062	10039		10026
MEM53@*O03*:MEM53@*H01*:T202@*OG1*	6094	8446	12298		5412

*Values correspond to the number of frames (total 42,501) in which a bridging water is present.

Table S8. Similarity of OXA-57:Avibactam Structure to Other OXA:Avibactam Complex Structures.

Protein	PDB accession code	Resolution (Å)	Cα RMSD* (Å)
OXA-10:avibactam	4S2O	1.70	1.72
OXA-24:avibactam	4WM9	2.40	1.56
OXA-48:avibactam	4S2N	2.00	1.61

*RMSD values were generated using SSMSuperpose *(9)*

Table S9. Similarity of OXA-57:Meropenem Structure to Other OXA:Carbapenem Complexe Structures.

Protein	PDB accession code	Resolution (Å)	Cα RMSD (Å)*
OXA-1:doripenem	3ISG	1.40	1.45
OXA-10:imipenem	6SKP	1.89	1.70
OXA-23:meropenem	4JF4	2.14	1.58
OXA-48:meropenem	6P98	1.75	1.58

*RMSD values were generated using SSMSuperpose *(9)*

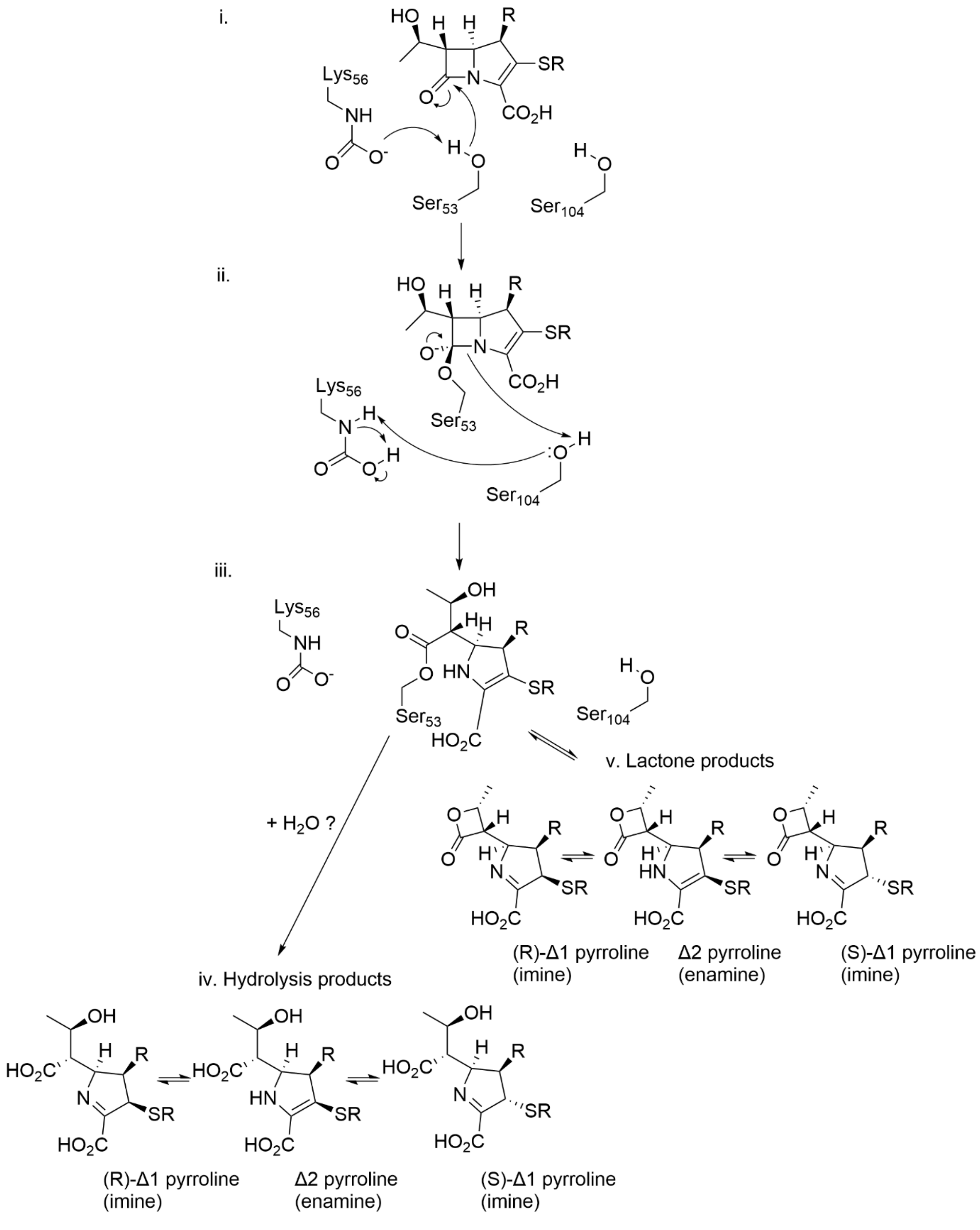

Figure S1. Possible Mechanisms of Reaction of Carbapenem Substrates with OXA β-Lactamases. i. S53 is activated for nucleophilic attack on the β-lactam carbonyl by carbamylated K56. **ii., iii.** Nucleophilic attack results in a tetrahedral intermediate that resolves to the ring-opened acyl-enzyme complex *via* protonation of the lactam nitrogen by S104. **iv.** The acylenzyme reacts with a deacylating water molecule to yield hydrolyzed products, which can exist in alternative tautomeric and epimeric forms. **v.** The alternative lactone products (which can also exist in alternative tautomeric and epimeric forms) are reversibly generated through reaction of the acyl-enzyme complex with the hydroxyethyl side chain alcohol.

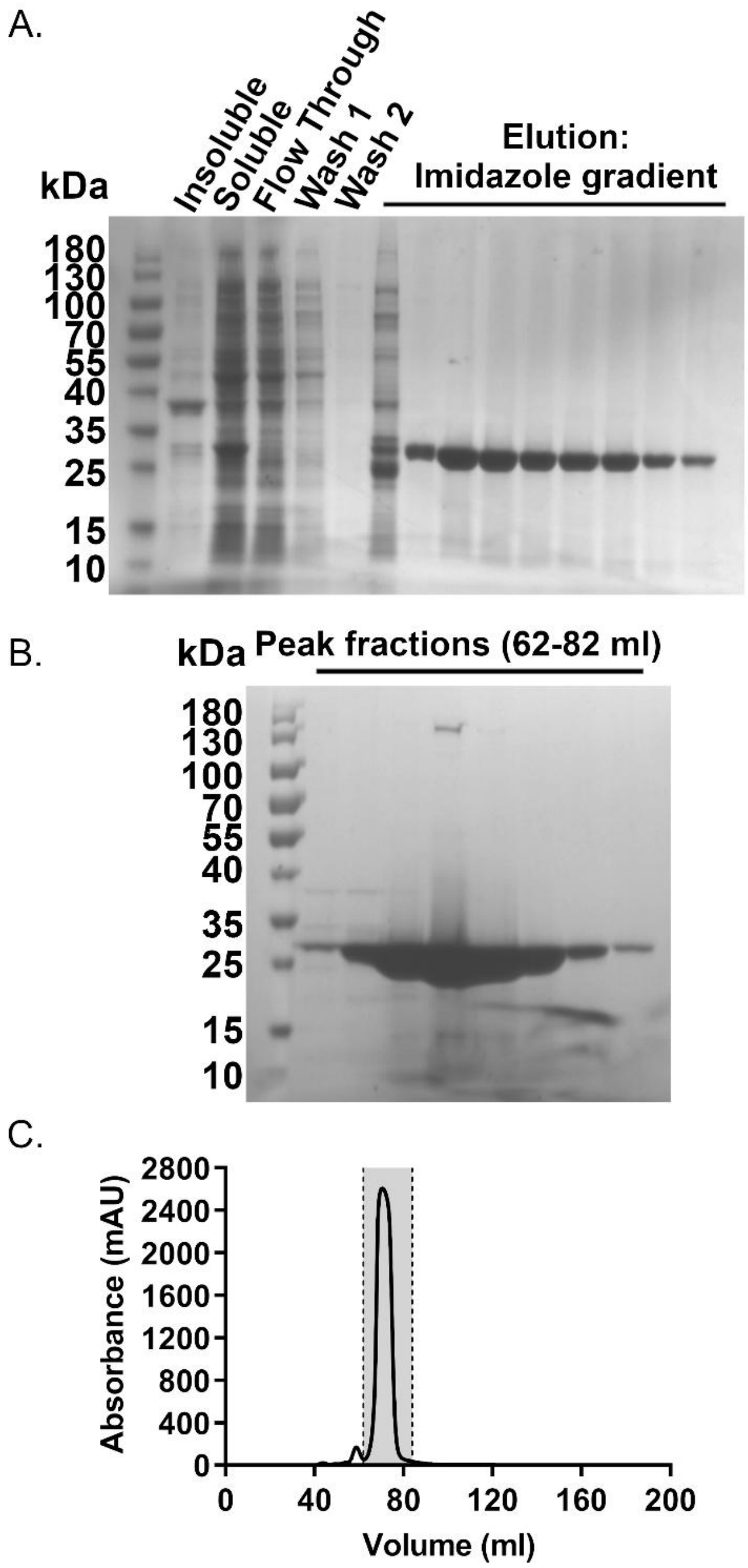

Figure S2. Purification of Recombinant OXA-57. A. SDS-PAGE showing fractions from IMAC purification with elution on a linear imidazole gradient. B. SDS-PAGE showing peak fractions from Size Exclusion Chromatography (SEC) on Superdex 75 column. C. Representative chromatogram of HiLoad 16/600 Superdex 75 pg SEC monitored by absorbance at 280 nm. Shaded gray region of the chromatogram represents the peak fractions shown in B, OXA-57 elutes as a single symmetrical peak of approximately 27 kDa molecular mass.

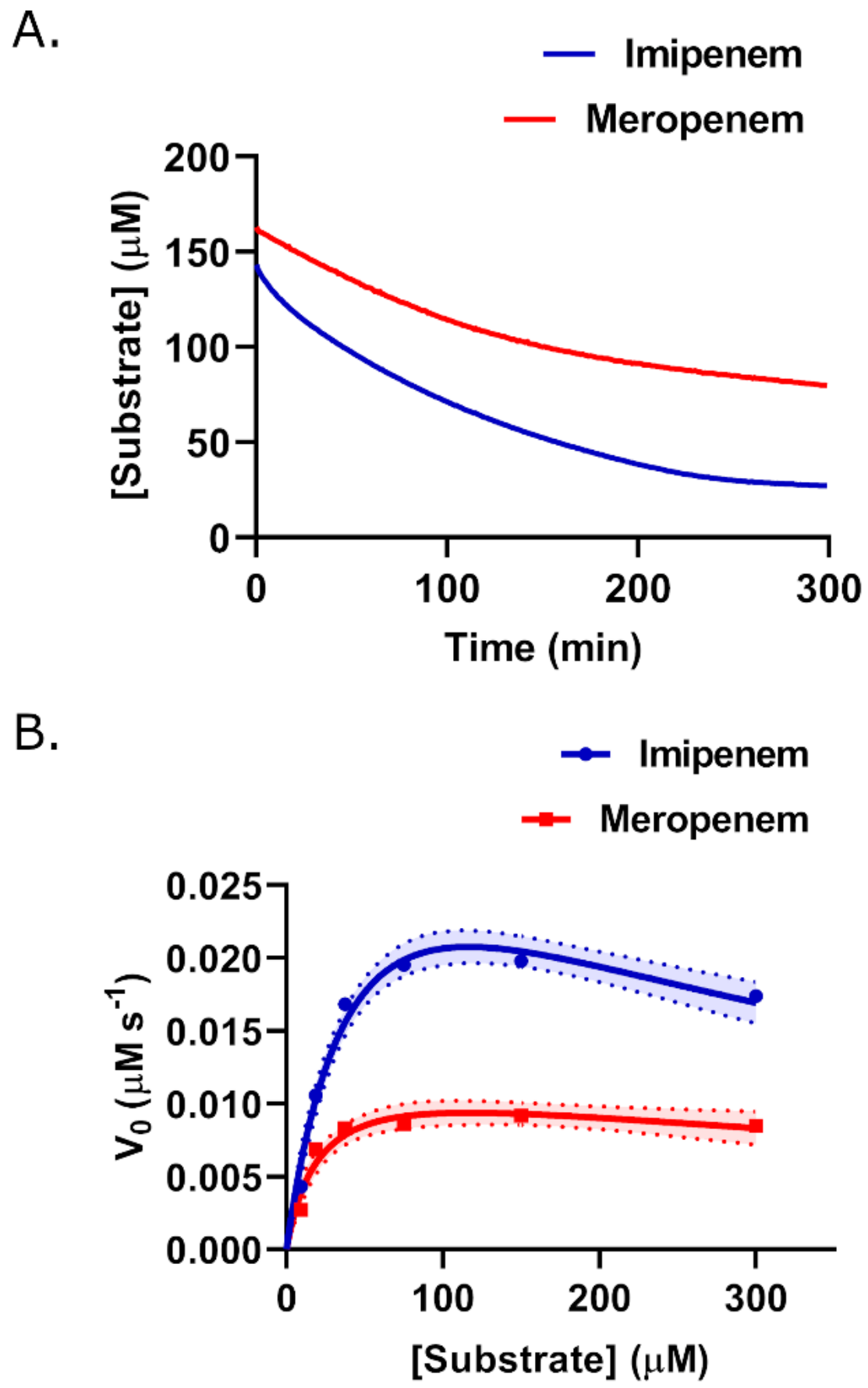

Figure S3. Hydrolytic activity of OXA-57 against carbapenems. A. Representative progress curves of imipenem (blue) and meropenem (red) hydrolysis at 150 μM with 1 μM OXA-57. B. Michaelis-Menten plots of imipenem (blue) and meropenem (red) hydrolysis kinetics. Each measurement was carried out in triplicate, dotted lines represent the 95 % confidence bands from nonlinear regression fitting.

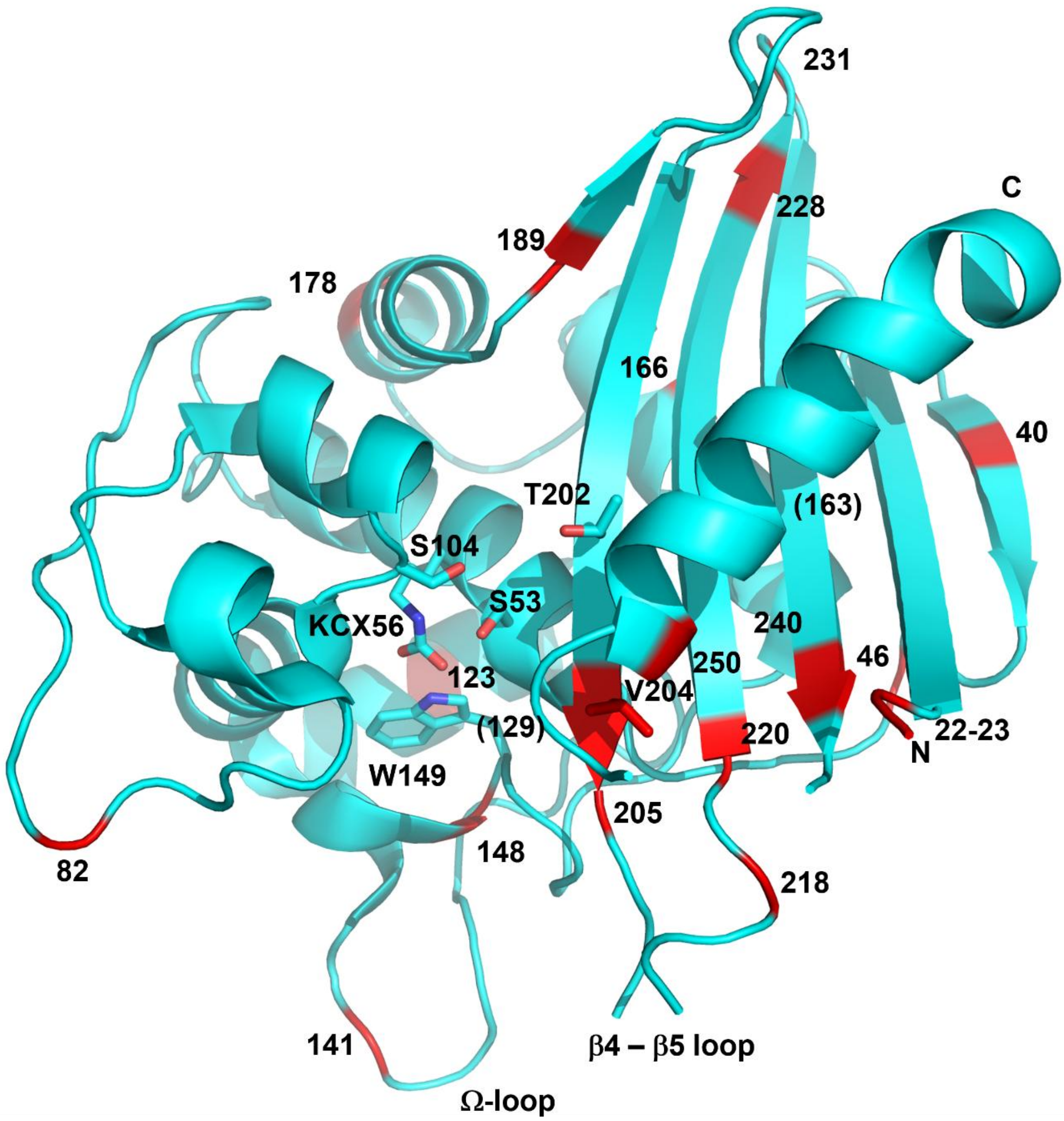

Figure S4. Positions of Sequence Variation between OXA-57 and *B. thailandensis* YbxI. Structure of uncomplexed OXA-57 (pdb 9HPT, this work) with positions of variation with respect to YbxI (pdb 6NI0) shown in red. Residue numbers are those for OXA-57. Numbers in parentheses give approximate locations of differing residues not visible in this view. Active site residues are shown as sticks. This Figure was created using Pymol (www.pymol.org).

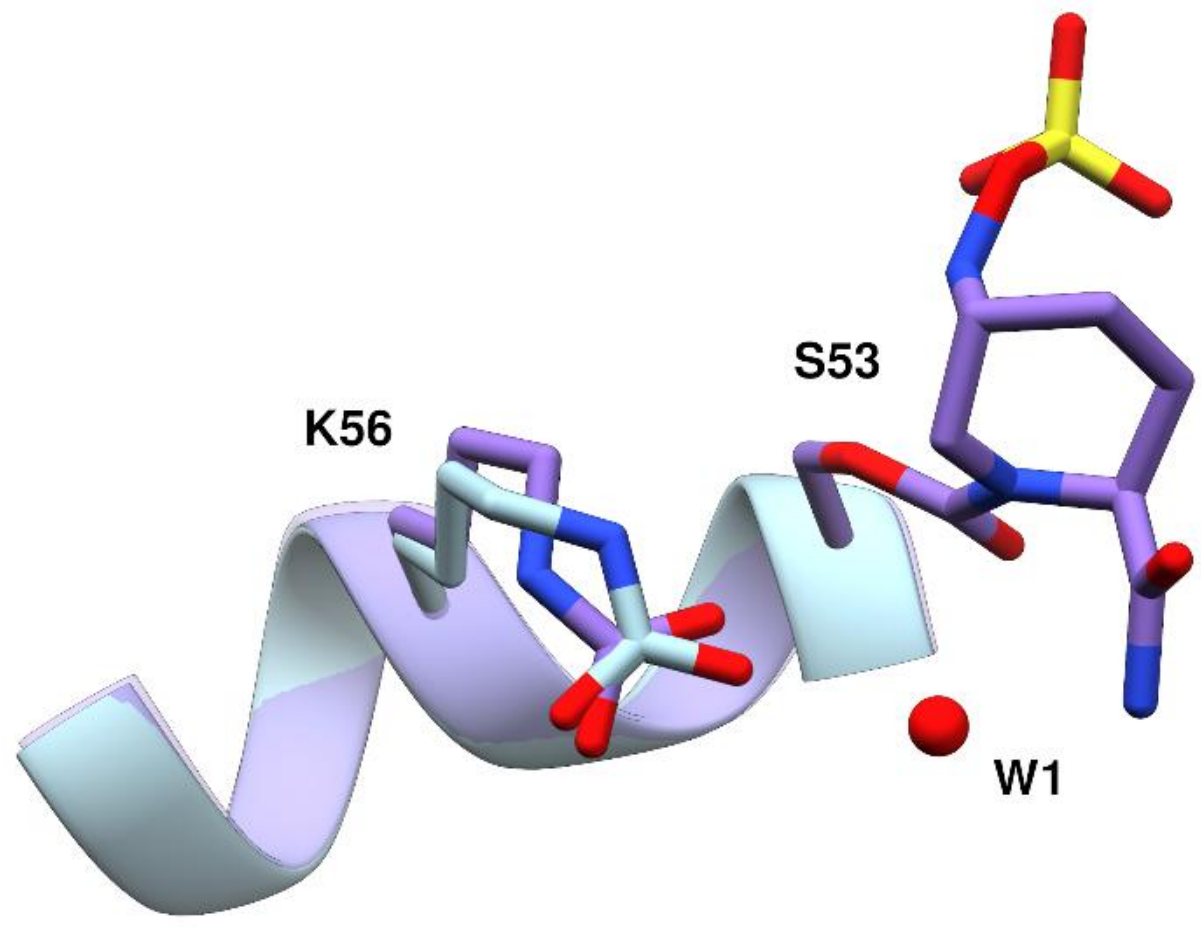

Figure S5. Change in orientation of carbamylated lysine K56 upon avibactam binding. OXA-57 protein chain (cartoon) and K56 side chain (sticks) are shown in light blue and purple in the uncomplexed and OXA-57:avibactam structures, respectively. Bound avibactam is shown as sticks, with carbon atoms purple and non-carbon atoms colored by atom type. Water molecules shown as red spheres.

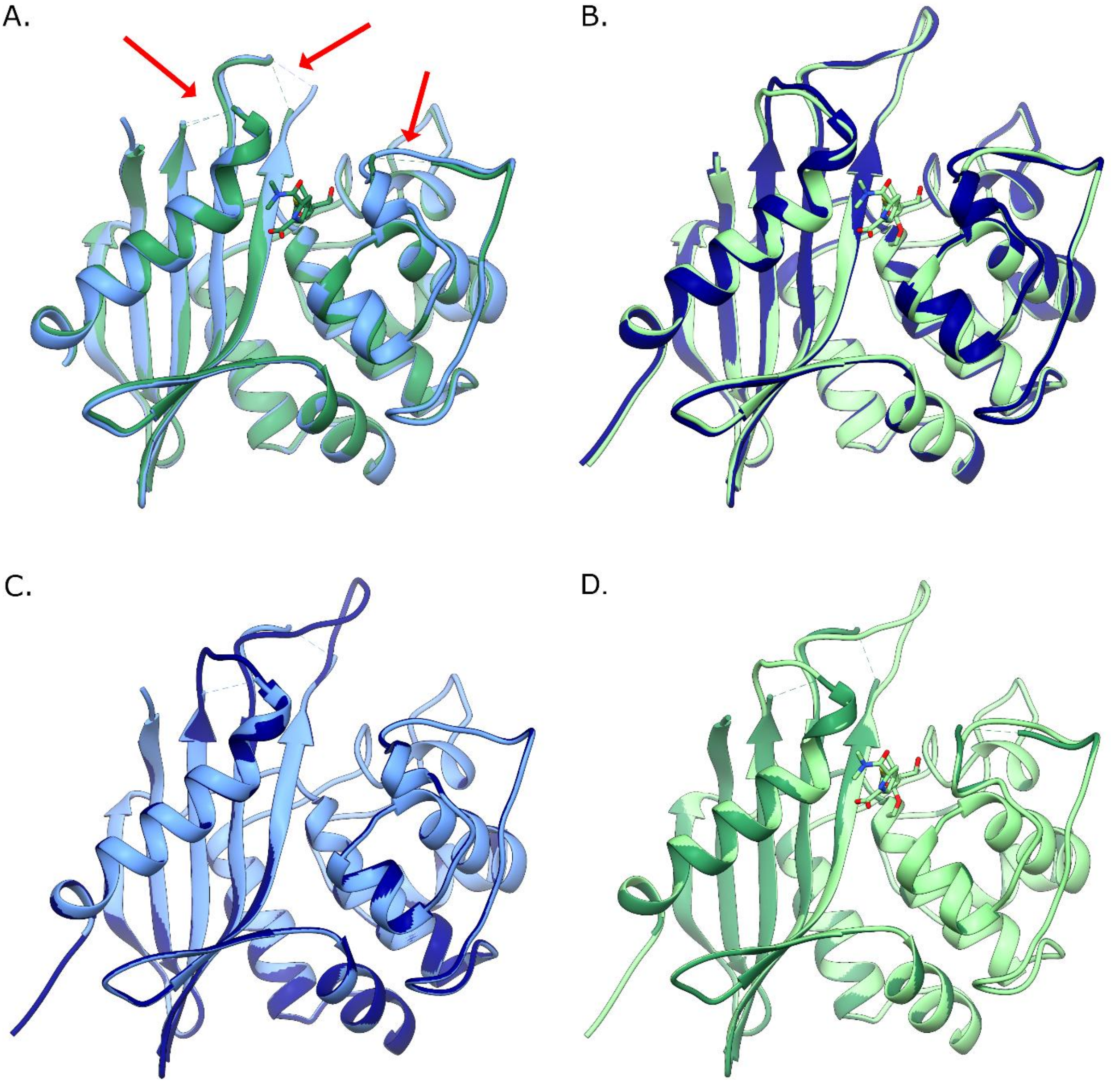

Figure S6. OXA-57 Structures Used for Molecular Dynamics Simulations. A. X-ray crystal structures (this work) of uncomplexed OXA-57 (PDB: 9HPT; blue) and OXA-57: meropenem complex (PDB: 9HPW; green). Red arrows indicate positions where unmodelled loops in the crystal structures were built for simulations. B. Models of uncomplexed OXA-57 (blue) and meropenem complex (mint) with β4 - β5 and β6 - α8, and α1 - α2, β4 - β5 and β6 - α8 loops, respectively, built using MODELLER*(10)*. C. Uncomplexed OXA-57 crystal structure (blue) and after addition of modelled loops (navy). D. OXA-57:meropenem complex crystal structure (green) and after addition of modelled loops (mint).

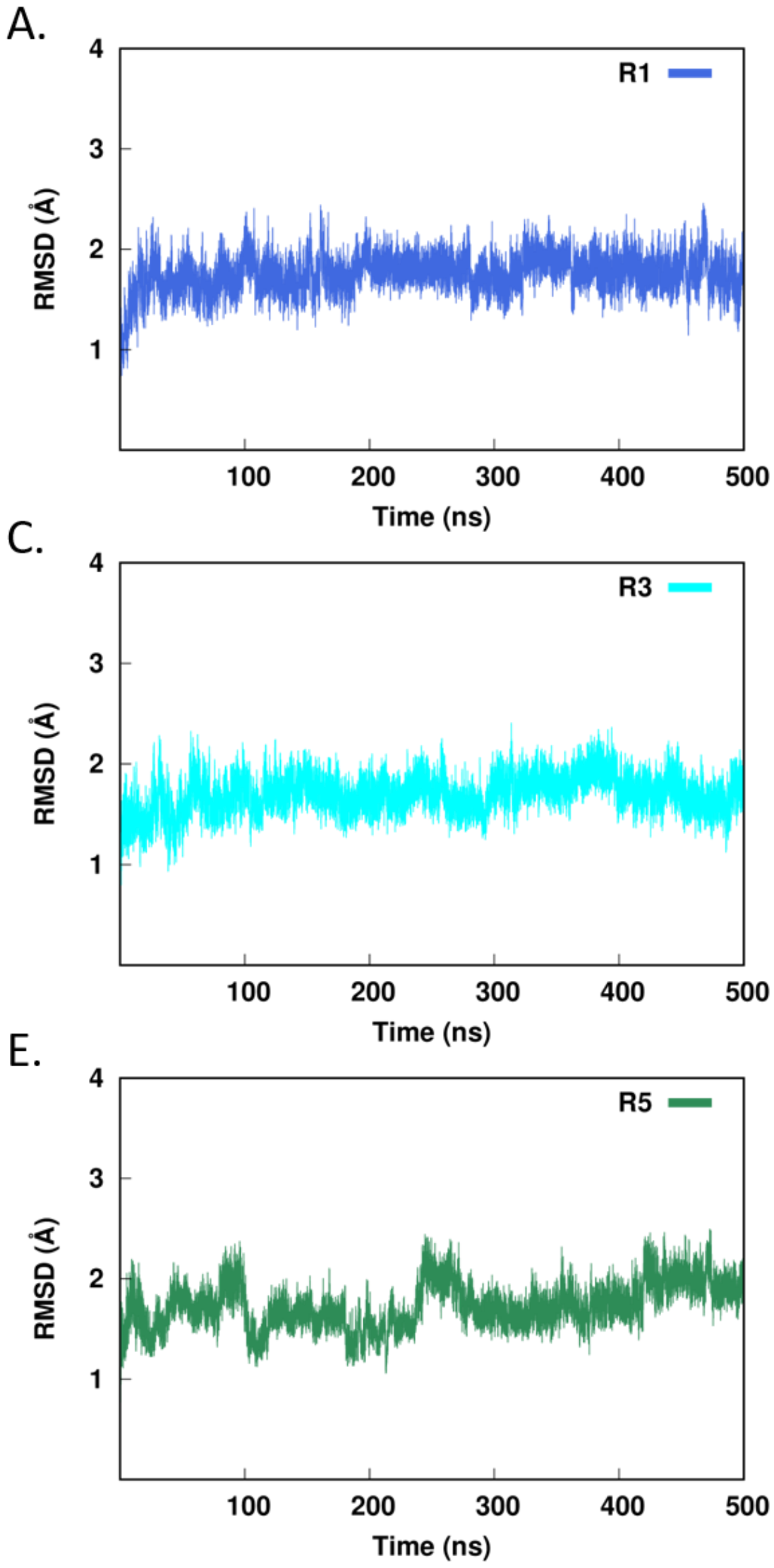

Figure S7. Stability of Molecular Dynamics Simulations of Uncomplexed OXA-57. Time series of backbone atom (N, Cα and C) RMSD values, compared to the starting structure, over 500 ns trajectories for repeat simulations one to five (R1-R5; panels A-E) of uncomplexed OXA-57.

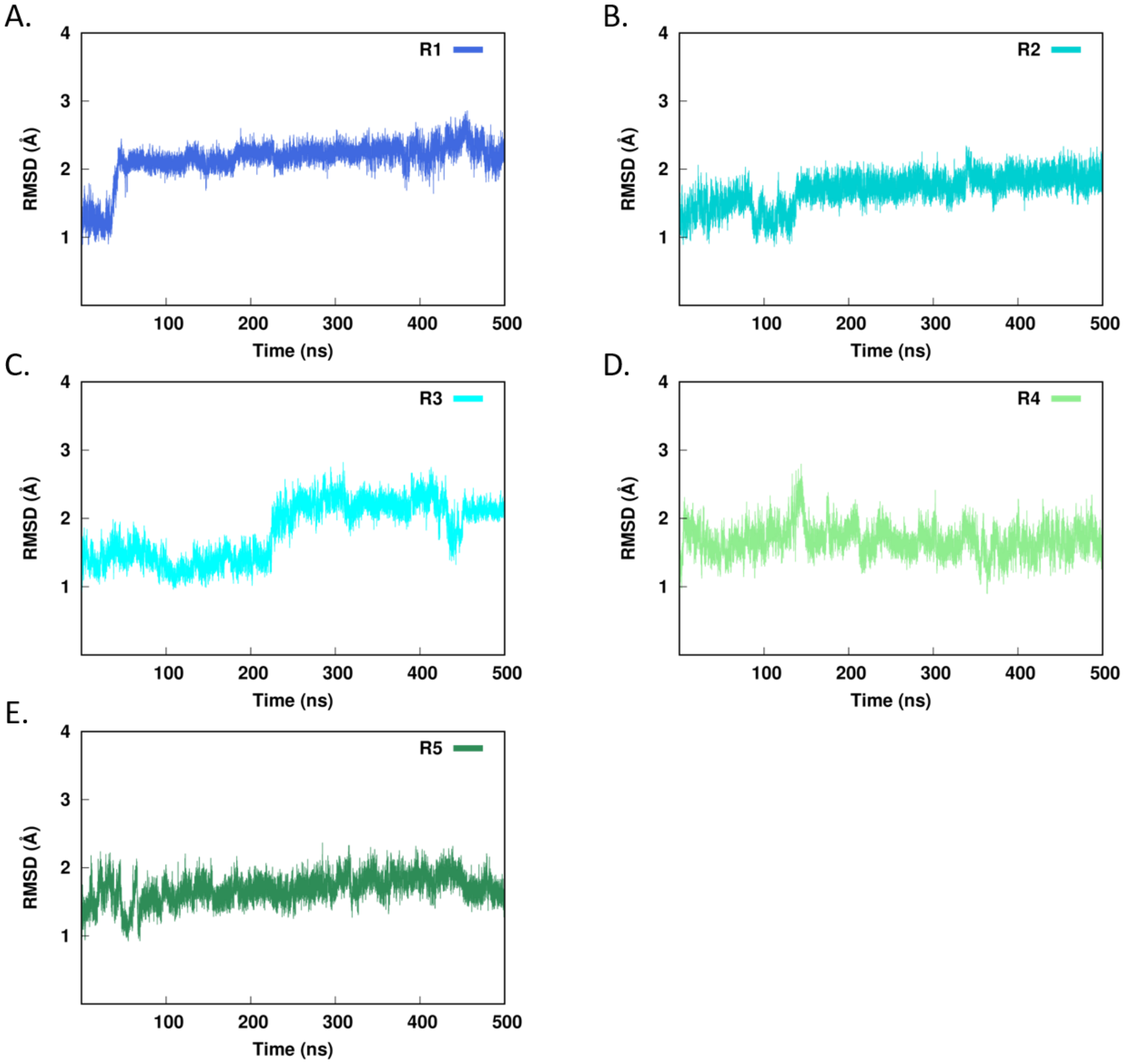

Figure S8. Stability of Molecular Dynamics Simulations of OXA-57:Meropenem Complex Time series of backbone atom (N, Cα and C) RMSD values, compared to the starting structure, over 500 ns trajectories for repeat simulations one to five (R1-R5; panels A - E) of the OXA-57:meropenem complex.

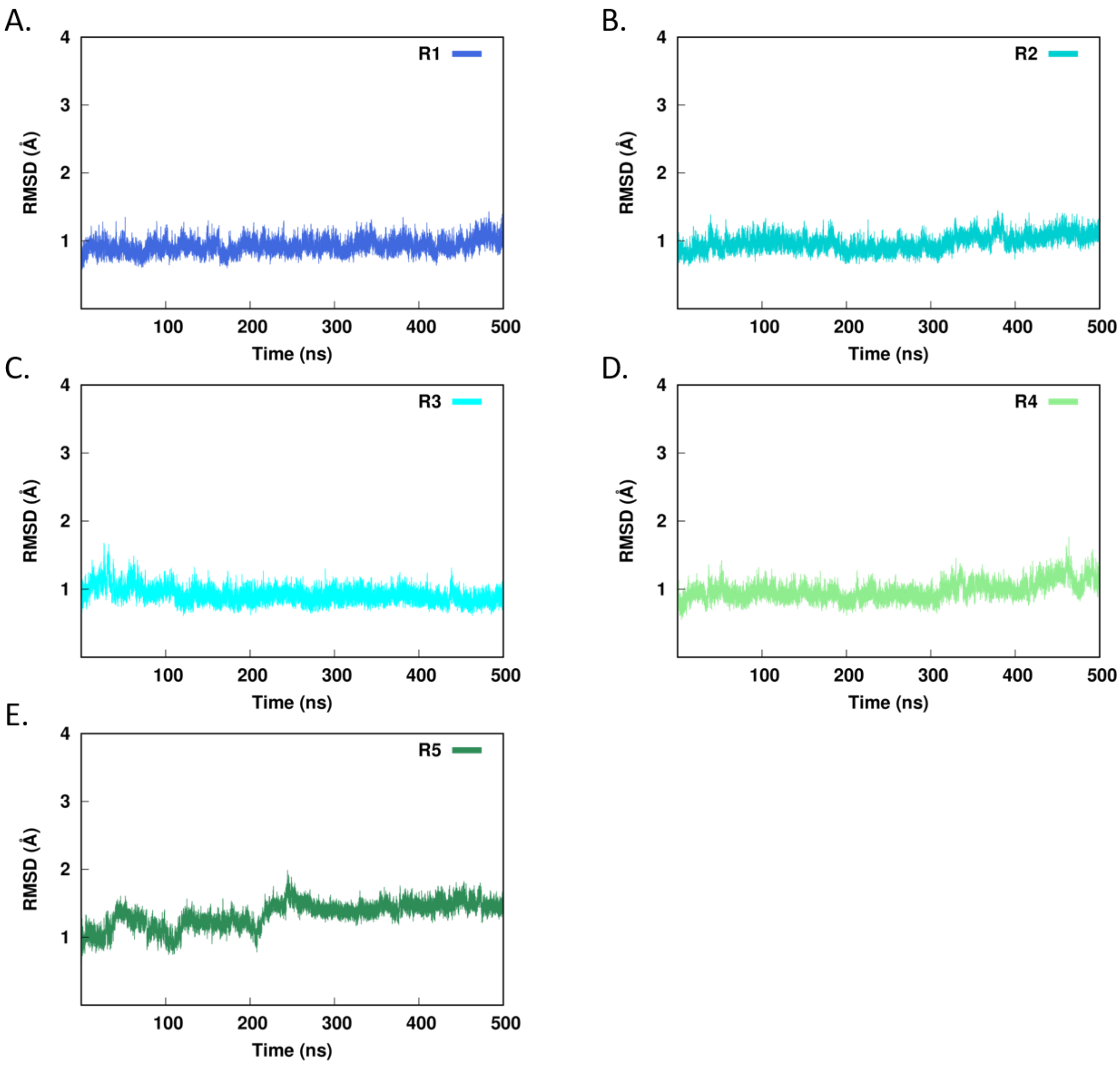

Figure S9. Stability of Molecular Dynamics Simulations of Uncomplexed OXA-57 (Core Regions). Time series of uncomplexed OXA-57 backbone atom (N, Cα and C) RMSD values, compared to the starting structure, over 500 ns trajectories for repeat simulations one to five (R1-R5; panels A-E), excluding residues corresponding to the β4 - β5 loop (S205 - S220) and the C-terminus (L266 - R269).

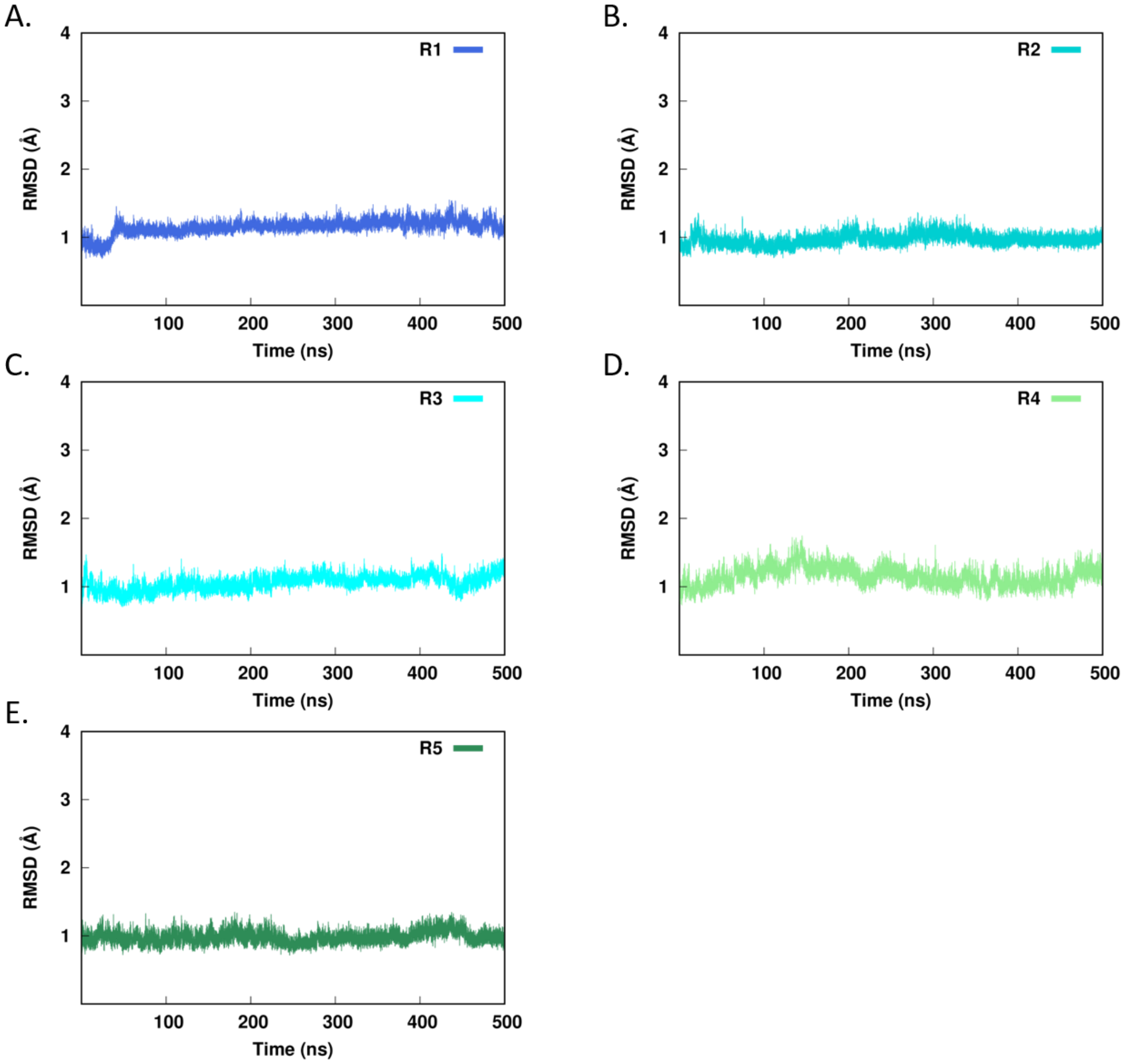

Figure S10. Stability of Molecular Dynamics Simulations of OXA-57:Meropenem Complex (Core Regions). Time series of backbone atom (N, Cα and C) RMSD values for the OXA-57:meropenem complex, compared to the starting structure, over 500 ns trajectories for repeat simulations one to five (R1 - R5; panels A - E), excluding residues corresponding to the β4 - β5 loop (S205 - S220) and the C-terminus (L266 - R269).

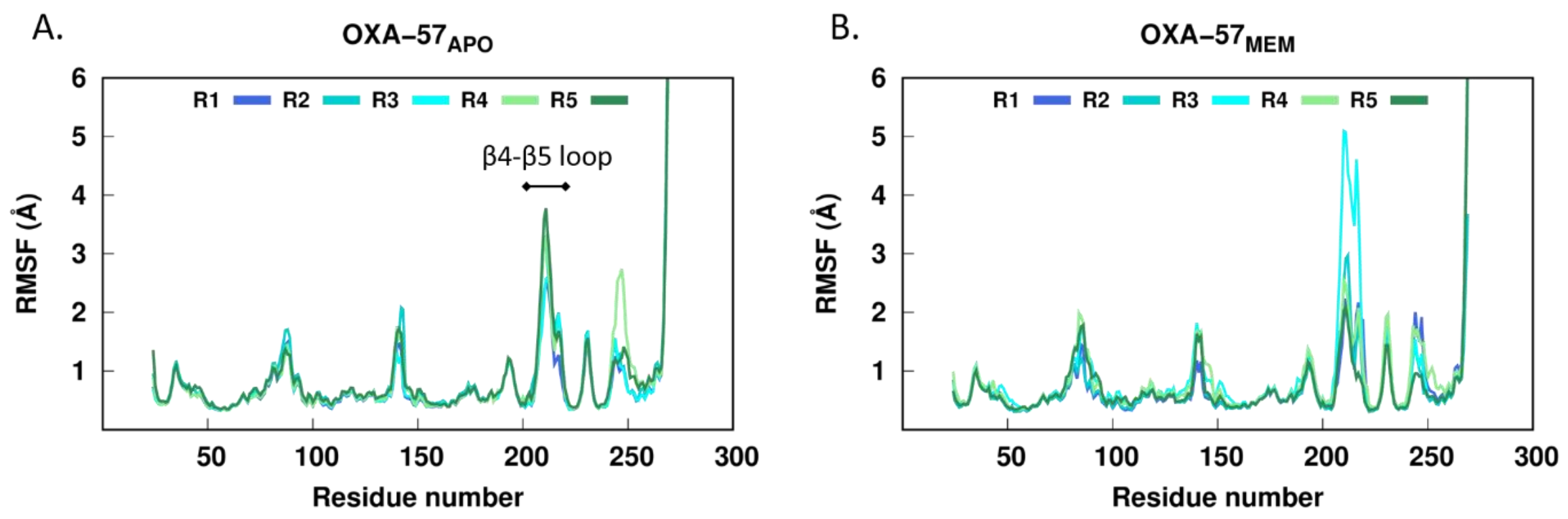

Figure S11. Backbone Dynamics of OXA-57. Backbone atom (N, Cα, C) RMSF values calculated over 425 ns MD trajectories (excluding first 75 ns for equilibration) using the average backbone (N, Cα, C) coordinates as reference for simulations of: A. uncomplexed OXA-57 and B. OXA-57:meropenem complex, respectively. The largest RMSF peak corresponds to the β4 - β5 loop.

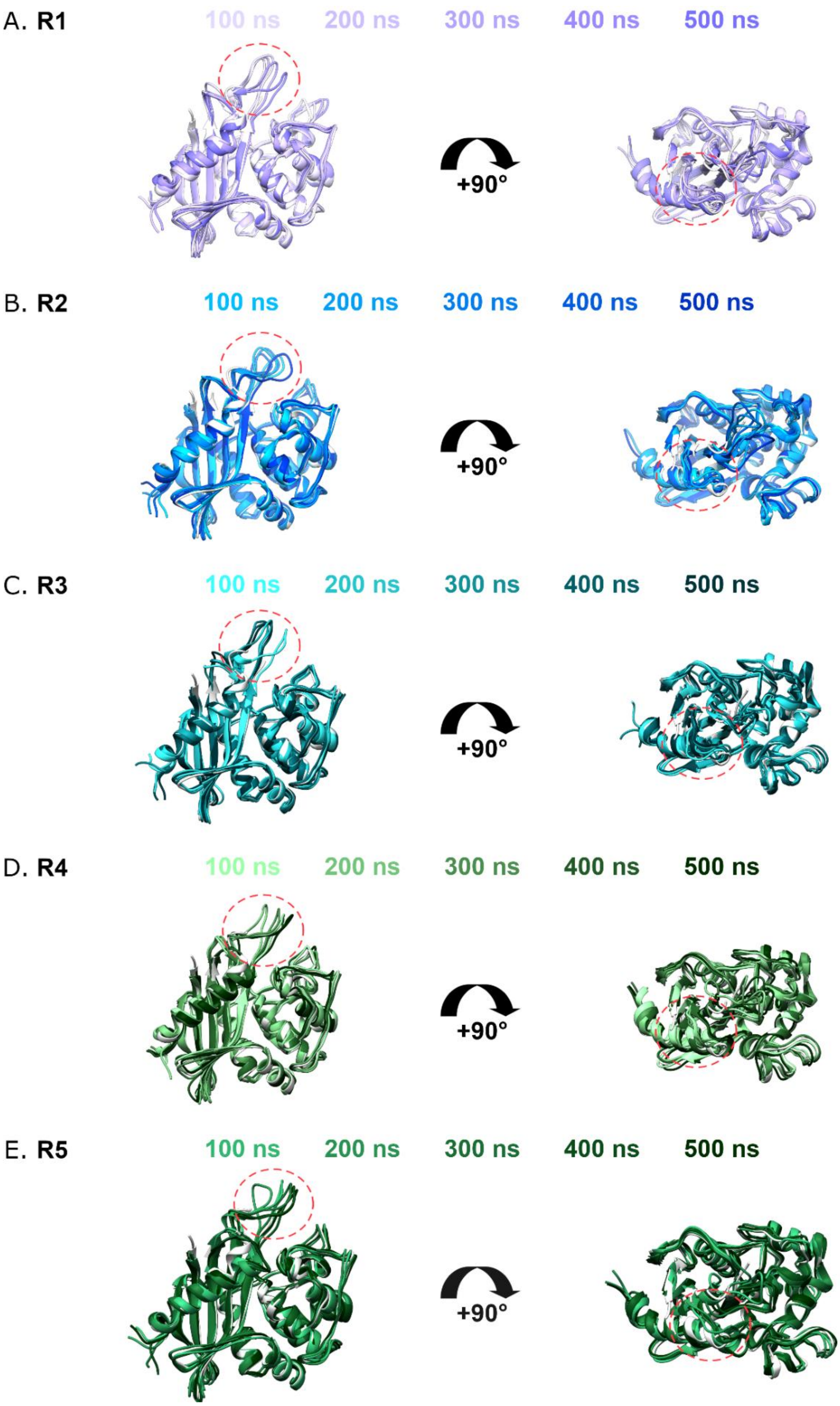

Figure S12. Snapshots of Uncomplexed OXA-57 During MD Simulations. Superposed structures from 100 ns snapshots throughout the 500 ns trajectories of simulation repeats one to five (R1-R5, A-E) of uncomplexed OXA-57. Coloring: R1 purple, R2 blue, R3 cyan, R4 mint, R5 green. Darkening colors indicate successive 100 ns snapshots of each simulation. Crystal structure is shown in white with the β4-β5 loop circled in red.

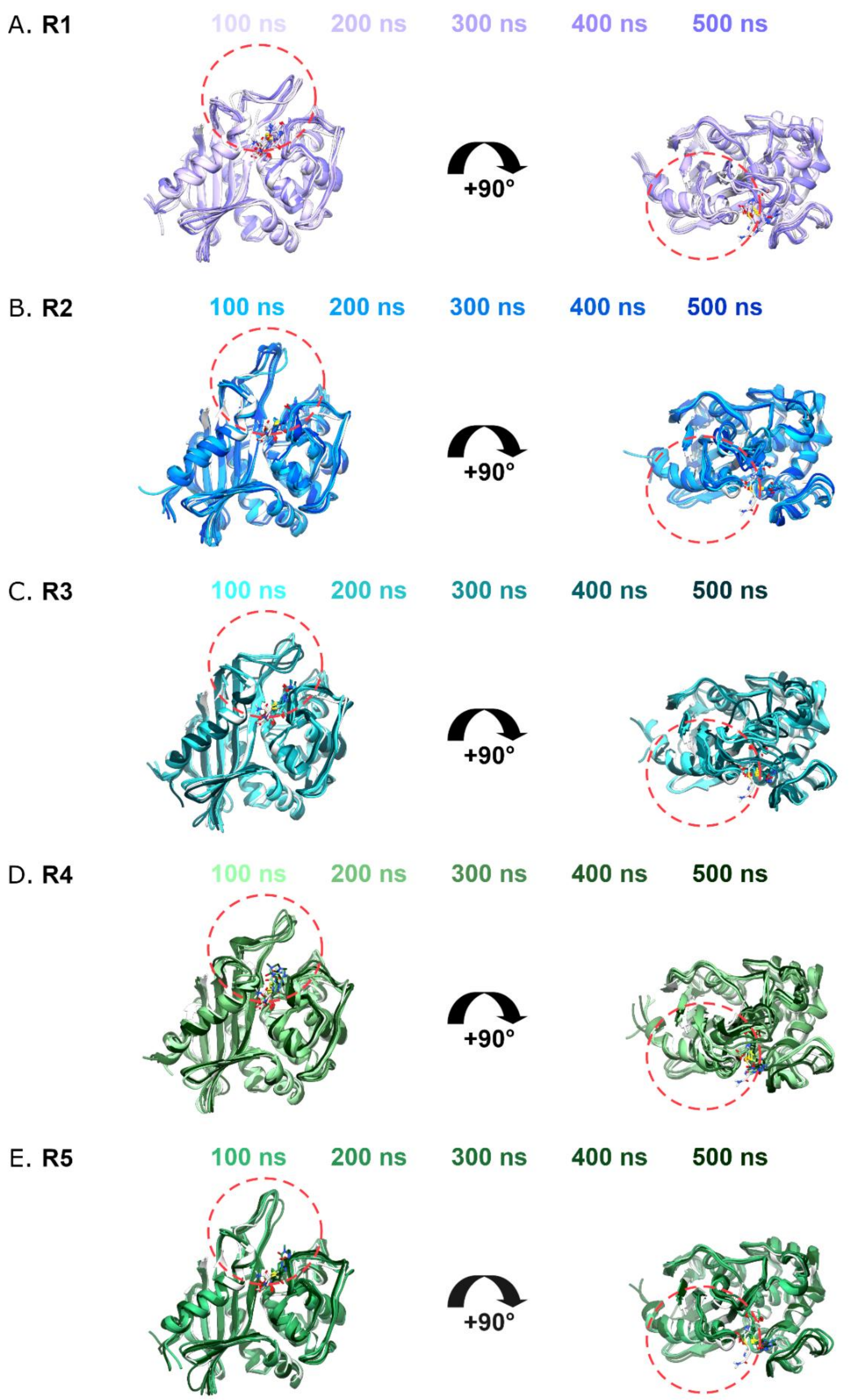

Figure S13. Snapshots of OXA-57:Meropenem Complex During MD Simulations. Superposed structures from 100 ns snapshots throughout the 500 ns MD trajectories of the OXA-57:meropenem complex for repeat simulations one to five (R1 - R5, panels A - E). Coloring is as follows: R1 in purple, R2 in blue, R3 in cyan, R4 in mint and R5 in green. Data from each increasing 100 ns snapshot of the simulation is depicted as the repeat color becoming progressively darker. Crystal structure is shown in white and the β4 - β5 loop is circled in red.

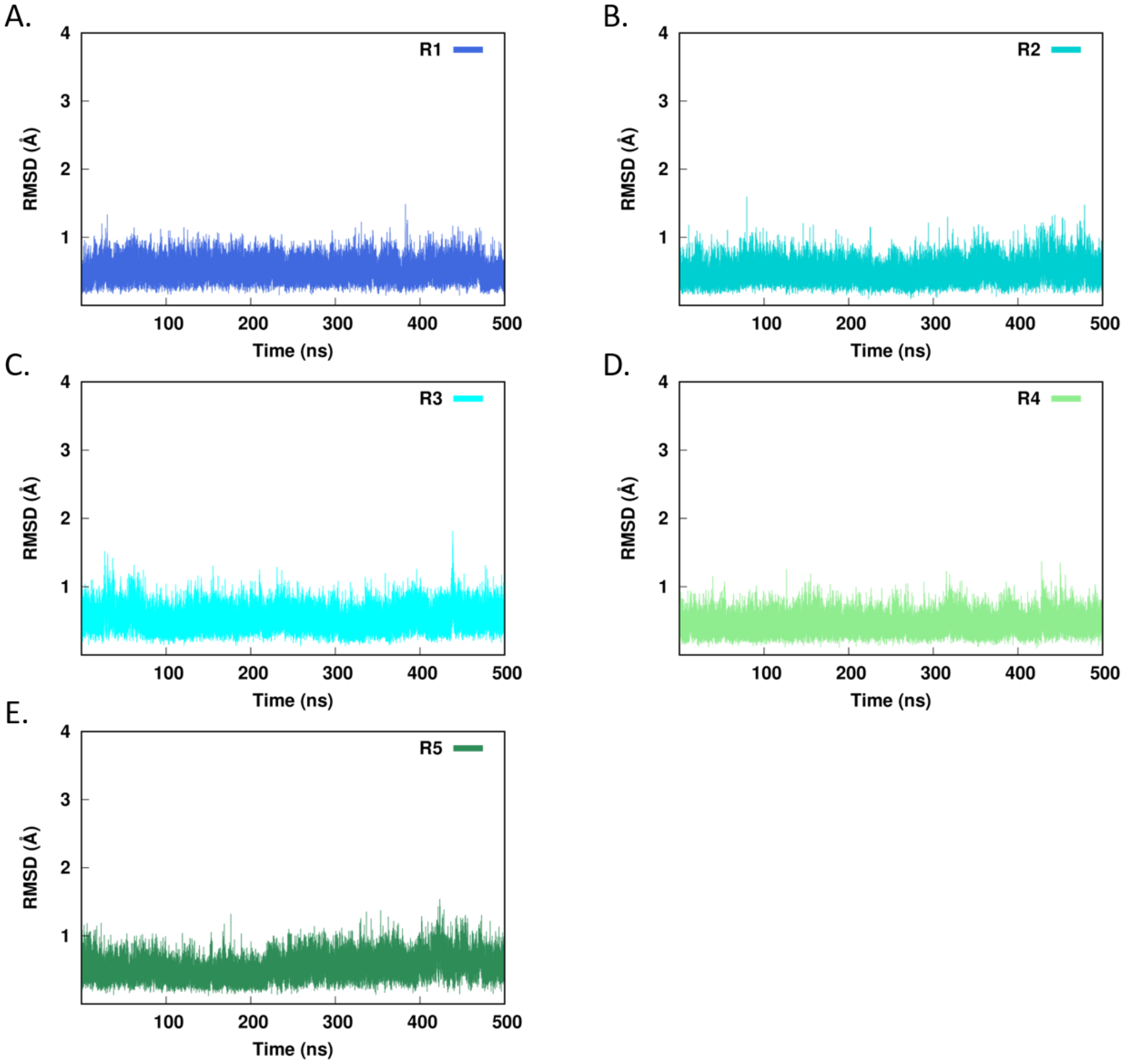

Figure S14. Stability of Active Site Carbamyl-K56 During Molecular Dynamics Simulations of Uncomplexed OXA-57. Time series of RMSD values excluding backbone atoms (N, Cα, C and O), compared to the starting structure, for the carbamylated active site lysine (K56), over 500 ns trajectories for repeat simulations one to five (R1 - R5; panels A - E) for uncomplexed OXA-57.

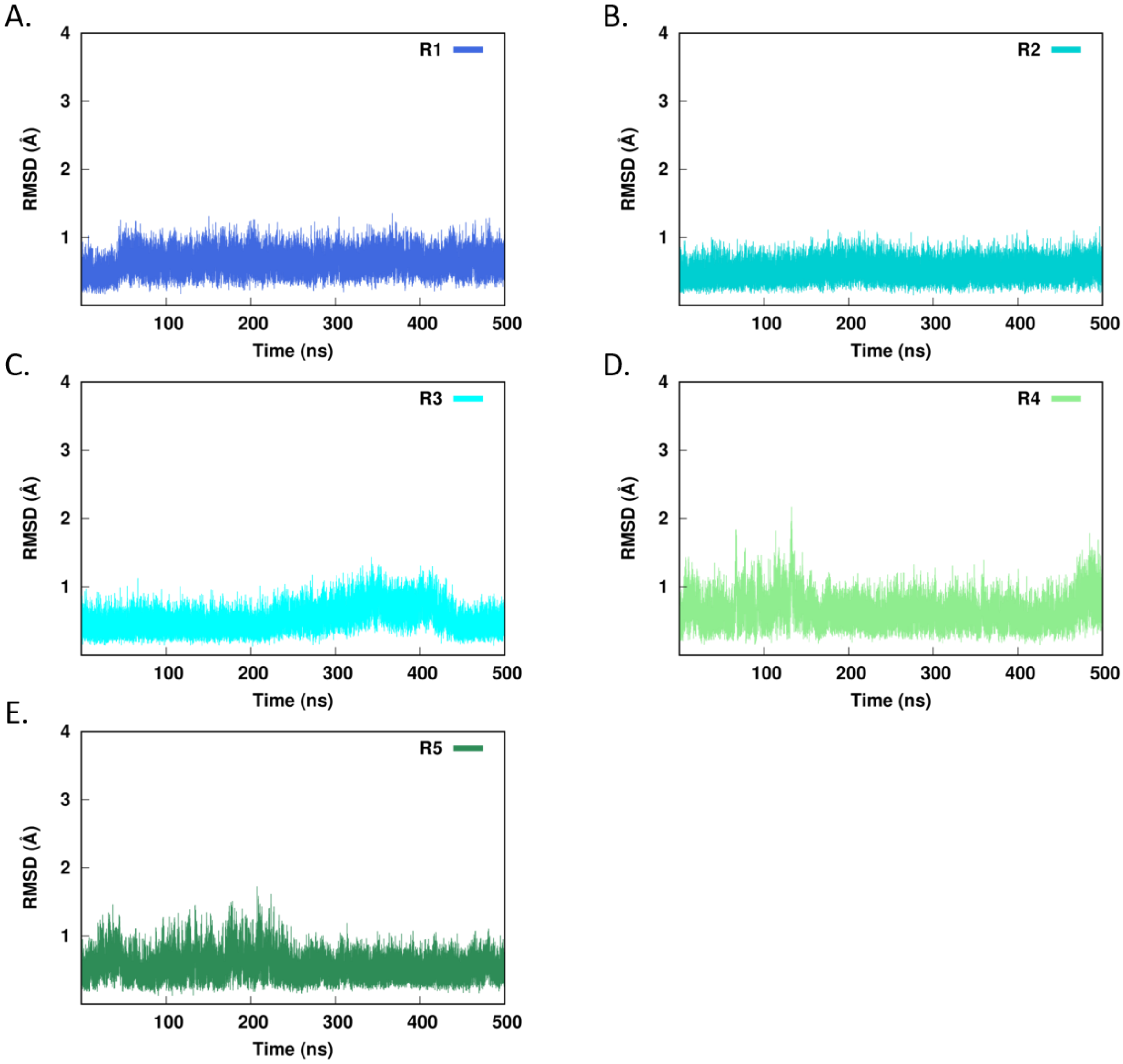

Figure S15. Stability of Active Site Carbamyl-K56 During Molecular Dynamics Simulations of OXA-57:Meropenem Complex. Time series of RMSD values excluding backbone atoms (N, Cα, C and O), compared to the starting structure, for the active site carbamylated lysine (K56) over 500 ns trajectories for repeat simulations one to five (R1 - R5; panels A - E) of the OXA-57:meropenem complex.

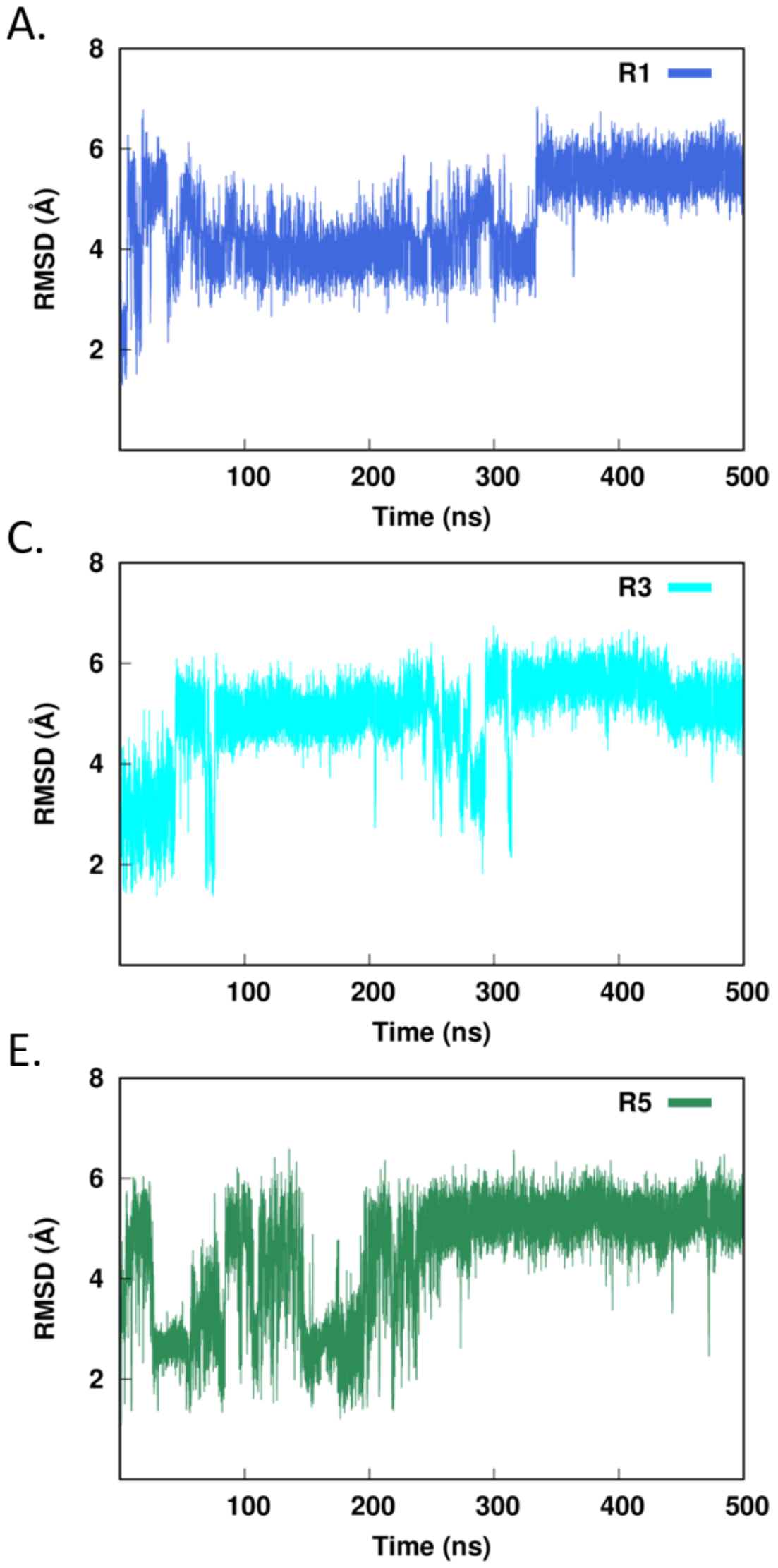

Figure S16. Stability of Bound Meropenem During Molecular Dynamics Simulations of OXA-57:Meropenem Complex. Time series of RMSD values, compared to the starting structure and excluding backbone atoms (N, Cα, C and O) of the acylated serine residue, for bound meropenem (MEM53) over 500 ns trajectories for repeat simulations one to five (R1 - R5; panels A-E) of the OXA-57:meropenem complex.

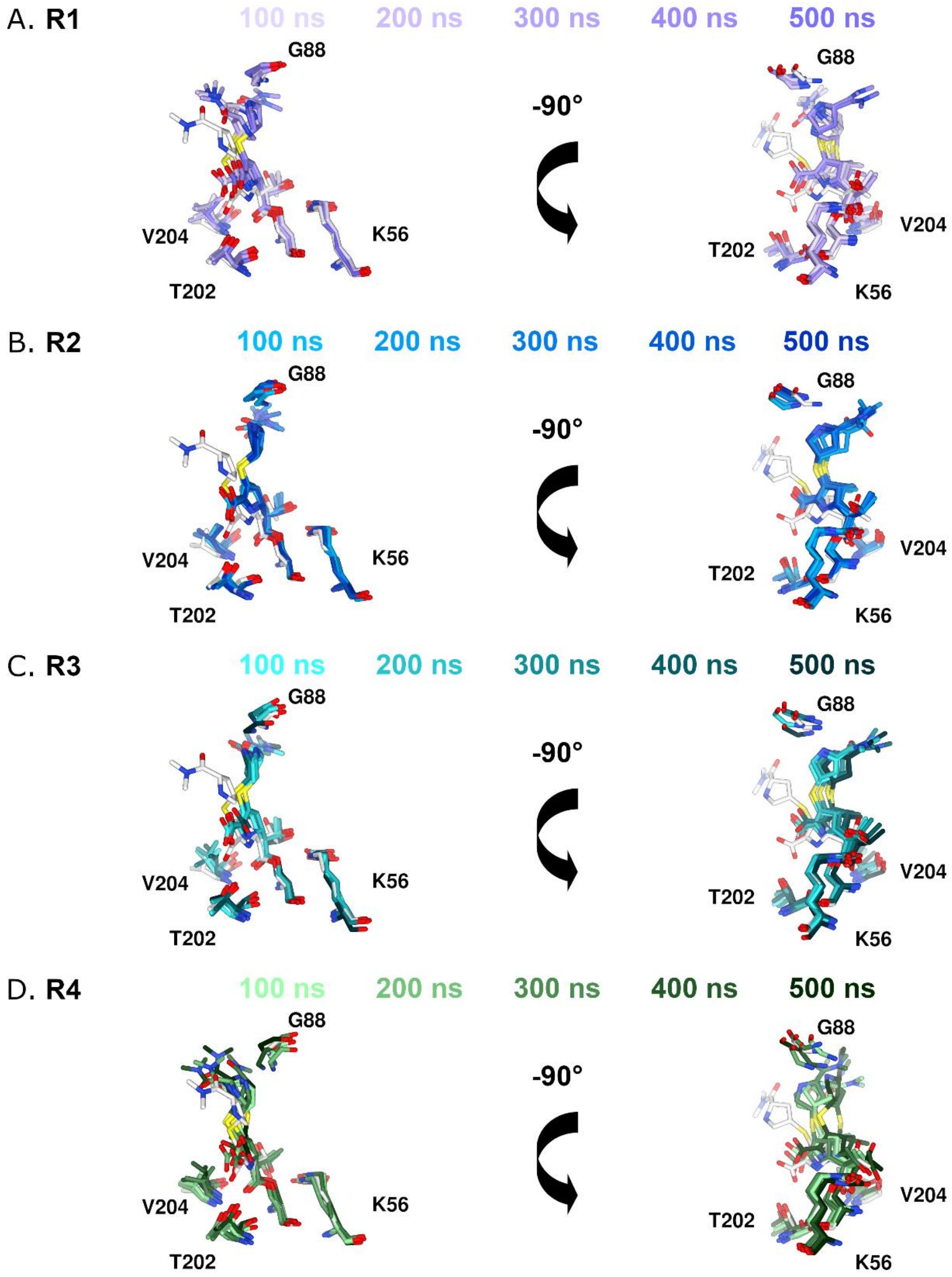

Figure S17. Orientations of Bound Meropenem During MD Simulations. Superposed structures of meropenem at 100 ns intervals throughout the 500 ns MD trajectories for repeat simulations one to four (R1-R4; panels A-D) of the OXA-57:meropenem complex. Coloring is as follows: R1 in purple, R2 in blue, R3 in cyan and R4 in mint. Data from each increasing 100 ns snapshot of the simulation is depicted as the repeat color becoming progressively darker. The crystal structure is shown in white.

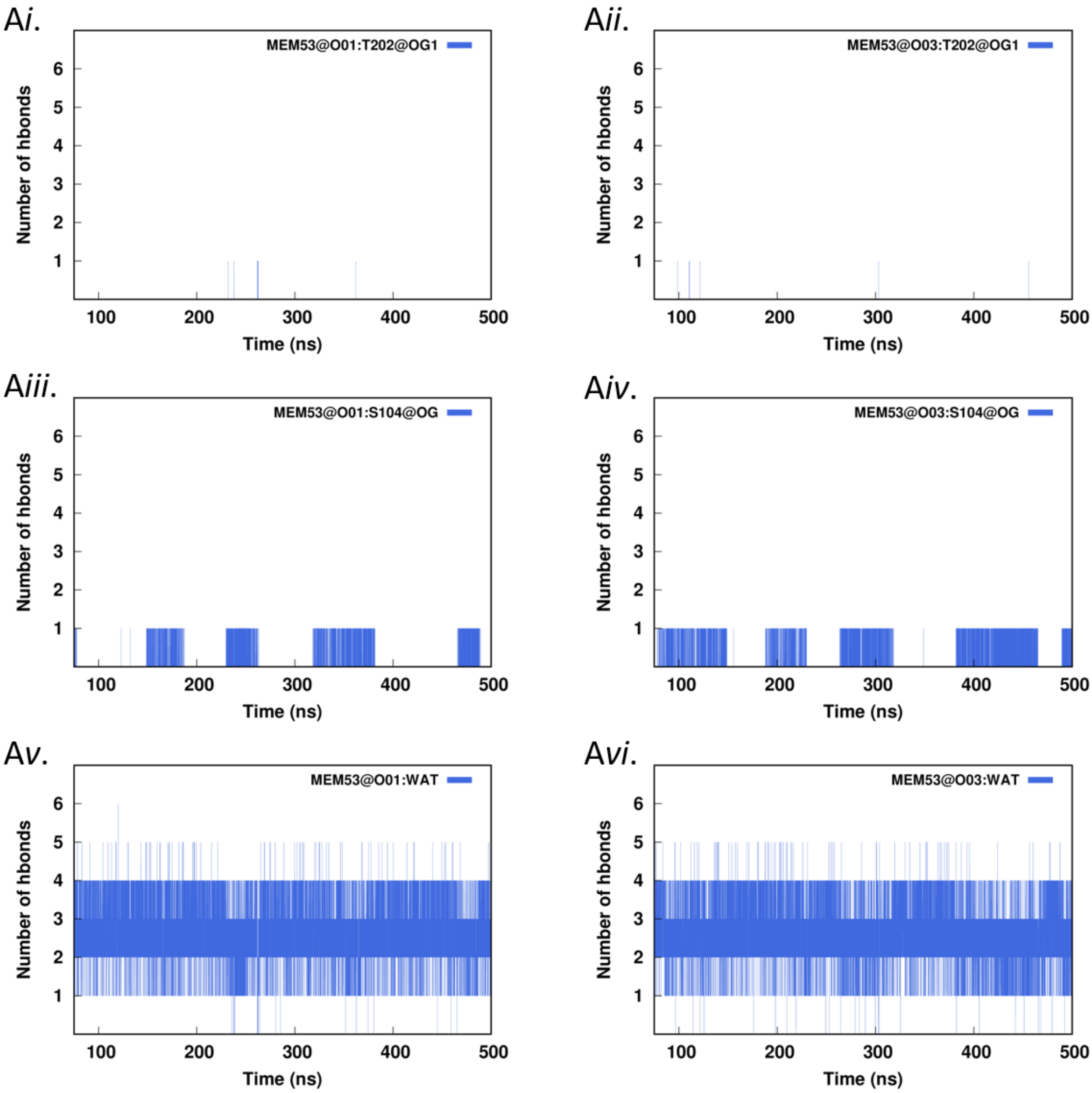

Ai.
MEM53@O01:T202@OG1
Aii.
MEM53@O03:T202@OG1
Aiii.
MEM53@O01:S104@OG
Aiv.
MEM53@O03:S104@OG
Av.
MEM53@O01:WAT
Avi.
MEM53@O03:WAT
Number of hbonds
Time (ns)

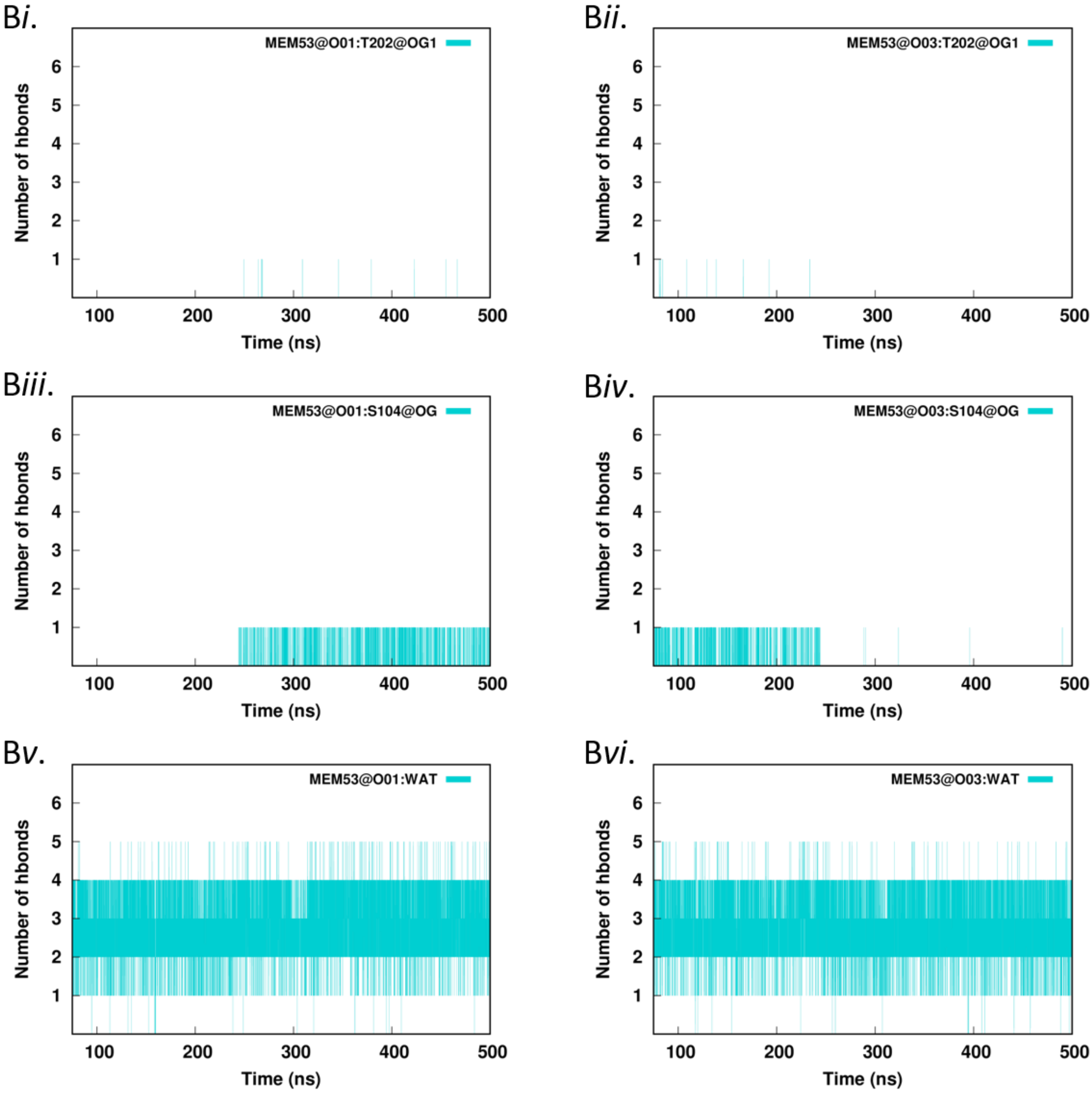
Bi.
MEM53@O01:T202@OG1
Bii.
MEM53@O03:T202@OG1
Biii.
MEM53@O01:S104@OG
Biv.
MEM53@O03:S104@OG
Bv.
MEM53@O01:WAT
Bvi.
MEM53@O03:WAT
Number of hbonds
Time (ns)
1
2
3
4
5
6
100
200
300
400
500

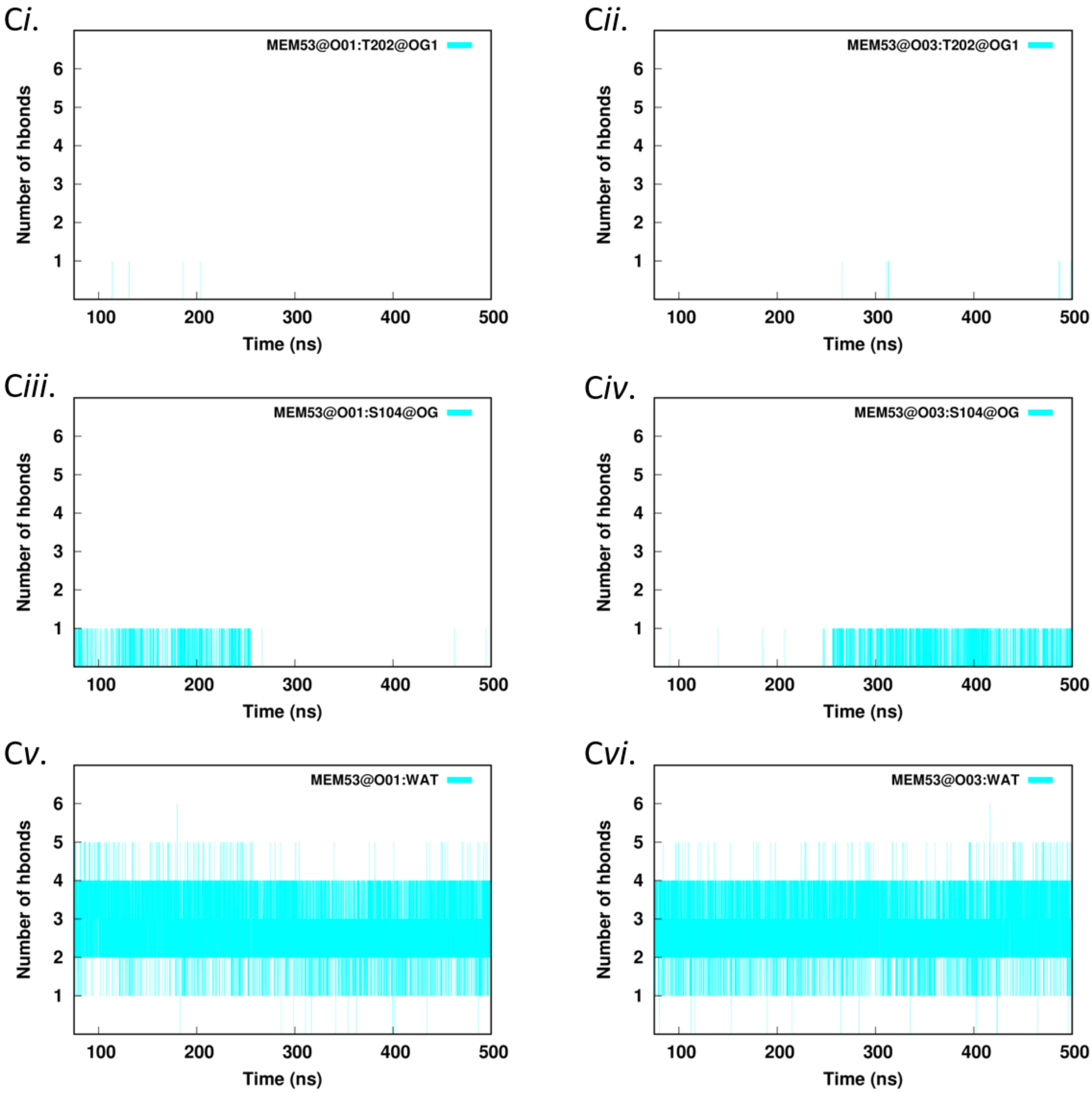
Ci.
MEM53@O01:T202@OG1
Number of hbonds
Time (ns)
Cii.
MEM53@O03:T202@OG1
Number of hbonds
Time (ns)
Ciii.
MEM53@O01:S104@OG
Number of hbonds
Time (ns)
Civ.
MEM53@O03:S104@OG
Number of hbonds
Time (ns)
Cv.
MEM53@O01:WAT
Number of hbonds
Time (ns)
Cvi.
MEM53@O03:WAT
Number of hbonds
Time (ns)

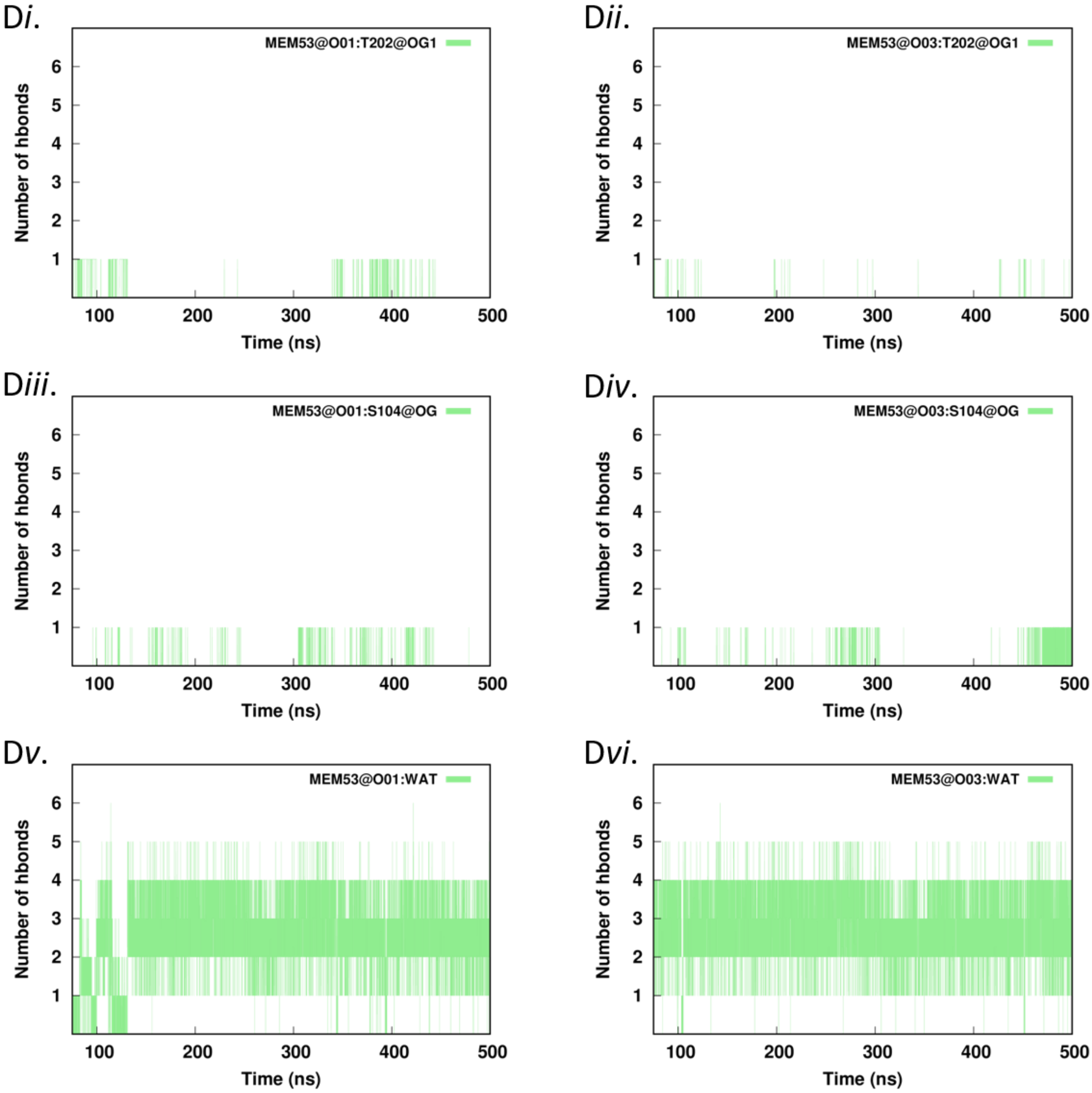

Di.
MEM53@O01:T202@OG1
Number of hbonds
Time (ns)
Dii.
MEM53@O03:T202@OG1
Number of hbonds
Time (ns)
Diii.
MEM53@O01:S104@OG
Number of hbonds
Time (ns)
Div.
MEM53@O03:S104@OG
Number of hbonds
Time (ns)
Dv.
MEM53@O01:WAT
Number of hbonds
Time (ns)
Dvi.
MEM53@O03:WAT
Number of hbonds
Time (ns)

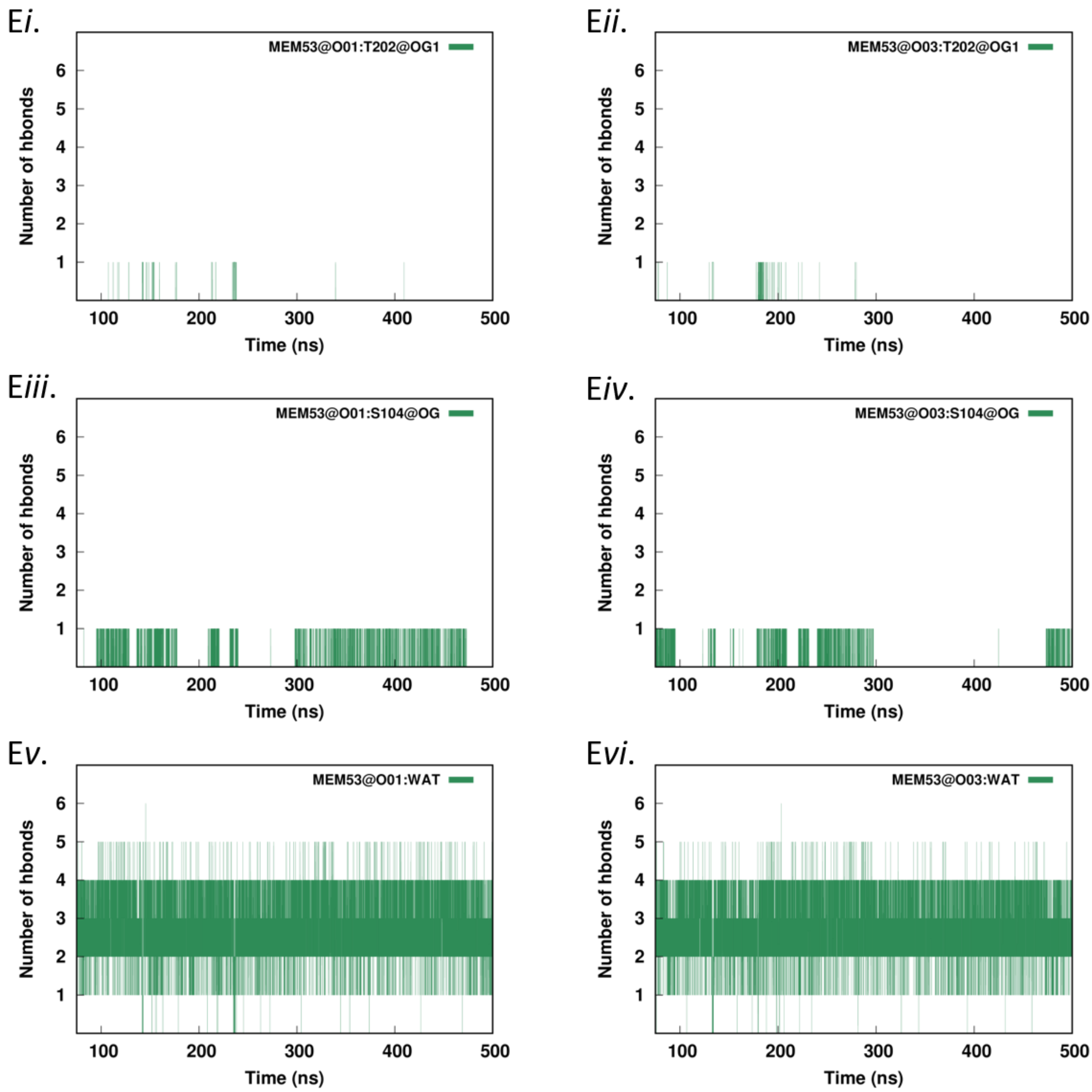

Figure S18. Time-Dependence of Hydrogen Bonds during MD Simulations of the OXA-57:Meropenem Complex. Time series of hydrogen bond interactions made by the meropenem carboxylate group through 425 ns MD trajectories of the OXA-57:meropenem complex simulations repeat one to five (R1-R5; panels A-E). (Residue number and atom name are given as acceptor:donor). *i*. MEM53@*O01*:T202@*OG1*, *ii*. MEM53@*O03*:T202@*OG1*, *iii*. MEM53@*O01*:S104@*OG*, *iv*. MEM53@*O03*:S104@*OG*, *v*. MEM53@*O01*:WAT, and *vi*. MEM53@*O03*:WAT. WAT = water molecules.

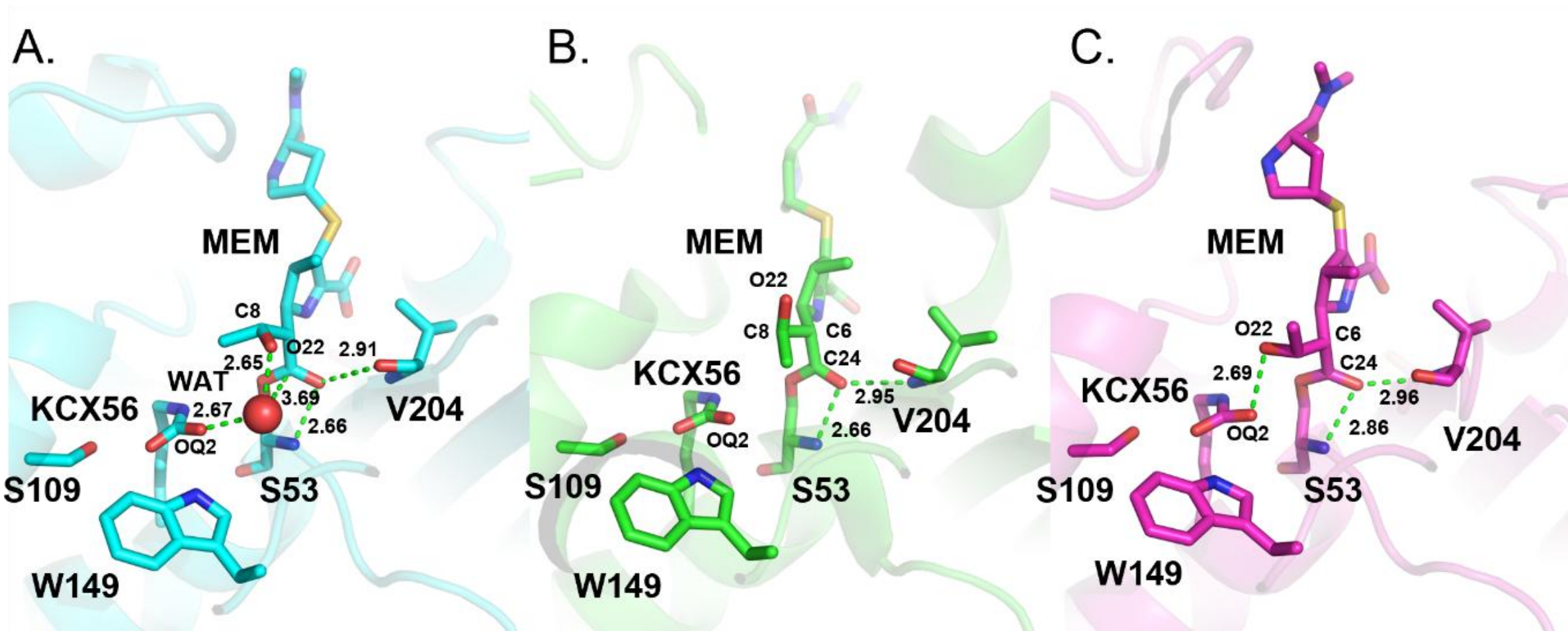

Figure S19. **Alternative Orientations of the Meropenem 6α-hydroxyethyl Group.** OXA-57:meropenem complexes with the 6α-hydroxyethyl group in A. orientation I (C24-C6-C8-O22 dihedral angle 69.8 °, snapshot from replicate MD simulation 4 after 75 ns; note presence of water molecule (WAT) in the deacylating position); B. orientation II (C24-C6-C8-O22 dihedral angle 160.8 °, crystal structure (9HPW); and C. orientation III (C24-C6-C8-O22 dihedral angle 283.4 °, snapshot from replicate MD simulation 1 after 100 ns). Carbon atoms colored cyan (A), green (B), magenta (C); all other atom colors as standard. This image was created in Pymol (www.pymol.org).

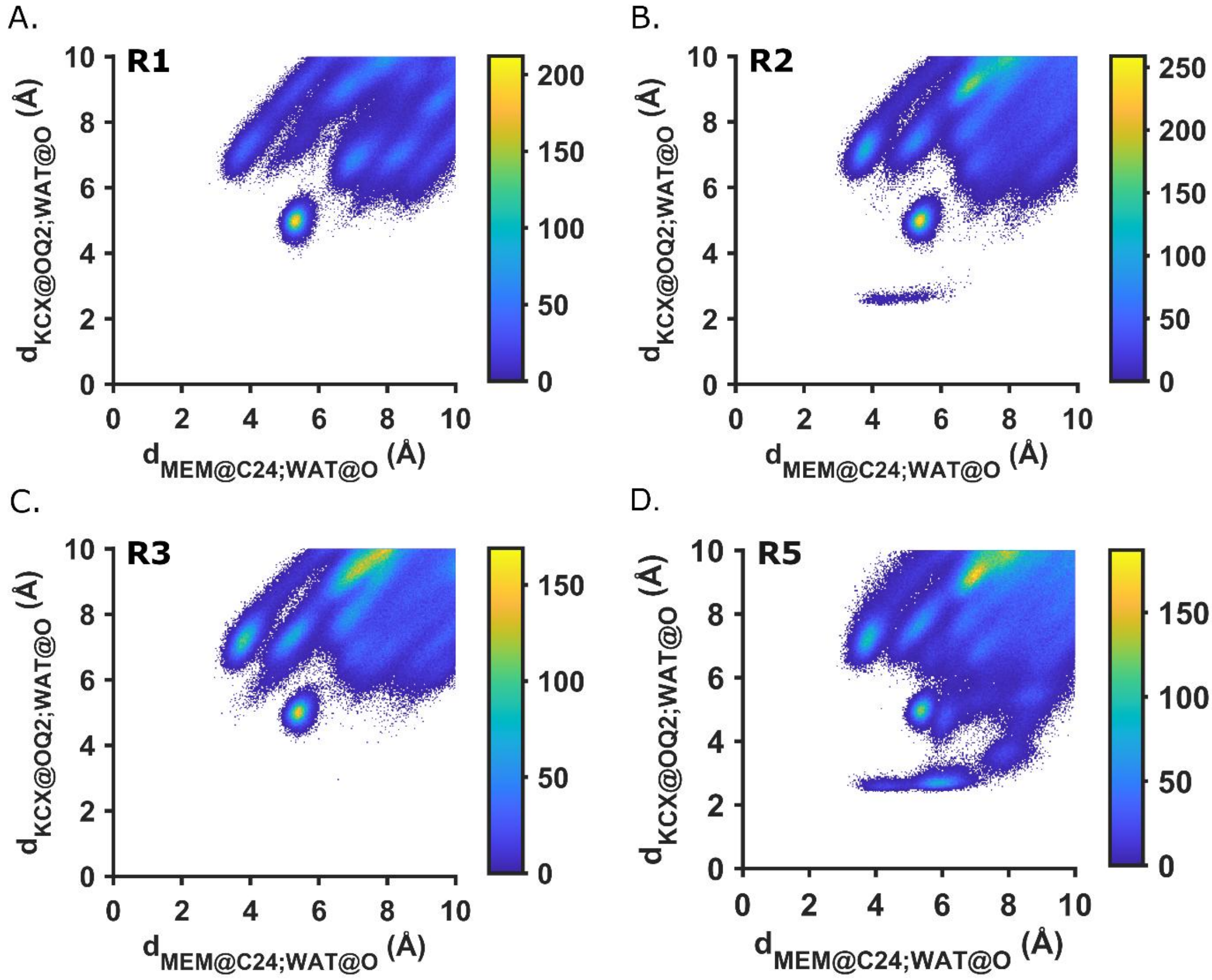

Figure S20. **Active Site Water Distribution during MD Simulations of the OXA-57:Meropenem Complex.** Distance distributions of water molecules between MEM53@*C24* and K56@*OQ2* over 425 ns MD trajectories of the OXA-57:meropenem complex in repeat simulations one, two, three and five (R1 - 3, R5; panels A - D).

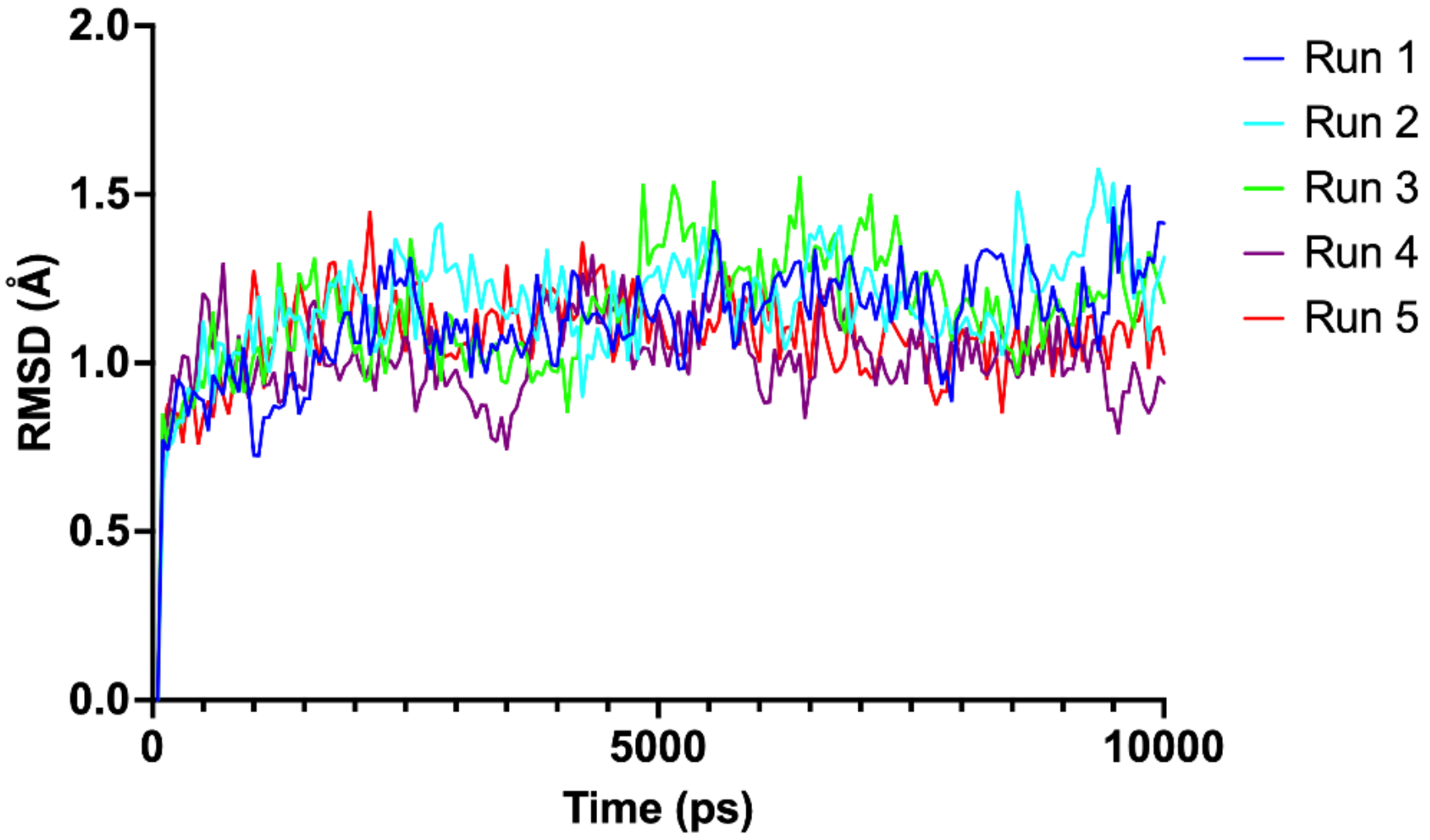

Figure S21. Stability of Molecular Dynamics Simulations of OXA-57:Meropenem Complex. Time series of Cα RMSD values, compared to the starting structure (snapshot from the R4 trajectory after 75 ns (**Figure S19A**), containing a water molecule in the putative deacylating position and the meropenem 6α-hydroxyethyl group in orientation I), over 5 x 10 ns simulations of the OXA-57:meropenem complex.

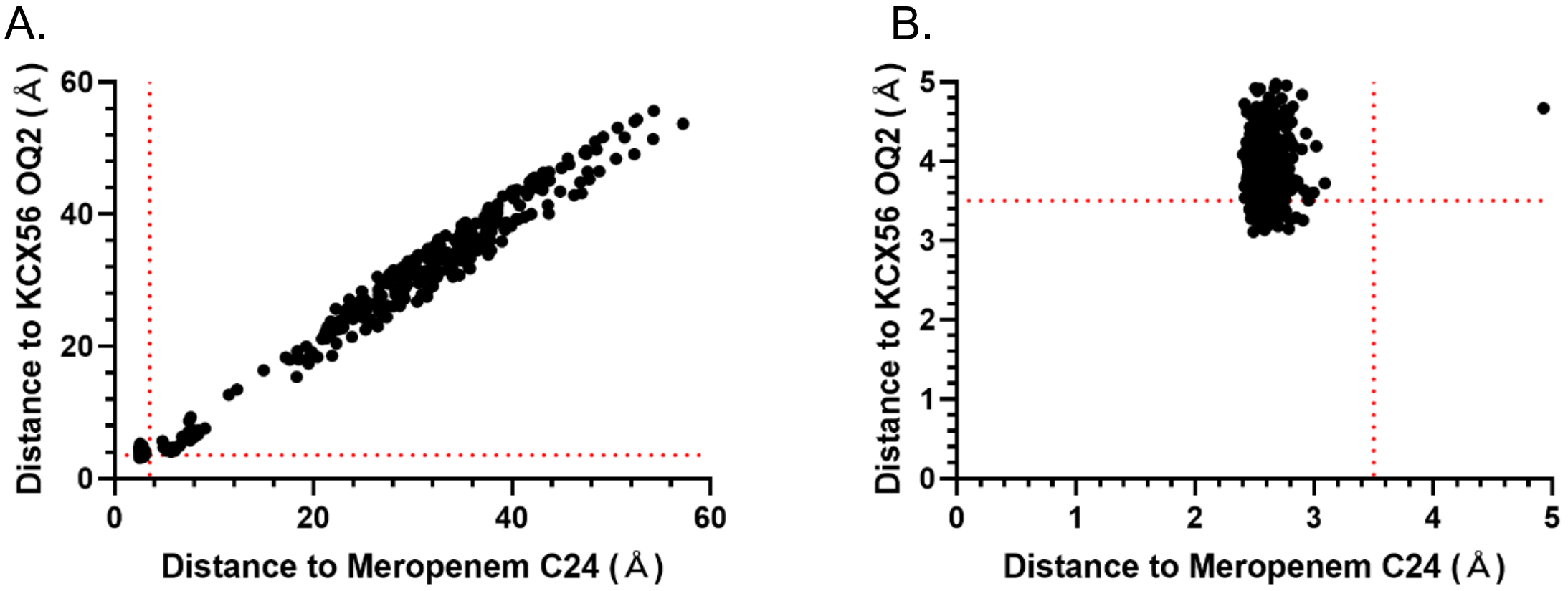

Figure S22. **Active Site Water Distribution during MD Simulations of the OXA-57:Meropenem Complex with Putative Deacylating Water Molecule.** Data are from MD simulations (5 x 10 ns, data points are 50 ps frames, 1000 frames total) of OXA-57:meropenem acylenzyme complex starting from a conformation with a water molecule in the deacylating position. Dotted red lines indicate water – carbamyl K56 OQ2 and water – meropenem C24 (carbonyl carbon) distances of 3.5 Å. A. all frames. B. frames (662) with water – carbamyl K56 OQ2 and water – meropenem C24 (carbonyl carbon) distances of ≤ 5.0 Å.

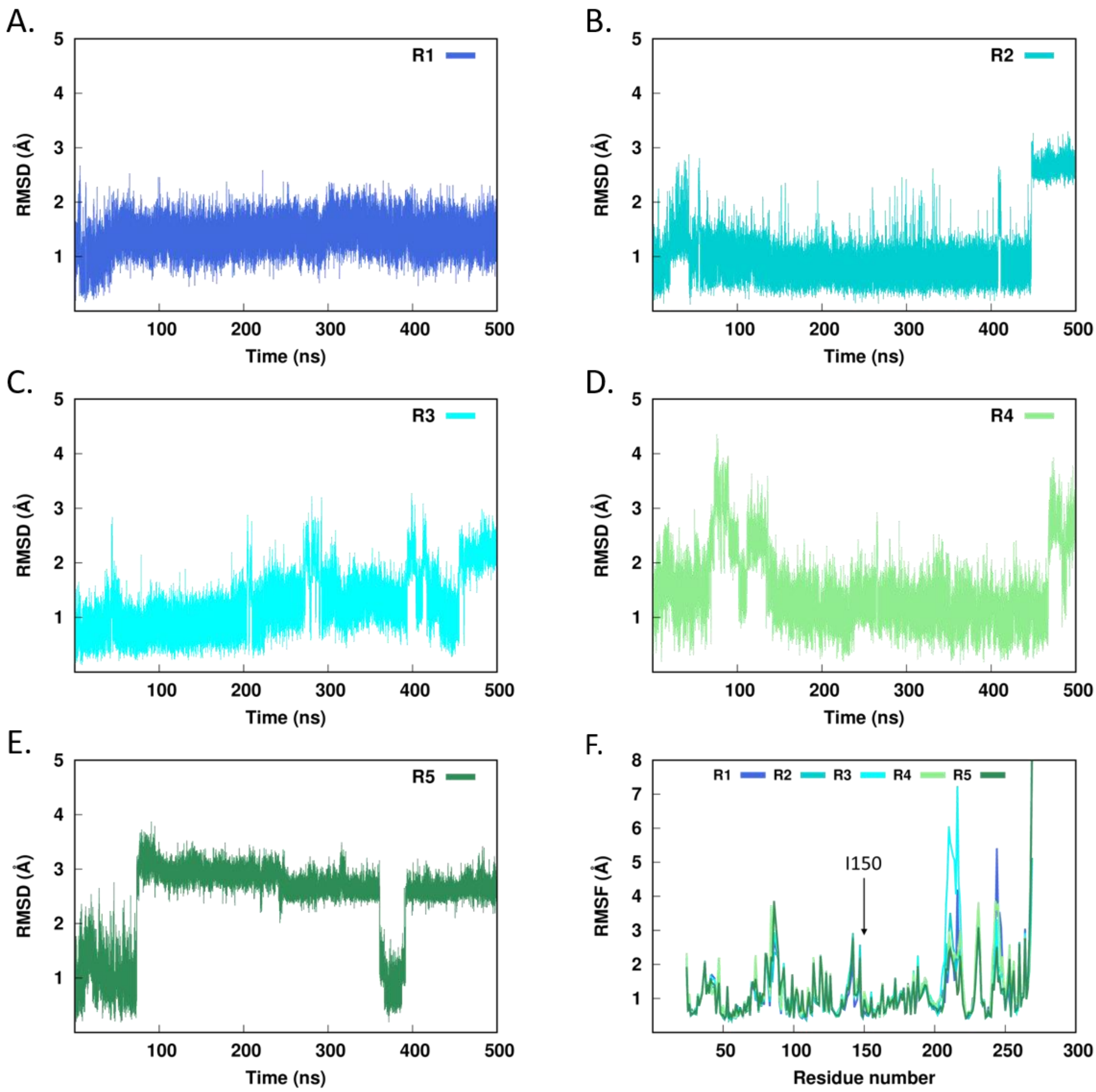

Figure S23. Mobility of Residue I150 during MD Simulations of the OXA-57:Meropenem Complex. RMSD distributions of the I150 sidechain (excluding atoms N, Cα, C, O, and hydrogens) for repeat simulations one to five (R1-R5; panels A-E) calculated over 500 ns MD trajectories of the OXA-57:meropenem complex. F. Per-residue RMSF distributions of all sidechains in the OXA-57:meropenem complex simulations, calculated over the final 425 ns of the MFD trajectories. I150 is annotated with a black arrow.

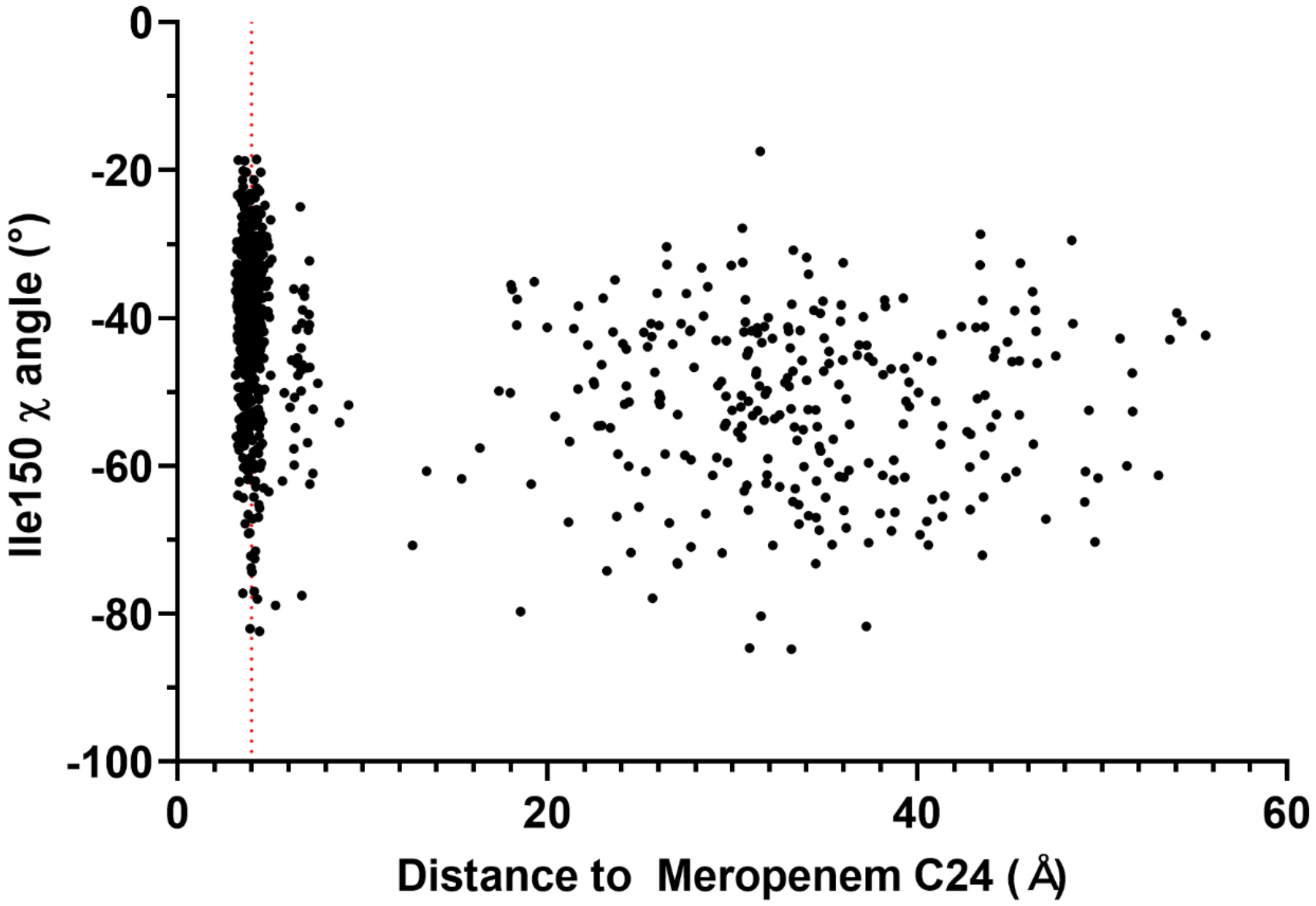

Figure S24. Mobility of Residue I150 during MD Simulations of the OXA-57:Meropenem Complex with a Water Molecule Positioned for Deacylation. Data are from MD simulations (5 x 10 ns) of OXA-57:meropenem acylenzyme complex starting from a conformation with a water molecule in the deacylating position. Dotted red line indicates water – meropenem C24 (carbonyl carbon) distance of 4.0 Å.

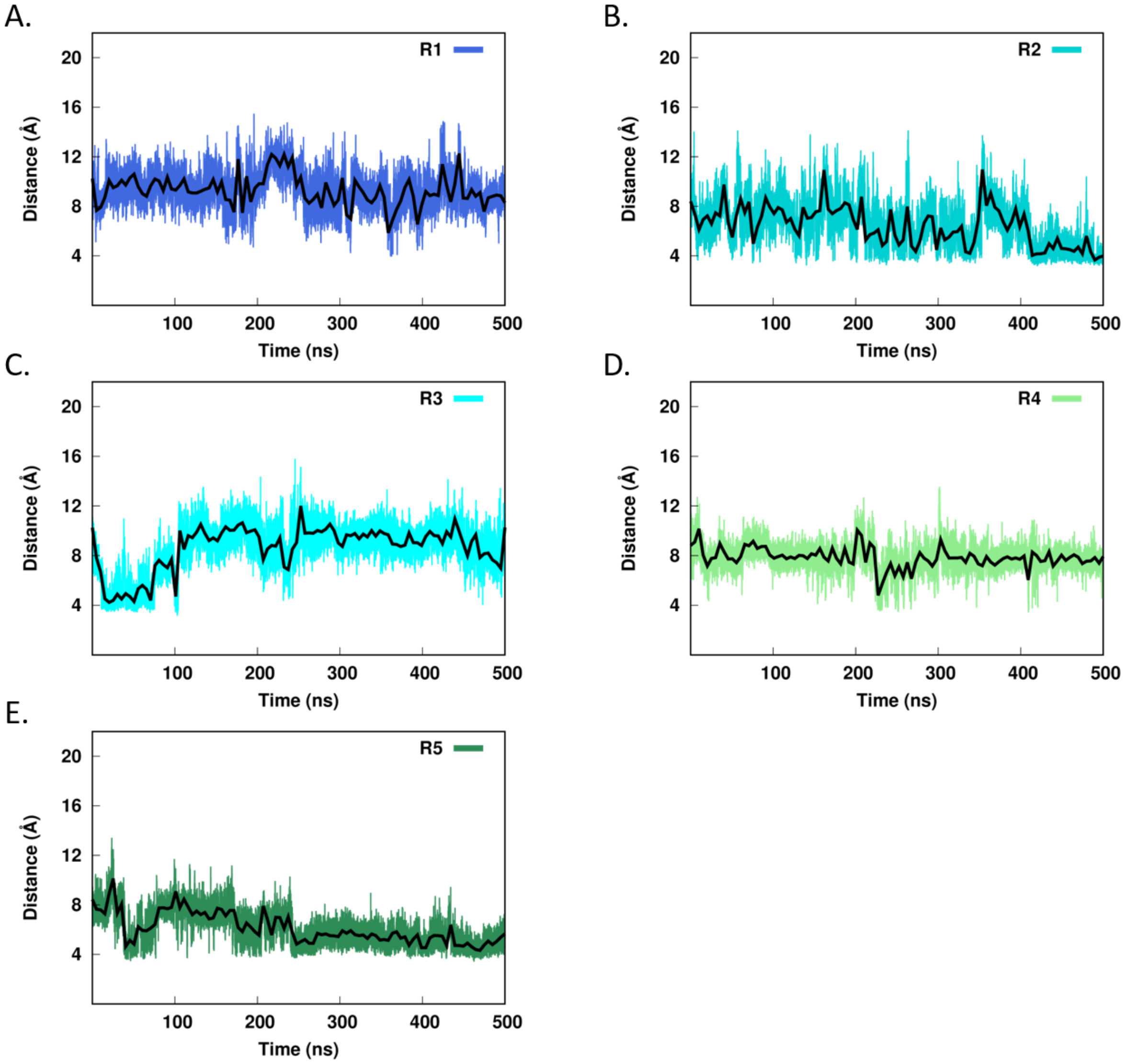

Figure S25. Mobility of Active Site Loops during MD Simulations of Uncomplexed OXA-57. Time series of the minimum distance between any carbon atom in residues W86 (α1 - α2 loop) and Y206 (β4 - β5 loop) calculated over 500 ns MD trajectories of uncomplexed OXA-57 for repeat simulations one to five (R1-R5; panels A-E). Black lines represent the distance data smoothed by the approximating cubic spline operation in gnuplot.

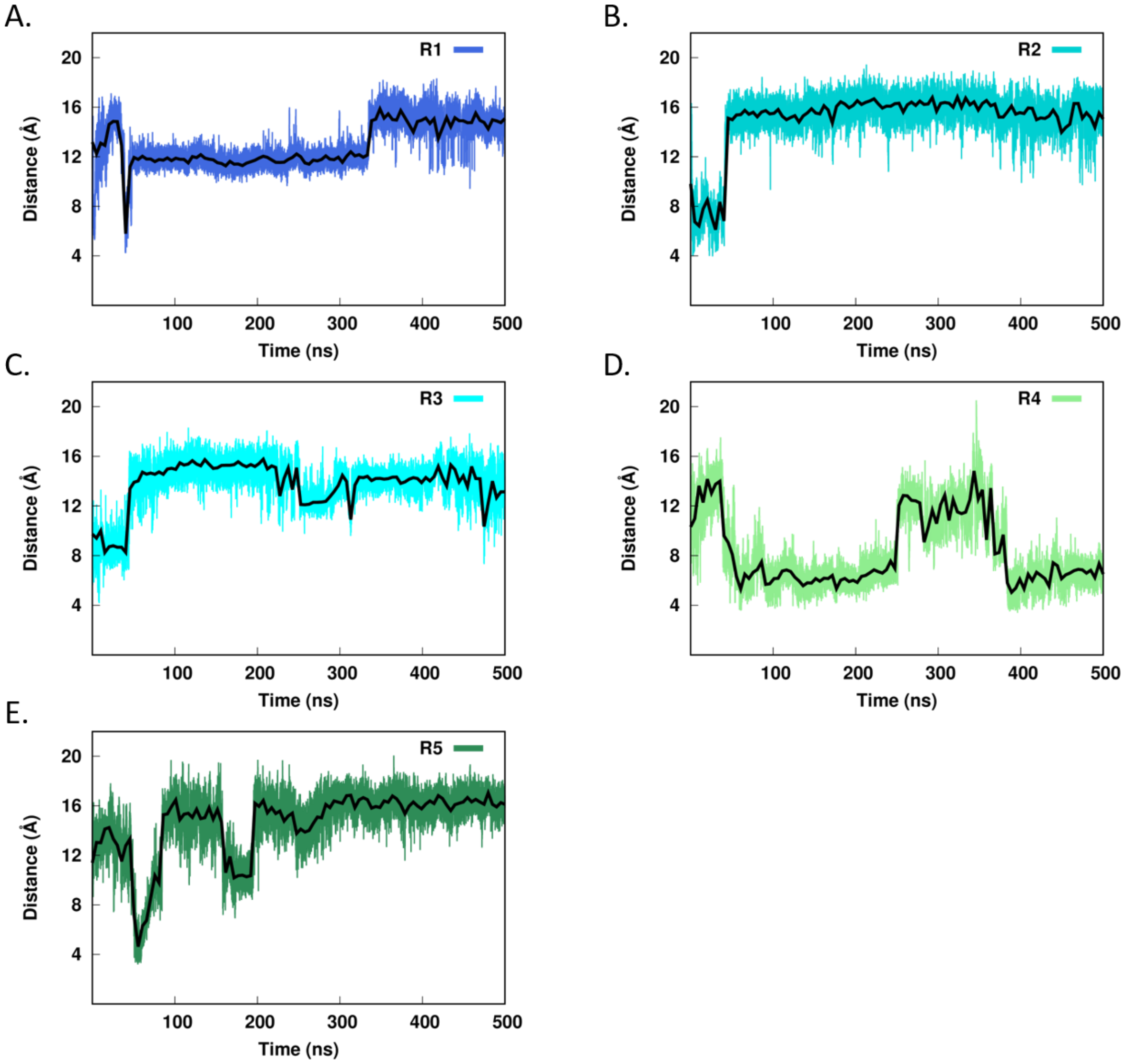

Figure S26. Mobility of Active Site Loops during MD Simulations of the OXA-57:Meropenem Complex. Time series of the minimum distance between any carbon atom in residues W86 (α1 - α2 loop) and Y206 (β4 - β5 loop) of the OXA-57:meropenem complex calculated over 500 ns MD trajectories for repeat simulations one to five (R1 – R5; panels A-E). Black lines represent the distance data smoothed by the approximating cubic spline operation in gnuplot.

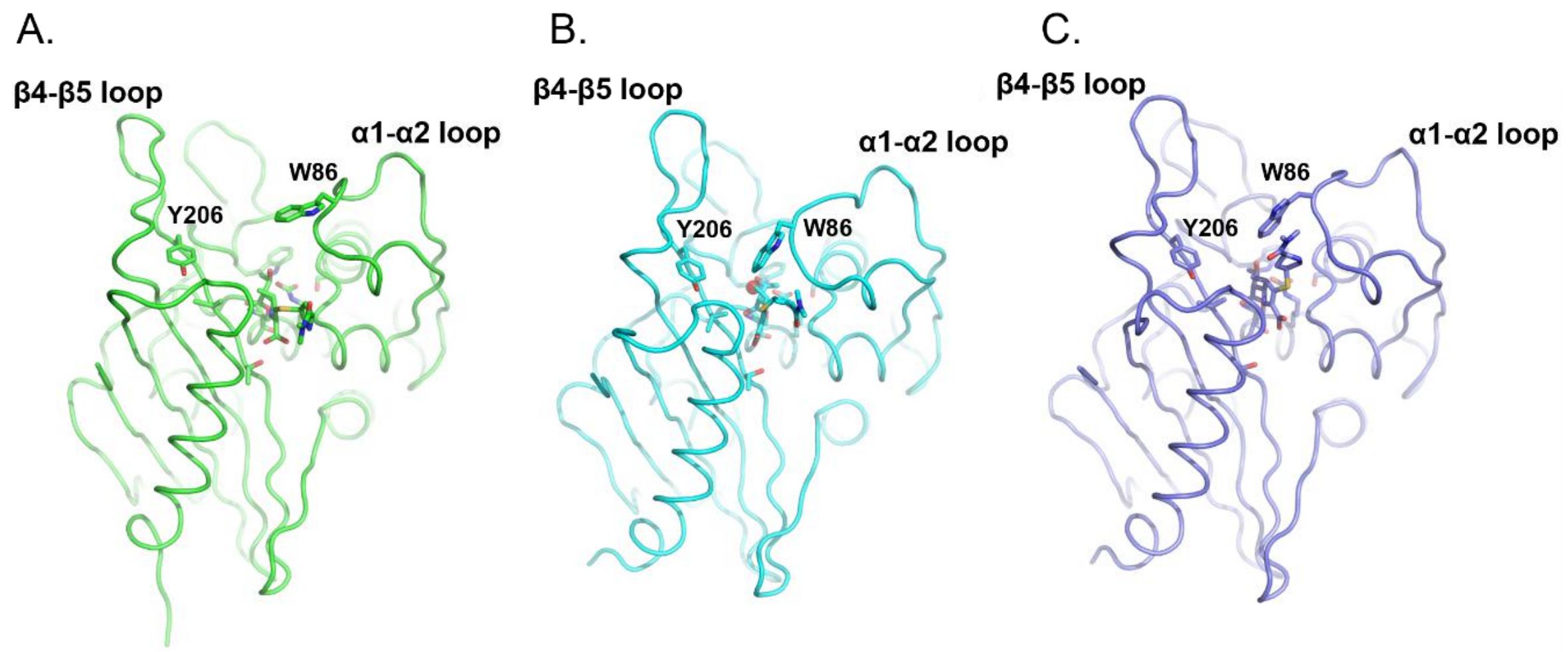

Figure S27. Orientations of Active Site Loops during Molecular Dynamics Simulations. Snapshots of OXA-57:meropenem complexes from molecular dynamics (MD) simulations showing A. starting model, B. snapshot from replicate MD simulation 4 after 75 ns; C. snapshot from end point of replicate MD simulation (500 ns). Note similar orientations of β4 - β5 loop in B. (where putative deacylating water molecule is present and meropenem 6α-hydroxyethyl group is in orientation I) and C. (where putative deacylating water molecule is absent and meropenem 6α-hydroxyethyl group is in orientation II). Carbon atoms colored green (A), cyan (B), slate (C); all other atom colors as standard. This image was created in Pymol (www.pymol.org).

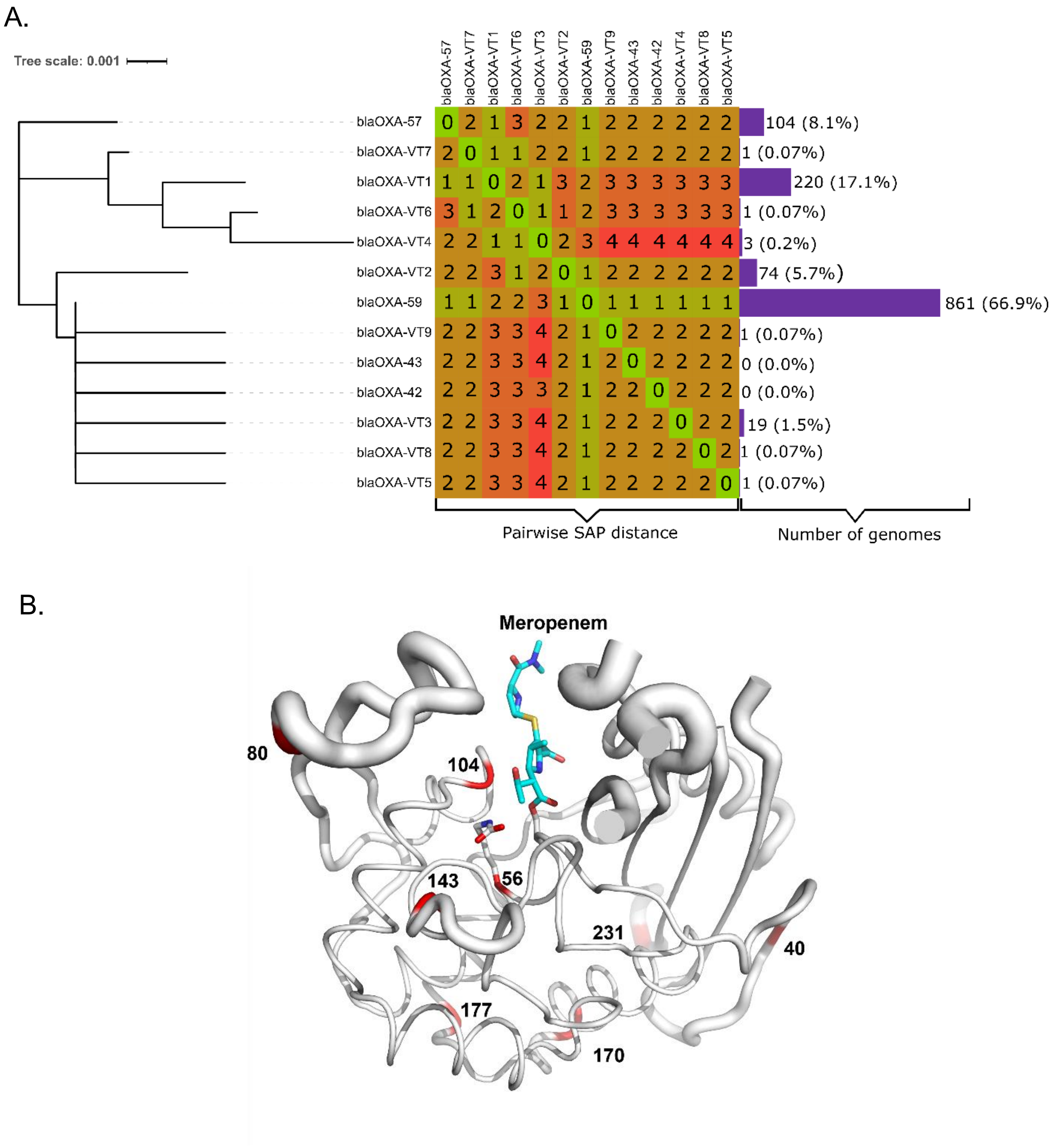

Figure S28. Sequence Polymorphisms in *B. pseudomallei* Chromosomal OXA β-Lactamases. A. Phylogenetic tree (left) and pairwise single amino acid polymorphism (SAP) distances (numbers are numbers of residue substitutions) of previously known blaOXA isoforms (OXA-42, OXA-43, OXA-57, OXA-59)) and new variant types (VTs, 1-9) identified in genomes of 1,286 clinical *B. pseudomallei* isolates collected in Northeast Thailand*(11)*. Magenta bars and numbers (right) denote absolute numbers and percentage frequency of occurrence of individual isoforms. Sequences are deposited in the European Nucleotide Archive (ENA) under study accession numbers PRJEB25606 and PRJEB35787. B. Positions of residue substitutions present in known and newly identified OXA isoforms (red) mapped onto crystal structure of OXA-57:meropenem complex (PDB 9HPW, this work). Bound meropenem (carbon atoms cyan) and the side chains of residues S53 and K56 (carbon atoms white) are shown as sticks. Worm thickness indicates B-factor. This image was created in Pymol (www.pymol.org).

References

(1.) Antunes, N. T., Lamoureaux, T. L., Toth, M., Stewart, N. K., Frase, H., and Vakulenko, S. B. Class D beta-lactamases: are they all carbapenemases?, *Antimicrob Agents Chemother* **2014**, *58* (4), 2119-2125.

(2.) Smith, C. A., Antunes, N. T., Stewart, N. K., Toth, M., Kumarasiri, M., Chang, M., Mobashery, S., and Vakulenko, S. B. Structural basis for carbapenemase activity of the OXA-23 beta-lactamase from Acinetobacter baumannii, *Chem Biol* **2013** *20* (9), 1107-1115.

(3.) Toth, M., Antunes, N. T., Stewart, N. K., Frase, H., Bhattacharya, M., Smith, C. A., and Vakulenko, S. B. Class D beta-lactamases do exist in Gram-positive bacteria, *Nat Chem Biol* **2016** *12* (1), 9-14.

(4.) Langley, G. W., Cain, R., Tyrrell, J. M., Hinchliffe, P., Calvopina, K., Tooke, C. L., Widlake, E., Dowson, C. G., Spencer, J., Walsh, T. R., Schofield, C. J., and Brem, J. Profiling interactions of vaborbactam with metallo-beta-lactamases, *Bioorg Med Chem Lett* **2019** *29* (15), 1981-1984.

(5.) Lohans, C. T., Wang, D. Y., Jorgensen, C., Cahill, S. T., Clifton, I. J., McDonough, M. A., Oswin, H. P., Spencer, J., Domene, C., Claridge, T. D. W., Brem, J., and Schofield, C. J. (13)C-Carbamylation as a mechanistic probe for the inhibition of class D beta-lactamases by avibactam and halide ions, *Org Biomol Chem* **2017** *15* (28), 6024-6032.

(6.) Philippon, A. M., Paul, G. C., and Jacoby, G. A. Properties of PSE-2 beta-lactamase and genetic basis for its production in Pseudomonas aeruginosa, *Antimicrob Agents Chemother* **1983** *24* (3), 362-369.

(7.) Poirel, L., Heritier, C., Tolun, V., and Nordmann, P. Emergence of oxacillinase-mediated resistance to imipenem in Klebsiella pneumoniae, *Antimicrob Agents Chemother* **2004** *48* (1), 15-22.

(8.) Tsivkovski, R., Totrov, M., and Lomovskaya, O. Biochemical Characterization of QPX7728, a New Ultrabroad-Spectrum Beta-Lactamase Inhibitor of Serine and Metallo-Beta-Lactamases, *Antimicrob Agents Chemother* **2020** *64,* No. e00130-20.

(9.) Krissinel, E. and Henrick, K. Secondary-structure matching (SSM), a new tool for fast protein structure alignment in three dimensions. *Acta Crystallogr., Sect. D* **2004**, *60* (12), 2256−2268.

(10.) Sali, A., and Blundell, T. L. Comparative protein modelling by satisfaction of spatial restraints, *J Mol Biol* **1993** *234* (3), 779-815.

(11.) Seng, R., Chomkatekaew, C., Tandhavanant, S., Saiprom, N., Phunpang, R., Thaipadungpanit, J., Batty, E. M., Day, N. P. J., Chantratita, W., West, T. E., Thomson, N. R., Parkhill, J., Chewapreecha, C., and Chantratita, N. Genetic diversity, determinants, and dissemination of Burkholderia pseudomallei lineages implicated in melioidosis in Northeast Thailand, *Nat Commun* **2024** *15* (1), 5699.
